# Evaluation of a two‐dimensional diode array for patient‐specific quality assurance of HyperArc

**DOI:** 10.1002/acm2.13438

**Published:** 2021-11-01

**Authors:** Richard A. Popple, Rodney J. Sullivan, Yuan Yuan, Xingen Wu, Elizabeth L. Covington

**Affiliations:** ^1^ Department of Radiation Oncology The University of Alabama at Birmingham Birmingham Alabama USA

**Keywords:** dosimetry, quality assurance, radiosurgery

## Abstract

**Purpose:**

To evaluate a two‐dimensional diode array for patient‐specific quality assurance of VMAT stereotactic radiosurgery (SRS) plans.

**Methods:**

The diode array, an SRS MapCHECK (SRSMC), was composed of a 77 mm ×77 mm face‐centered array having a spacing of 2.47 mm. Sixty SRS plans were selected from our clinical database, 30 for treatment of a single target and 30 for multiple targets. The target sizes ranged from 2.4 mm to 44.7 mm equivalent diameter (median 8.7 mm). The plans were delivered to the diode array. For multiple target plans, two measurements were obtained at two locations, one corresponding to the largest target and the other to the smallest target. Gamma using a 3%/1 mm criteria and the dose to the center diode were compared with radiochromic film (RCF). Dose to selected regions of the detector electronics was calculated.

**Results:**

The mean difference between the center diode and RCF was −1.2%. For a threshold of at least 95% of detectors/pixels having gamma < 1 for a 3%/1 mm criteria, SRSMC and RCF gave consistent results for 79 of the 90 measurements. For plans with an arc having a patient support angle of 90° or 270°, the median dose to the electronics was 0.65% of the prescription dose.

**Conclusions:**

SRSMC is an efficient tool for accurate patient‐specific quality assurance of VMAT single and multiple target radiosurgery, yielding similar clinical decisions as radiochromic film.

## 1 INTRODUCTION

The use of volumetric modulated arc therapy (VMAT) to deliver stereotactic radiosurgery (SRS) is becoming increasingly common.^1^, ^2^ Furthermore, planning techniques have been developed that produce high‐quality plans for simultaneous treatment of multiple lesions using a single isocenter.^3^, ^4^, ^5^ When combined with high dose rate flattening filter‐free beams, these plans can be delivered very efficiently, having treatment times similar to those for conventional fractionation.^6^

The efficiency of VMAT SRS has led vendors to develop systems that automate planning for multiple brain targets using a single isocenter (e.g., HyperArc, Varian Medical Systems, Palo Alto, CA; and BrainLab Multiple Metastasis Elements™, BrainLab, Munich, Germany).^7^ BrainLab Multiple Metastasis Elements™ is a linear accelerator agnostic planning system, whereas HyperArc is specifically for the TrueBeam Delivery System (Varian Medical Systems, Palo Alto, CA). HyperArc relies on a normal tissue objective (NTO) specific to multi‐target radiosurgery planning and allows automated delivery without a requirement for room entry to change couch angles.

For patient‐specific optimized plans, the current standard of care requires measurements to assess the accuracy of dose delivery.^8^, ^9^, ^10^, ^11^ Measurement of VMAT SRS plans is challenging because of the small target sizes. Small target sizes require a high‐resolution detector and a careful compensation for detector response in small field sizes.^12^ We used radiochromic film (RCF) for patient‐specific quality assurance of HyperArc plans.^13^ RCF has high resolution and, with careful calibration, can provide accurate dosimetry.^14^, ^15^, ^16^ However, RCF is labor‐intensive and requires delay between irradiation and analysis. Based on the work of Rose et al.^17^ and Ahmed et al.^18^, we hypothesized that a high‐resolution two‐dimensional diode array could be used to replace RCF for patient‐specific quality assurance of HyperArc plans.

## 2 METHODS

The SRS MapCHECK (SRSMC) (Sun Nuclear Corporation, Melbourne, FL) is composed of 1013 *n*‐type diodes having a volume of 0.007 mm^3^ (0.48 mm × 0.48 mm × 0.03 mm) arranged in a 77 × 77 mm^2^ face‐centered array. Each diode is spaced 2.47 mm from its four nearest neighbors. The array is enclosed in a 320 mm × 105 mm × 45 mm polymethyl methacrylate (PMMA) package.^17^ The SNC Patient software (version 8.2) corrects the diode measurements to account for pulse repetition rate, diode temperature, and angular dependence of response. These corrections have been reported to result in errors much less than 2% except under extreme conditions, most notably field sizes 5 mm or less.^18^

The SRSMC is used in conjunction with the StereoPHAN (Sun Nuclear Corporation, Melbourne, FL), a phantom composed of PMMA. The geometry of the StereoPHAN is a 15.2 cm diameter cylinder capped with a hemisphere of the same diameter. When inserted into the StereoPHAN, the center of the SRSMC detector array is located at the center of the cylinder and the hemisphere, 7.6 cm from the tip of the phantom. The phantom, and thus the array, can be rotated around the longitudinal axis; for this work, the array was fixed in the coronal plane of the phantom. For the creation of verification plans in the treatment planning system (TPS), a digital phantom was created using MATLAB (MathWorks, Natick, MA) composed of empty CT images with 1 mm spacing and a structure set containing the external contour of the phantom. The phantom was stored as DICOM files and imported into the Eclipse TPS (Varian Medical Systems, Palo Alto, CA). In Eclipse, the CT value inside the external contour was set to the relative electron density of PMMA (1.19) and assigned the material PMMA.^17^ The DICOM files for the digital phantom are provided in the supplementary materials.

HyperArc is a highly automated system for treatment planning and delivery of intracranial radiosurgery. It comprises a dedicated planning tool in the Eclipse TPS and automated delivery on the linear accelerator. The system uses VMAT using up to 4 arcs selected from table angles 0°, 45°, 315°, and either 90° or 270°.

The patient‐specific quality assurance protocol for HyperArc at our institution used EBT‐XD RCF (Ashland Global Holdings Inc., Wilmington, Delaware).^13^ A strip of film 5 cm × 20 cm was placed in the coronal plane of an 18 cm × 17 cm × 20 cm PMMA slab phantom, with pins for marking the film to establish the phantom coordinate system. For multiple target plans, two verification plans were created corresponding to the largest and the smallest target volumes. For each verification plan, the isocenter was placed such that the center of the target of interest was located at the origin of the phantom coordinate system. At the start of each measurement session, the phantom was positioned using the on‐board kilovoltage imaging system. For each measurement, the phantom was shifted according to the verification plan. Calibration curves for RCF were obtained for each measurement session using a stepped dose pattern generated using a standard MLC field‐in‐field pattern.^14^ The RCF was digitized at 96 dpi using a flat‐bed scanner (Perfection V700, Epson America Inc., Long Beach, CA) operated in the transmission mode and 16‐bits per color channel. The calibration film pixel values for each color channel were fit to dose using a three‐parameter function and the patient‐specific film images were converted to dose using a three‐channel technique.^15^

Sixty HyperArc VMAT SRS plans were selected from our clinical database. Thirty plans were for the treatment of a single target and thirty plans were for the treatment of multiple targets. The target sizes ranged from 2.4 mm to 44.7 mm equivalent diameter (median 8.7 mm), where the equivalent diameter is the diameter of a sphere having the same volume as the target. All treatment plans used the 10 MV flattening filter‐free beam (2400 MU/min maximum dose rate) on an Edge linear accelerator equipped with an HD120 multileaf collimator (Varian Medical Systems, Palo Alto, CA). Every plan had at least one non‐coplanar arc, and 20 plans had an arc in the sagittal plane (table angle 90° or 270°). The dose was calculated using 1 mm grid spacing and either the AAA algorithm version 13.6.23 (23 plans) or AcurosXB version 15.5.11 (37 plans) (Varian Medical Systems, Palo Alto, CA).

**FIGURE 1:**
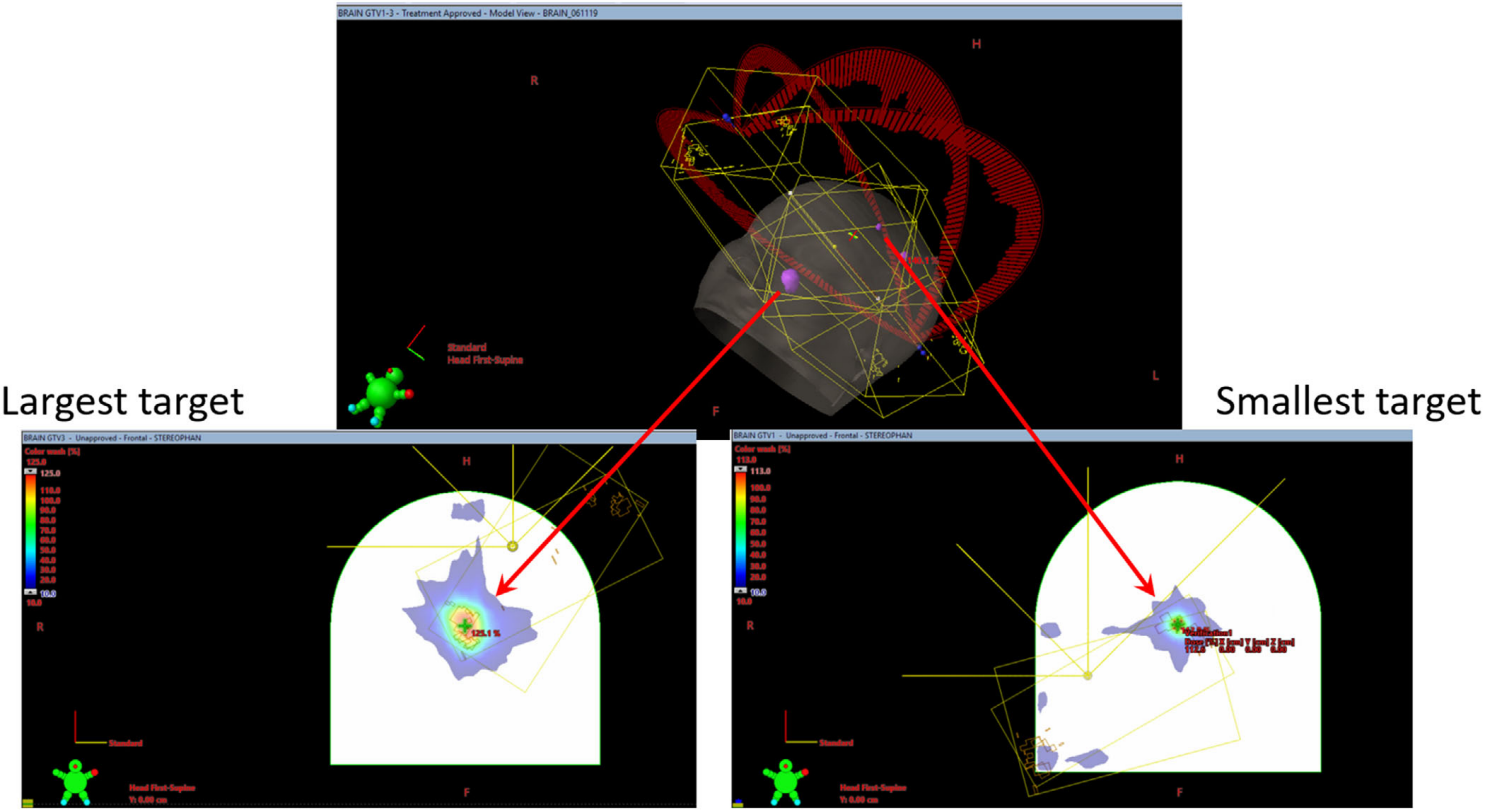
Schematic of verification planning for multiple‐target plans. For each treatment plan, two verification plans were created such that the center of the largest and smallest targets were located at the origin of the phantom coordinate system

The SRSMC measurement process was analogous to the RCF protocol. The same isocenter position as the corresponding RCF verification plan was used for the SRSMC verification plan, thus centering the dose distribution for the target of interest on the center detector of the diode array. The planning process is illustrated in Figure 1. At the start of a measurement session, anteroposterior (AP) and lateral views were acquired using the on‐board kilovoltage imaging system and the position was adjusted using the detector plane, the edge rows and columns of the array, and the location of the central (CAX) diode. For each measurement, the phantom was shifted according to the verification plan using automated couch movement available at the control console. A dose calibration using a 4 cm ×4 cm field was done before each measurement session.

The SRSMC measurements were analyzed using SNC Patient version 8.2 and the RCF measurements were analyzed using in‐house software developed in MATLAB (The MathWorks, Inc., Natick, MA). The CAX dose was compared with the average dose in a 1 mm^2^ region of interest for the corresponding RCF measurement. Gamma calculations^19^ were done for both SRSMC and RCF using criteria 3%/1 mm, a 10% dose threshold, and global normalization. The in‐house software interpolated the TPS dose calculations onto a 0.1 mm grid for the gamma calculation. SNC patient used the simplex method described by Ju et al.^20^ Because the RCF measurements were done using strips of film 5 cm wide, the gamma analysis region of interest was limited to a 20 mm radius from the origin of the phantom coordinate system.

To evaluate the dose to the electronics outside of the active measurement area, the synthetic phantom was extended to include the electrometer and readout electronics. Regions‐of‐interest (ROIs) 10 mm × 10 mm were contoured centered around component ROIs identified by Sun Nuclear product engineering. The ROIs included two electrometers, an amplifier, a voltage reference, and communication electronics. The mean and maximum doses to the ROIs were calculated.

## 3 RESULTS

**FIGURE 2:**
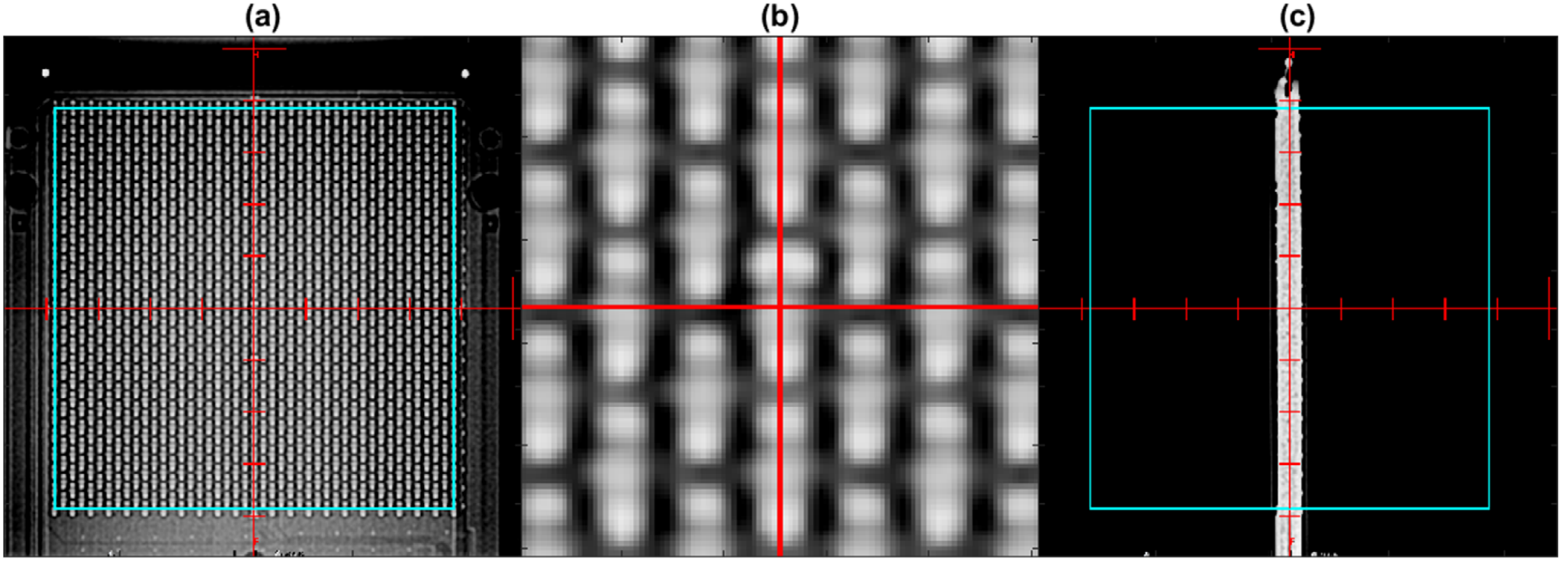
A 2 kV image‐guided setup images of the SRS MapCHECK (SRSMC). (a) Anteroposterior (AP) view, (b) magnified AP view, and (c) lateral view

The imaging technique for the orthogonal alignment images was 80 kVp and 5 mAs. An image filter was applied to enhance the contrast of the diodes. Of the filters available on the Edge on‐board imaging system, “Dynamic Filter” and “Highlight” were found to be useful for visualizing the diode array on the AP image. A 7.7 cm aperture representing the extent of the diode array was superimposed on the AP image, the corners of which were aligned to the corresponding detectors. The AP image was then magnified to further refine the alignment of the isocenter to the center diode. It was noted that the superior and inferior ends of each diode were brighter than the center, which was presumed to be the solder connections. The diodes could not be visualized on the lateral image, so the vertical position of the measurement plane could not be determined directly. However, a fiducial marker located superior to the array was used to evaluate the vertical position of the array. Example orthogonal alignment images are shown in Figure 2.

**FIGURE 3:**
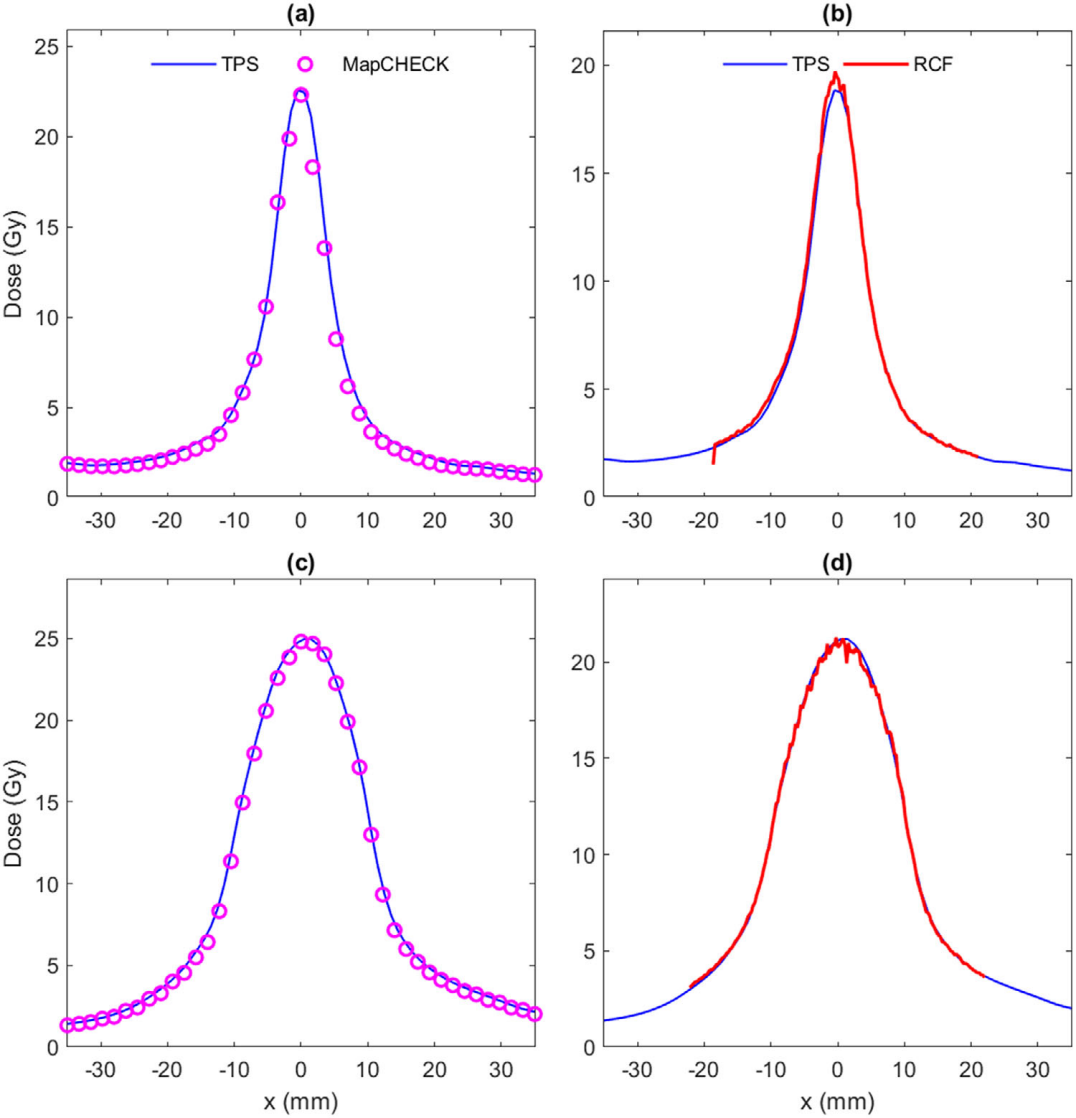
Measured and calculated dose profiles through the phantom center for the treatment plan illustrated in Figure 1 for (a) the smallest target measured with SRS MapCHECK (SRSMC), (b) the smallest target (volume 0.1 cm^3^, equivalent diameter 5.8 mm) measured with radiochromic film, (c) the largest target (volume 4.1 cm^3^, equivalent diameter 19.9 mm) measured with SRSMC, and (d) the largest target measured with radiochromic film. Note that the treatment planning system calculations and dose scales are different because SRSMC and radiochromic film measurements used different phantoms

**FIGURE 4:**
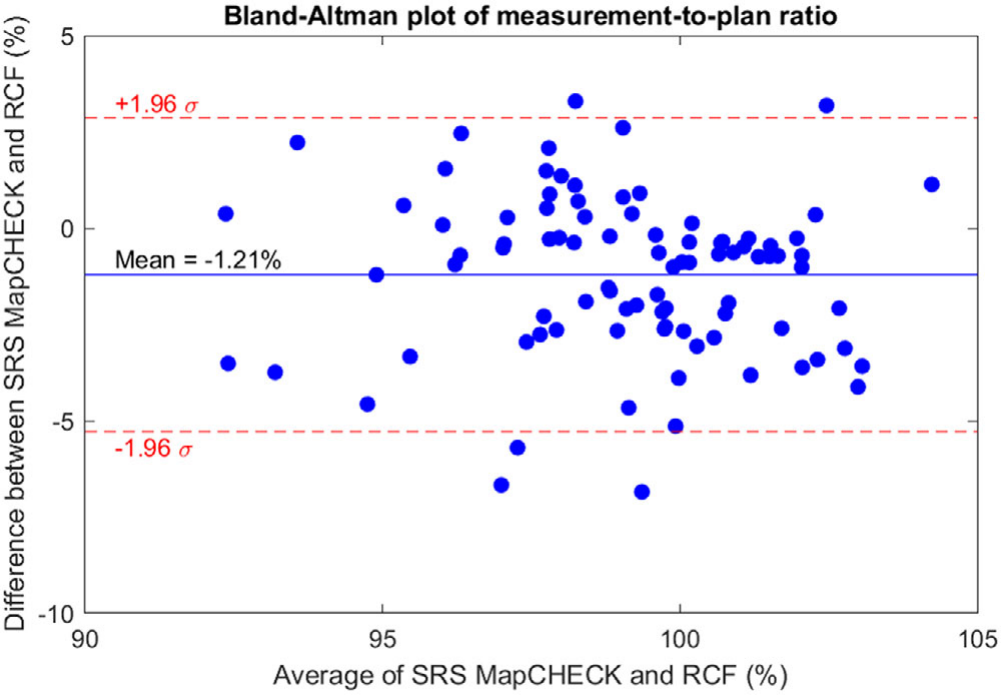
Bland–Altman plot of measurement‐to‐plan ratio for the center diode (CAX diode) of the SRS MapCHECK (SRSMC) and radiochromic film (RCF) at the center of the phantom

Dose profiles for all 90 measurements are shown in the supplementary materials. Example dose profiles for the SRSMC and RCF are shown in Figure 3 for the treatment plan illustrated in Figure 1. A Bland–Altman plot comparing the CAX diode with RCF is shown in Figure 4. The mean difference between the CAX diode and the RCF was −1.2% (95% confidence interval −1.6% to −0.8%). There was no difference between single‐target and multiple‐target plans (two‐sample *t*‐test *p* = 0.93).

**FIGURE 5:**
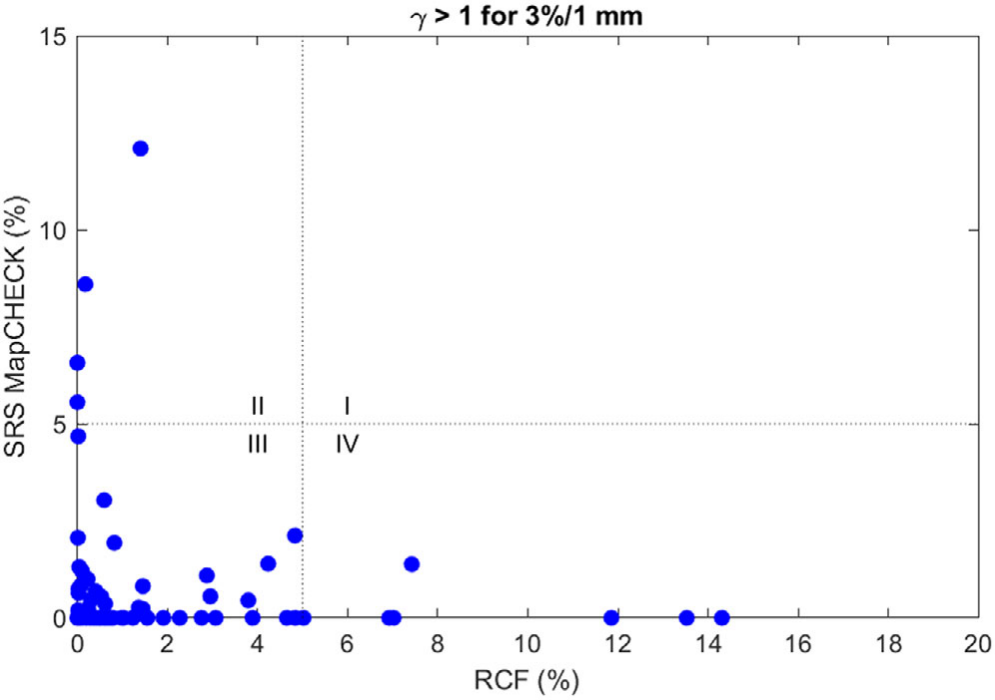
Comparison of gamma failure rates for SRS MapCHECK (SRSMC) and radiochromic film (RCF). For quadrants III and IV, the passing rate is ≥95% for SRSMC. For quadrants II and III, the passing rate is ≥95% for RCF. SRSMC and RCF agree (both pass or both fail) for quadrants I and III and disagree in quadrants II and IV

Clinical decision‐making using the gamma index is based on the number of pixels/detectors having a gamma index ≤ 1. The tolerance limit recommended by the AAPM task group 218 is gamma passing rate ≥ 95% with 3%/2 mm and a 10% dose threshold.^11^ However, task group 218 also recommended using a more stringent criteria for SRS. A recent study of action limits for SRS concluded that the spatial tolerance criteria could be reduced to 1 mm^21^, therefore we used 3%/1 mm and a 10% dose threshold. The number of measurements having ≥95% passing rate was 85 (94.4%) and 82 (91.1%) for SRSMC and RCF, respectively, and 78 (86.7%) measurements had ≥95% passing rate for both SRSMC and RCF. A comparison between the gamma pass rates for SRSMC and RCF is shown in Figure 5. Figure 5 is divided into four quadrants. In quadrants III and IV, the passing rate is ≥95% for SRSMC. In quadrants II and III, the passing rate is ≥95% for RCF. In quadrant I, both SRSMC and RCF have passing rates < 95%. Seven plans met the criteria for SRSMC but not for RCF. For six of these measurements (2, 15, 56, 65, 80, and 81 in the supplementary material), the pass rates were < 95% due to dose discrepancies for which the RCF dose was greater than TPS in the high‐dose region of the target. For the seventh measurement (59 in the supplementary material), the pass rate was < 95% due to dose discrepancies in the low‐dose region, suggesting an error in the calibration of the film. Both measurements for two multiple‐target plans (measurements 10, 11, 49, 50 in the supplementary material) met the criteria for RCF but not for SRSMC. All were for small targets having equivalent diameters 2.4, 2.8, 3.8, and 5.8 mm, for which the SRSMC reported dose at the center of the phantom lower than RCF −4.6%, −3.7%, −3.5%, and −3.3%, respectively.

**FIGURE 6:**
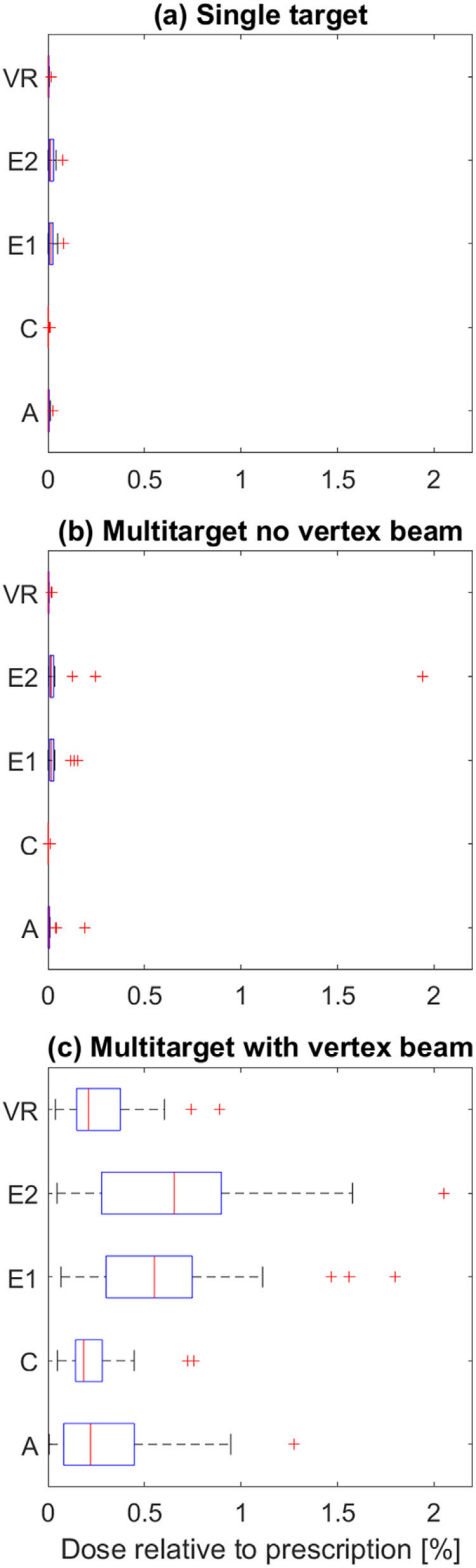
Box and whisker plots of calculated dose to the amplifier (A), communication (C), electrometer 1 (E1), electrometer 2 (E2), and voltage reference (VR) electronic locations

The box plots of the dose received by the electronics are shown in Figure 6 for three groups of plans, plans having a single target (*N* = 30), plans having multiple targets without a sagittal arc (*N* = 10), and plans having multiple targets with a sagittal arc (*N* = 20). The largest median dose to all the ROIs is less than 0.02% of the prescription dose for plans without a sagittal arc (patient support angle 90° or 270°). For plans with a sagittal arc, the median dose is much larger, with electrometer 2 receiving the highest median dose 0.65% of the prescription dose.

## 4 DISCUSSION

A significant advantage of a two‐dimensional diode array compared to the film is the efficiency of use and the immediate availability of results after measurement. Furthermore, RCF requires careful calibration and processing protocols to achieve accurate results. This study demonstrated that a two‐dimensional diode array can obtain results equivalent to the film, resulting in similar gamma analysis outcomes using tolerance limits appropriate for SRS.

RCF agreed well with a plastic scintillator detector,^22^ so the systematic 1.2% difference between the SRSMC and RCF suggests a difference in response of the diode array due to the non‐coplanar VMAT delivery of patient plans and the coplanar calibration geometry. Further investigation is needed to determine if this difference is consistent across multiple diode arrays and if an improved calibration technique can be developed to reduce the difference.

The SRSMC is suitable for routine patient‐specific quality assurance of VMAT, multiple‐target radiosurgery treatment plans. However, given the systematic difference in absolute dose at the central diode and the courser resolution compared to film, the SRSMC should be supplemented with other tools when commissioning a VMAT SRS program. Because RCF, gel dosimetry, and other high‐resolution detectors require specialized equipment and expertise, it is challenging for most clinics to use them. Therefore, clinics should take advantage of end‐to‐end testing services provided by third parties, such as the M. D. Anderson Dosimetry Calibration Laboratory, when commissioning a VMAT radiosurgery program.

The application of several diode arrays, including the SRSMC, to SRS PSQA has recently been reported by Xia et al.^21^ Xia et al. investigated a range of spatial/dose criteria for gamma analysis and found that the spatial tolerance criteria could be reduced to 1 mm. They specifically concluded that 3%/1 mm could be applied for the SRSMC. Using the suggested 3%/1 mm, we found that SRSMC agreed well with RCF.

This work focused on the measurement of individual targets at the center of the diode array. Simultaneous measurement of multiple targets is challenging because it is uncommon for multiple targets to be in the same plane. The SRSMC can be rotated to place at least two targets in a common plane, but this technique significantly increases the complexity of the measurement process. Investigation of the SRSMC for simultaneous measurement of multiple targets is a potential topic for future work.

Our RCF technique used strips of film that were 5 cm wide. This technique eliminated the need for a correction to account for the lateral response of the film scanner.^15^ However, for larger targets, the dose distribution extended beyond the limits of the film. Because the SRSMC is 7.7 cm wide, it is more suitable for larger targets.

The SRSMC electronics are distant from the high‐dose region and are not directly irradiated during most of a VMAT delivery. The calculation accuracy of out‐of‐field dose by TPSs is significantly less accurate than in the treated volume,^23^ so the calculations presented here have significant uncertainty. However, it is evident that the dose to the electronics is small but non‐negligible, particularly for irradiation at table angles 90° or 270°. The effect of this dose on the long‐term performance of the device remains to be seen. In our clinical practice, we have been using the SRSMC clinically and have irradiated it more than 450 times totaling more than 6000 Gy, of which treatment plans containing vertex beams accounted for 1860 Gy (31%). To date, we have not observed any failures of diodes or electronics.

## 5 CONCLUSION

The SRSMC is an efficient tool for accurate patient‐specific quality assurance of VMAT single and multiple‐target radiosurgery. For a 3%/1 mm tolerance level, the SRSMC yields similar clinical decisions as radiochromic film.

## AUTHOR CONTRIBUTIONS

Richard A. Popple made substantial contributions to the conception and design of the work; the acquisition, analysis, and interpretation of data for the work; drafting the work and revising it critically for important intellectual content; gave final approval of the version to be published; and agrees to be accountable for all aspects of the work in ensuring that questions related to the accuracy or integrity of any part of the work are appropriately investigated and resolved. Rodney J. Sullivan made substantial contributions to the interpretation of data for the work; revising it critically for important intellectual content; gave final approval of the version to be published; and agrees to be accountable for all aspects of the work in ensuring that questions related to the accuracy or integrity of any part of the work are appropriately investigated and resolved. Yuan Yuan made substantial contributions to the interpretation of data for the work; revising it critically for important intellectual content; gave final approval of the version to be published; and agrees to be accountable for all aspects of the work in ensuring that questions related to the accuracy or integrity of any part of the work are appropriately investigated and resolved. Xingen Wu made substantial contributions to the interpretation of data for the work; revising it critically for important intellectual content; gave final approval of the version to be published; and agrees to be accountable for all aspects of the work in ensuring that questions related to the accuracy or integrity of any part of the work are appropriately investigated and resolved. Elizabeth L. Covington made substantial contributions to the conception and design of the work; the acquisition, analysis, and interpretation of data for the work; revising it critically for important intellectual content; gave final approval of the version to be published; and agrees to be accountable for all aspects of the work in ensuring that questions related to the accuracy or integrity of any part of the work are appropriately investigated and resolved.

## Supporting information

Supporting information

Supporting information

## References

[1] Mayo CS , Ding L , Addesa A , Kadish S , Fitzgerald TJ , Moser R . Initial experience with volumetric IMRT (RapidArc) for intracranial stereotactic radiosurgery. Int J Radiat Oncol Biol Phys. 2010;78(5):1457‐1466. 20207494 10.1016/j.ijrobp.2009.10.005

[2] Wolff HA , Wagner DM , Christiansen H , Hess CF , Vorwerk H . Single fraction radiosurgery using rapid arc for treatment of intracranial targets. Radiat Oncol. 2010;5:77. 20836871 10.1186/1748-717X-5-77 PMC2949676

[3] Thomas EM , Popple RA , Wu X , et al. Comparison of plan quality and delivery time between volumetric arc therapy (RapidArc) and gamma knife radiosurgery for multiple cranial metastases. Neurosurgery. 2014;75(4):409‐417. discussion 417‐408. 24871143 10.1227/NEU.0000000000000448 PMC4203364

[4] Clark GM , Popple RA , Young PE , Fiveash JB . Feasibility of single‐isocenter volumetric modulated arc radiosurgery for treatment of multiple brain metastases. Int J Radiat Oncol Biol Phys. 2010;76(1):296‐302. 19836151 10.1016/j.ijrobp.2009.05.029

[5] Clark GM , Popple RA , Prendergast BM , et al. Plan quality and treatment planning technique for single isocenter cranial radiosurgery with volumetric modulated arc therapy. Pract Radiat Oncol. 2012;2(4):306‐313. 24674169 10.1016/j.prro.2011.12.003

[6] Prendergast BM , Popple RA , Clark GM , et al. Improved clinical efficiency in CNS stereotactic radiosurgery using a flattening filter free linear accelerator. J Radiosurg SBRT. 2011;1:117‐122. 29296305 PMC5675468

[7] Liu H , Thomas EM , Li J , et al. Interinstitutional plan quality assessment of 2 linac‐based, single‐isocenter, multiple metastasis radiosurgery techniques. Adv Radiat Oncol. 2020;5(5):1051‐1060. 33089021 10.1016/j.adro.2019.10.007 PMC7560574

[8] Seung SK , Larson DA , Galvin JM , et al. American College of Radiology (ACR) and American Society for Radiation Oncology (ASTRO) practice guideline for the performance of stereotactic radiosurgery (SRS). Am J Clin Oncol. 2013;36(3):310‐315. 23681017 10.1097/COC.0b013e31826e053d PMC4285440

[9] Moran JM , Dempsey M , Eisbruch A , et al. Safety considerations for IMRT: executive summary. Med Phys. 2011;38(9):5067‐5072. 21978051 10.1118/1.3600524

[10] Hartford AC , Galvin JM , Beyer DC , et al. American College of Radiology (ACR) and American Society for Radiation Oncology (ASTRO) practice guideline for intensity‐modulated radiation therapy (IMRT). Am J Clin Oncol. 2012;35(6):612‐617. 23165357 10.1097/COC.0b013e31826e0515

[11] Miften M , Olch A , Mihailidis D , et al. Tolerance limits and methodologies for IMRT measurement‐based verification QA: recommendations of AAPM Task Group No. 218. Med Phys. 2018;45(4):e53‐e83. 29443390 10.1002/mp.12810

[12] Palmans H , Andreo P , Huq MS , Seuntjens J , Christaki KE , Meghzifene A . Dosimetry of small static fields used in external photon beam radiotherapy: summary of TRS‐483, the IAEA‐AAPM international code of practice for reference and relative dose determination. Med Phys. 2018;45:e1123‐e1145. 30247757 10.1002/mp.13208

[13] Popple RA , Brown MH , Thomas EM , et al. Transition from manual to automated planning and delivery of volumetric modulated arc therapy stereotactic radiosurgery: clinical, dosimetric, and quality assurance results. Pract Radiat Oncol. 2021;11(2):e163‐e171. 33632630 10.1016/j.prro.2020.10.013

[14] Menegotti L , Delana A , Martignano A . Radiochromic film dosimetry with flatbed scanners: a fast and accurate method for dose calibration and uniformity correction with single film exposure. Med Phys. 2008;35(7):3078‐3085. 18697531 10.1118/1.2936334

[15] Micke A , Lewis DF , Yu X . Multichannel film dosimetry with nonuniformity correction. Med Phys. 2011;38(5):2523‐2534. 21776787 10.1118/1.3576105

[16] Palmer AL , Dimitriadis A , Nisbet A , Clark CH . Evaluation of Gafchromic EBT‐XD film, with comparison to EBT3 film, and application in high dose radiotherapy verification. Phys Med Biol. 2015;60(22):8741‐8752. 26512917 10.1088/0031-9155/60/22/8741

[17] Rose MS , Tirpak L , Van Casteren K , et al. Multi‐institution validation of a new high spatial resolution diode array for SRS and SBRT plan pretreatment quality assurance. Med Phys. 2020;47(7):3153‐3164. 32215929 10.1002/mp.14153

[18] Ahmed S , Zhang G , Moros EG , Feygelman V . Comprehensive evaluation of the high‐resolution diode array for SRS dosimetry. J Appl Clin Med Phys. 2019;20(10):13‐23. 10.1002/acm2.12696 PMC6806480 31478343

[19] Low DA , Harms WB , Mutic S , Purdy JA . A technique for the quantitative evaluation of dose distributions. Med Phys. 1998;25(5):656‐661. 9608475 10.1118/1.598248

[20] Ju T , Simpson T , Deasy JO , Low DA . Geometric interpretation of the gamma dose distribution comparison technique: interpolation‐free calculation. Med Phys. 2008;35(3):879‐887. 18404924 10.1118/1.2836952

[21] Xia Y , Adamson J , Zlateva Y , Giles W . Application of TG‐218 action limits to SRS and SBRT pre‐treatment patient specific QA. J Radiosurg SBRT. 2020;7(2):135‐147. 33282467 PMC7717087

[22] Snyder JD , Sullivan RJ , Wu X , Covington EL , Popple RA . Use of a plastic scintillator detector for patient‐specific quality assurance of VMAT SRS. J Appl Clin Med Phys. 2019;20(9):143‐148. 31538717 10.1002/acm2.12705 PMC6753731

[23] Covington EL , Snyder JD , Wu X , Cardan RA , Popple RA . Assessing the feasibility of single target radiosurgery quality assurance with portal dosimetry. J Appl Clin Med Phys. 2019;20(5):135‐140. 10.1002/acm2.12578 PMC6522988 30933414

