# Supplementary material for: Evaluation of a two‐dimensional diode array for patient‐specific quality assurance of HyperArc
Source: J Appl Clin Med Phys. 2021 Nov 1;22(12):203–10. doi: 10.1002/acm2.13438 (PMC8664143; doi:10.1002/acm2.13438)

Table of Contents

Measurement 1. Plan 1, single target, equivalent diameter 15.9 mm ... 1
Measurement 2. Plan 2, single target, equivalent diameter 28.2 mm ... 2
Measurement 3. Plan 3, single target, equivalent diameter 20.1 mm ... 3
Measurement 4. Plan 4, 3 targets, equivalent diameter 5.8 mm ... 4
Measurement 5. Plan 4, 3 targets, equivalent diameter 19.9 mm ... 5
Measurement 6. Plan 5, 4 targets, equivalent diameter 13.3 mm ... 6
Measurement 7. Plan 5, 4 targets, equivalent diameter 23.4 mm ... 7
Measurement 8. Plan 6, 17 targets, equivalent diameter 2.8 mm ... 8
Measurement 9. Plan 6, 17 targets, equivalent diameter 5.8 mm ... 9
Measurement 10. Plan 7, 9 targets, equivalent diameter 2.8 mm ... 10
Measurement 11. Plan 7, 9 targets, equivalent diameter 5.8 mm ... 11
Measurement 12. Plan 8, 9 targets, equivalent diameter 5.3 mm ... 12
Measurement 13. Plan 8, 9 targets, equivalent diameter 20.1 mm ... 13
Measurement 14. Plan 9, single target, equivalent diameter 10.2 mm ... 14
Measurement 15. Plan 10, single target, equivalent diameter 10.4 mm ... 15
Measurement 16. Plan 11, 25 targets, equivalent diameter 3.5 mm ... 16
Measurement 17. Plan 11, 25 targets, equivalent diameter 18.2 mm ... 17
Measurement 18. Plan 12, single target, equivalent diameter 26.1 mm ... 18
Measurement 19. Plan 13, single target, equivalent diameter 6.9 mm ... 19
Measurement 20. Plan 14, 4 targets, equivalent diameter 6.8 mm ... 20
Measurement 21. Plan 14, 4 targets, equivalent diameter 16.2 mm ... 21
Measurement 22. Plan 15, 2 targets, equivalent diameter 3.6 mm ... 22
Measurement 23. Plan 15, 2 targets, equivalent diameter 7.0 mm ... 23
Measurement 24. Plan 16, 7 targets, equivalent diameter 4.1 mm ... 24
Measurement 25. Plan 16, 7 targets, equivalent diameter 17.5 mm ... 25
Measurement 26. Plan 17, 7 targets, equivalent diameter 3.9 mm ... 26
Measurement 27. Plan 17, 7 targets, equivalent diameter 11.1 mm ... 27
Measurement 28. Plan 18, single target, equivalent diameter 19.0 mm ... 28
Measurement 29. Plan 19, 9 targets, equivalent diameter 4.2 mm ... 29
Measurement 30. Plan 19, 9 targets, equivalent diameter 17.1 mm ... 30
Measurement 31. Plan 20, 4 targets, equivalent diameter 5.7 mm ... 31
Measurement 32. Plan 20, 4 targets, equivalent diameter 14.7 mm ... 32
Measurement 33. Plan 21, single target, equivalent diameter 10.5 mm ... 33
Measurement 34. Plan 22, 6 targets, equivalent diameter 4.0 mm ... 34
Measurement 35. Plan 22, 6 targets, equivalent diameter 8.7 mm ... 35
Measurement 36. Plan 23, 9 targets, equivalent diameter 3.0 mm ... 36
Measurement 37. Plan 23, 9 targets, equivalent diameter 6.5 mm ... 37
Measurement 38. Plan 24, single target, equivalent diameter 6.0 mm ... 38
Measurement 39. Plan 25, single target, equivalent diameter 3.0 mm ... 39
Measurement 40. Plan 26, 5 targets, equivalent diameter 3.0 mm ... 40
Measurement 41. Plan 26, 5 targets, equivalent diameter 11.8 mm ... 41
Measurement 42. Plan 27, single target, equivalent diameter 16.2 mm ... 42
Measurement 43. Plan 28, single target, equivalent diameter 8.6 mm ... 43

Measurement 44. Plan 29, single target, equivalent diameter 23.2 mm 44
Measurement 45. Plan 30, 4 targets, equivalent diameter 4.9 mm 45
Measurement 46. Plan 30, 4 targets, equivalent diameter 19.9 mm 46
Measurement 47. Plan 31, 7 targets, equivalent diameter 3.2 mm 47
Measurement 48. Plan 31, 7 targets, equivalent diameter 20.4 mm 48
Measurement 49. Plan 32, 10 targets, equivalent diameter 2.4 mm 49
Measurement 50. Plan 32, 10 targets, equivalent diameter 3.8 mm 50
Measurement 51. Plan 33, 8 targets, equivalent diameter 8.3 mm 51
Measurement 52. Plan 33, 8 targets, equivalent diameter 15.7 mm 52
Measurement 53. Plan 34, 2 targets, equivalent diameter 10.8 mm 53
Measurement 54. Plan 34, 2 targets, equivalent diameter 15.7 mm 54
Measurement 55. Plan 35, single target, equivalent diameter 17.0 mm 55
Measurement 56. Plan 36, single target, equivalent diameter 20.0 mm 56
Measurement 57. Plan 37, 2 targets, equivalent diameter 3.8 mm 57
Measurement 58. Plan 37, 2 targets, equivalent diameter 6.6 mm 58
Measurement 59. Plan 38, 2 targets, equivalent diameter 4.6 mm 59
Measurement 60. Plan 38, 2 targets, equivalent diameter 5.9 mm 60
Measurement 61. Plan 39, single target, equivalent diameter 5.9 mm 61
Measurement 62. Plan 40, 4 targets, equivalent diameter 8.3 mm 62
Measurement 63. Plan 40, 4 targets, equivalent diameter 11.8 mm 63
Measurement 64. Plan 41, 2 targets, equivalent diameter 3.3 mm 64
Measurement 65. Plan 41, 2 targets, equivalent diameter 26.7 mm 65
Measurement 66. Plan 42, 2 targets, equivalent diameter 3.4 mm 66
Measurement 67. Plan 42, 2 targets, equivalent diameter 10.8 mm 67
Measurement 68. Plan 43, 2 targets, equivalent diameter 2.4 mm 68
Measurement 69. Plan 43, 2 targets, equivalent diameter 5.5 mm 69
Measurement 70. Plan 44, 2 targets, equivalent diameter 5.6 mm 70
Measurement 71. Plan 44, 2 targets, equivalent diameter 9.2 mm 71
Measurement 72. Plan 45, single target, equivalent diameter 13.1 mm 72
Measurement 73. Plan 46, 2 targets, equivalent diameter 4.2 mm 73
Measurement 74. Plan 46, 2 targets, equivalent diameter 8.8 mm 74
Measurement 75. Plan 47, single target, equivalent diameter 35.8 mm 75
Measurement 76. Plan 48, 6 targets, equivalent diameter 2.7 mm 76
Measurement 77. Plan 48, 6 targets, equivalent diameter 6.6 mm 77
Measurement 78. Plan 49, 7 targets, equivalent diameter 3.5 mm 78
Measurement 79. Plan 49, 7 targets, equivalent diameter 44.7 mm 79
Measurement 80. Plan 50, single target, equivalent diameter 13.6 mm 80
Measurement 81. Plan 51, single target, equivalent diameter 15.2 mm 81
Measurement 82. Plan 52, single target, equivalent diameter 30.9 mm 82
Measurement 83. Plan 53, single target, equivalent diameter 19.5 mm 83
Measurement 84. Plan 54, single target, equivalent diameter 31.7 mm 84
Measurement 85. Plan 55, single target, equivalent diameter 12.2 mm 85
Measurement 86. Plan 56, single target, equivalent diameter 4.8 mm 86
Measurement 87. Plan 57, single target, equivalent diameter 25.4 mm 87
Measurement 88. Plan 58, single target, equivalent diameter 21.3 mm 88
Measurement 89. Plan 59, single target, equivalent diameter 7.7 mm 89

Measurement 90. Plan 60, single target, equivalent diameter 25.1 mm 90

Measurement 1. Plan 1, single target, equivalent diameter 15.9 mm

SRS MapCHECK fraction passing gamma 3%/1 mm = 99.5%

Radichromic film fraction passing gamma 3%/1 mm = 96.2%

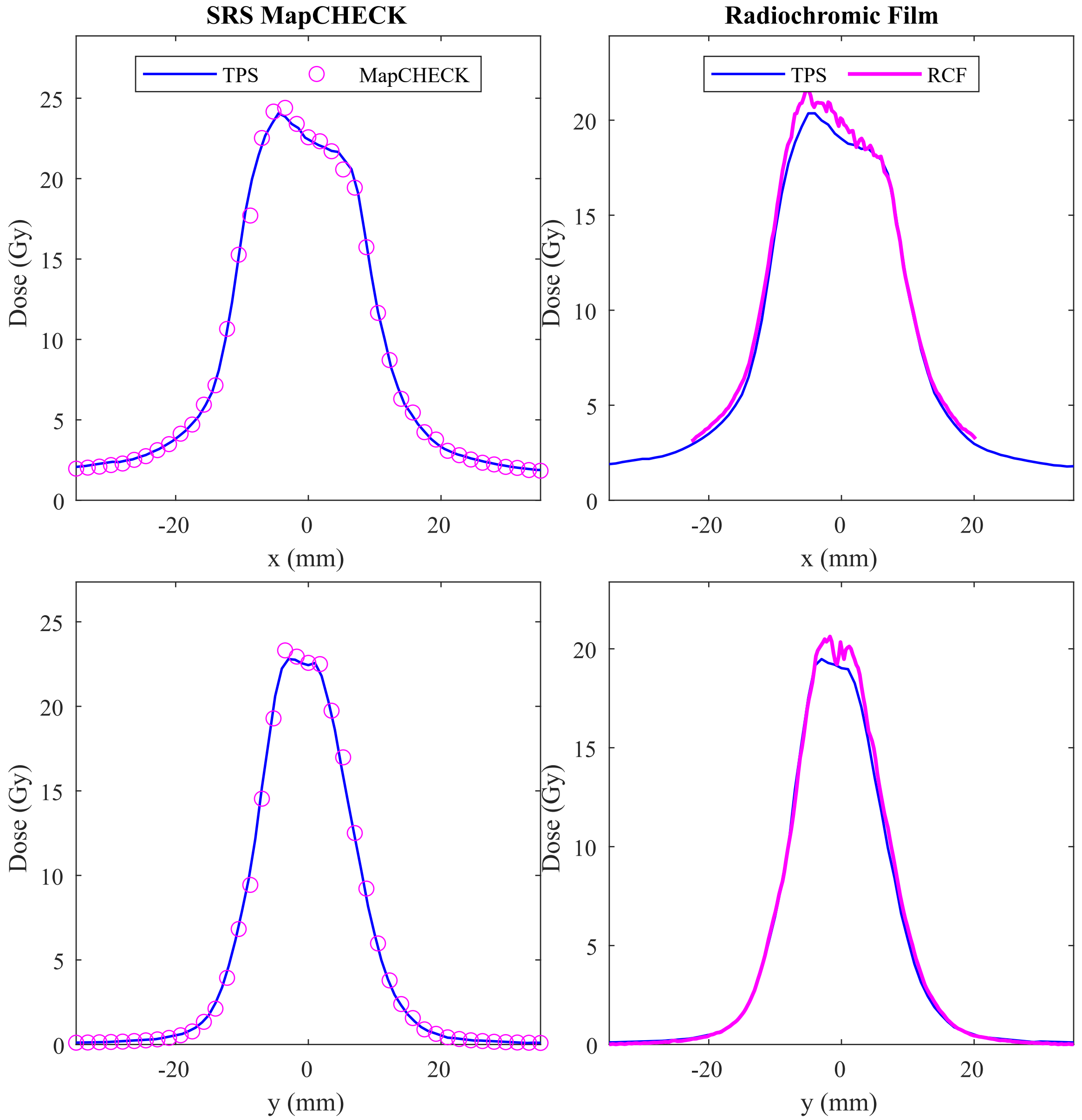

Measurement 2. Plan 2, single target, equivalent diameter 28.2 mm

SRS MapCHECK fraction passing gamma 3%/1 mm = 100.0%

Radichromic film fraction passing gamma 3%/1 mm = 86.5%

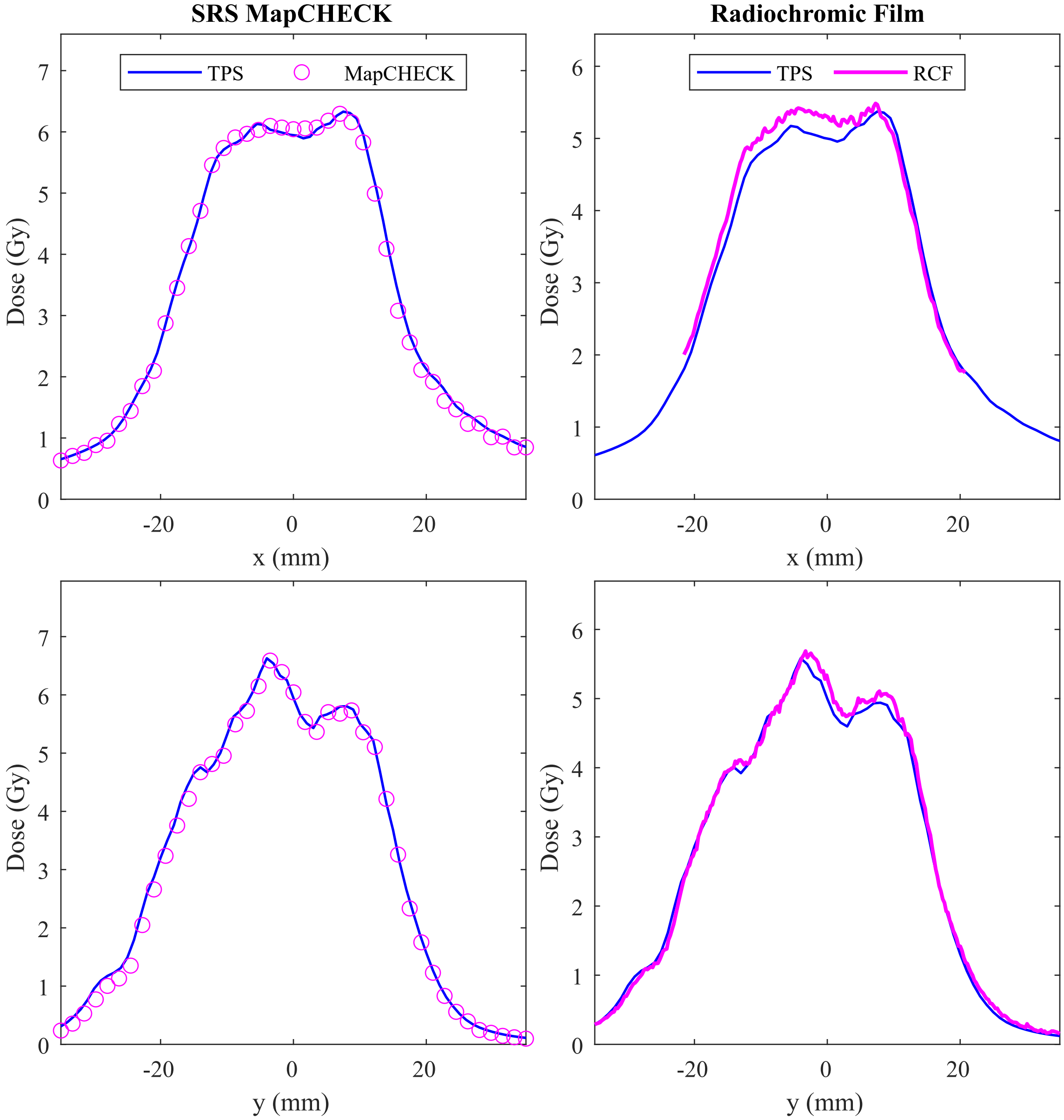

Measurement 3. Plan 3, single target, equivalent diameter 20.1 mm

SRS MapCHECK fraction passing gamma 3%/1 mm = 100.0%

Radichromic film fraction passing gamma 3%/1 mm = 98.4%

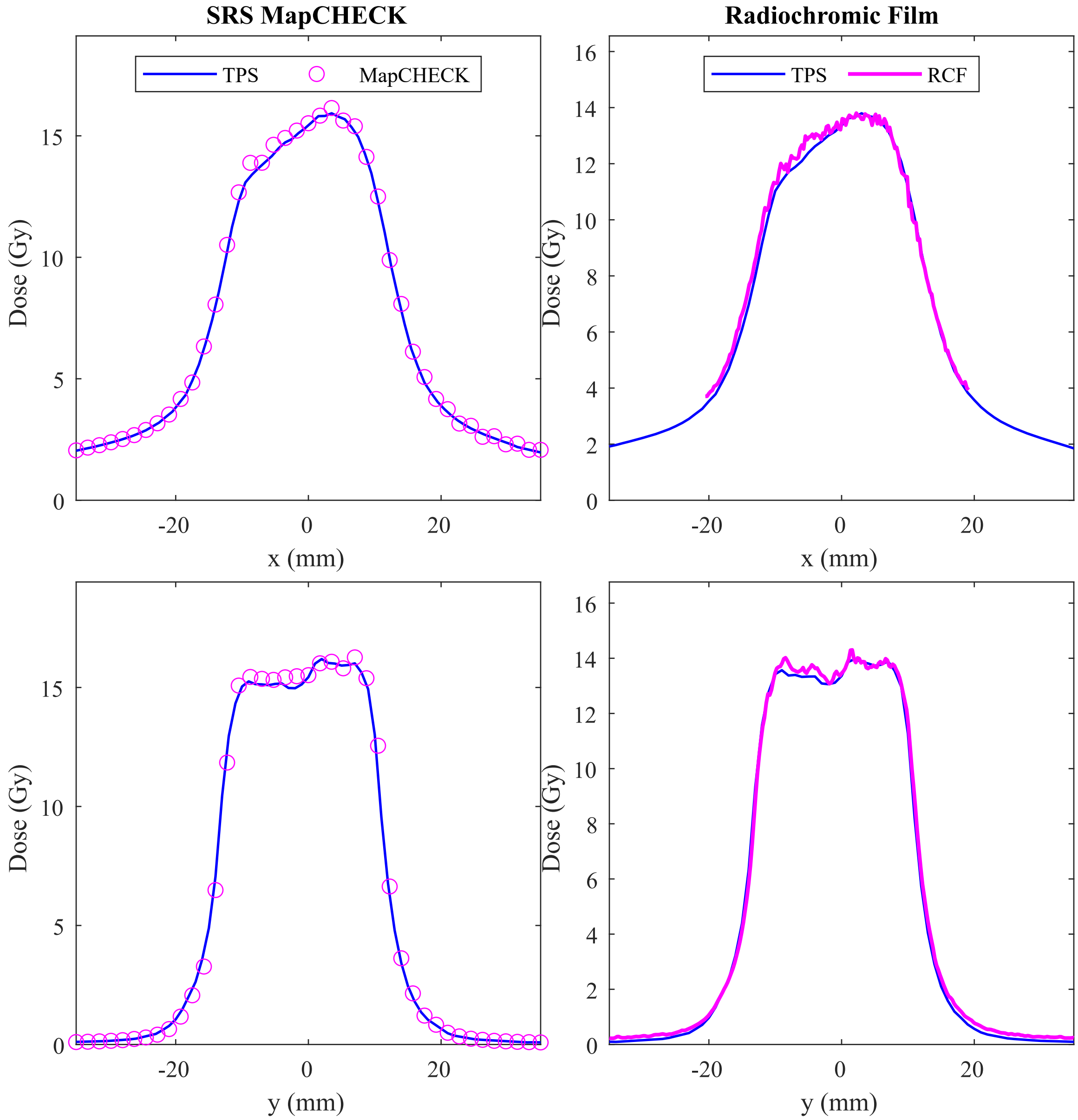

Measurement 4. Plan 4, 3 targets, equivalent diameter 5.8 mm

SRS MapCHECK fraction passing gamma 3%/1 mm = 100.0%

Radichromic film fraction passing gamma 3%/1 mm = 99.4%

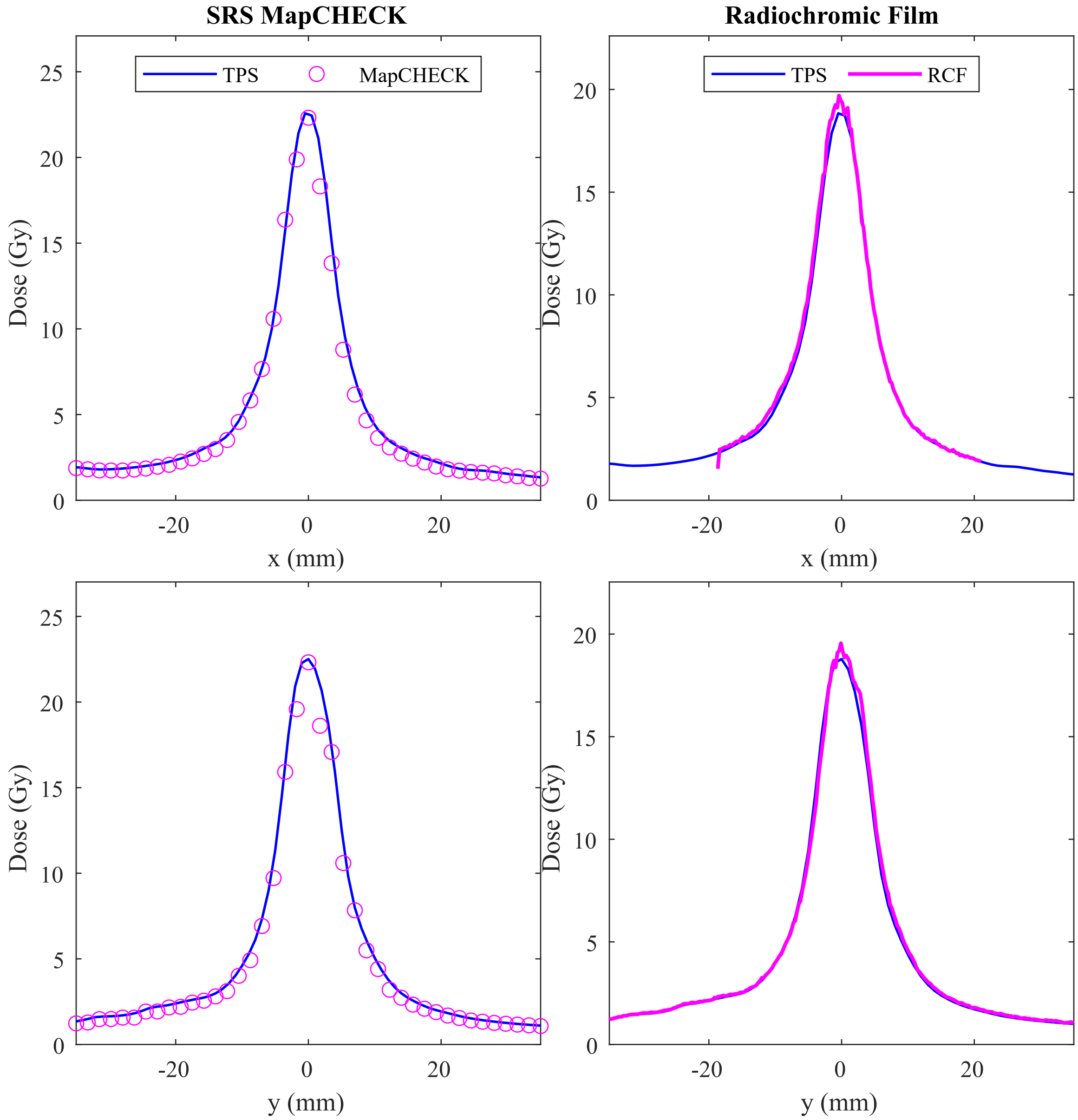

Measurement 5. Plan 4, 3 targets, equivalent diameter 19.9 mm

SRS MapCHECK fraction passing gamma 3%/1 mm = 100.0%

Radichromic film fraction passing gamma 3%/1 mm = 99.5%

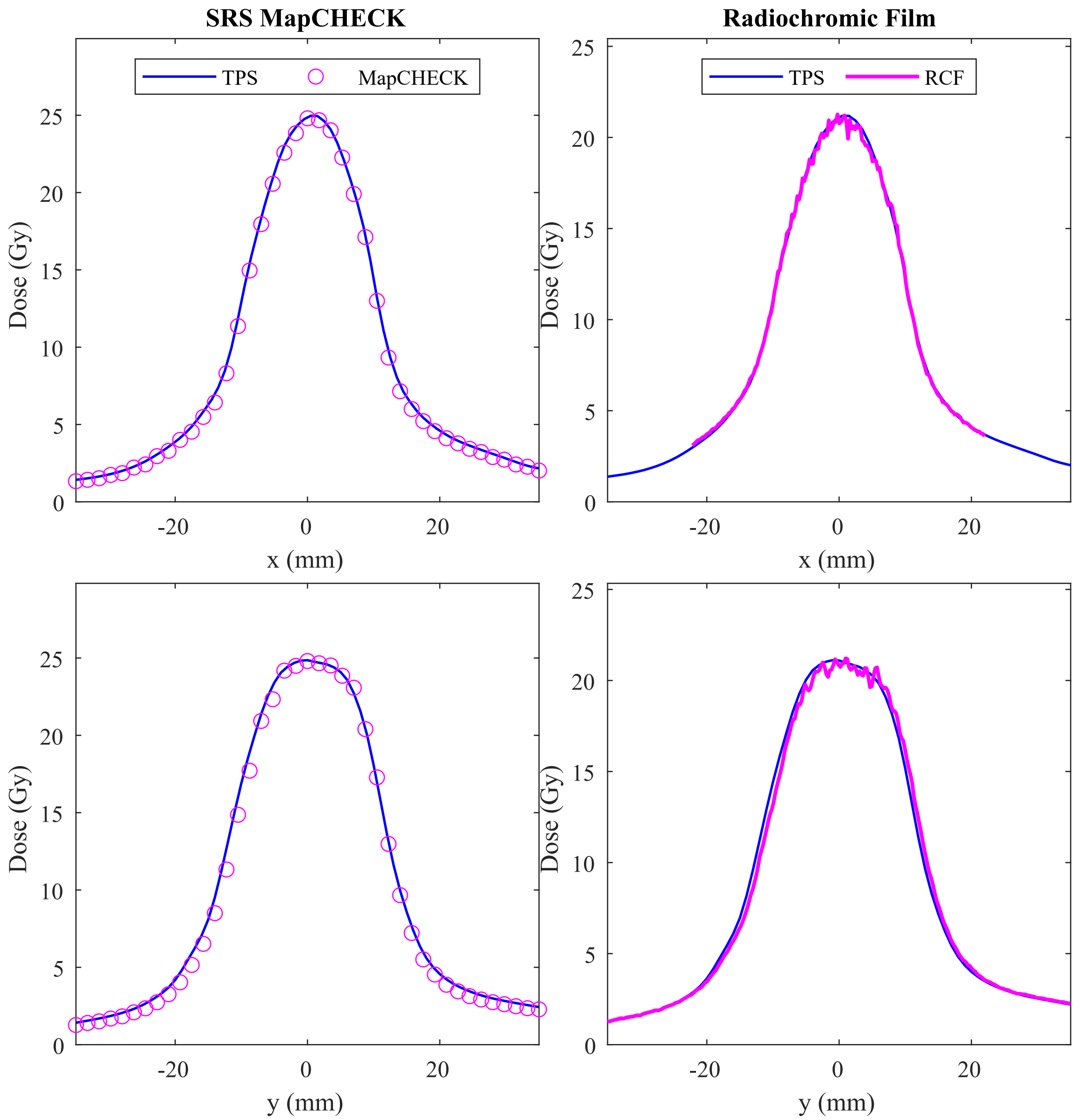

Measurement 6. Plan 5, 4 targets, equivalent diameter 13.3 mm

SRS MapCHECK fraction passing gamma 3%/1 mm = 100.0%

Radichromic film fraction passing gamma 3%/1 mm = 95.2%

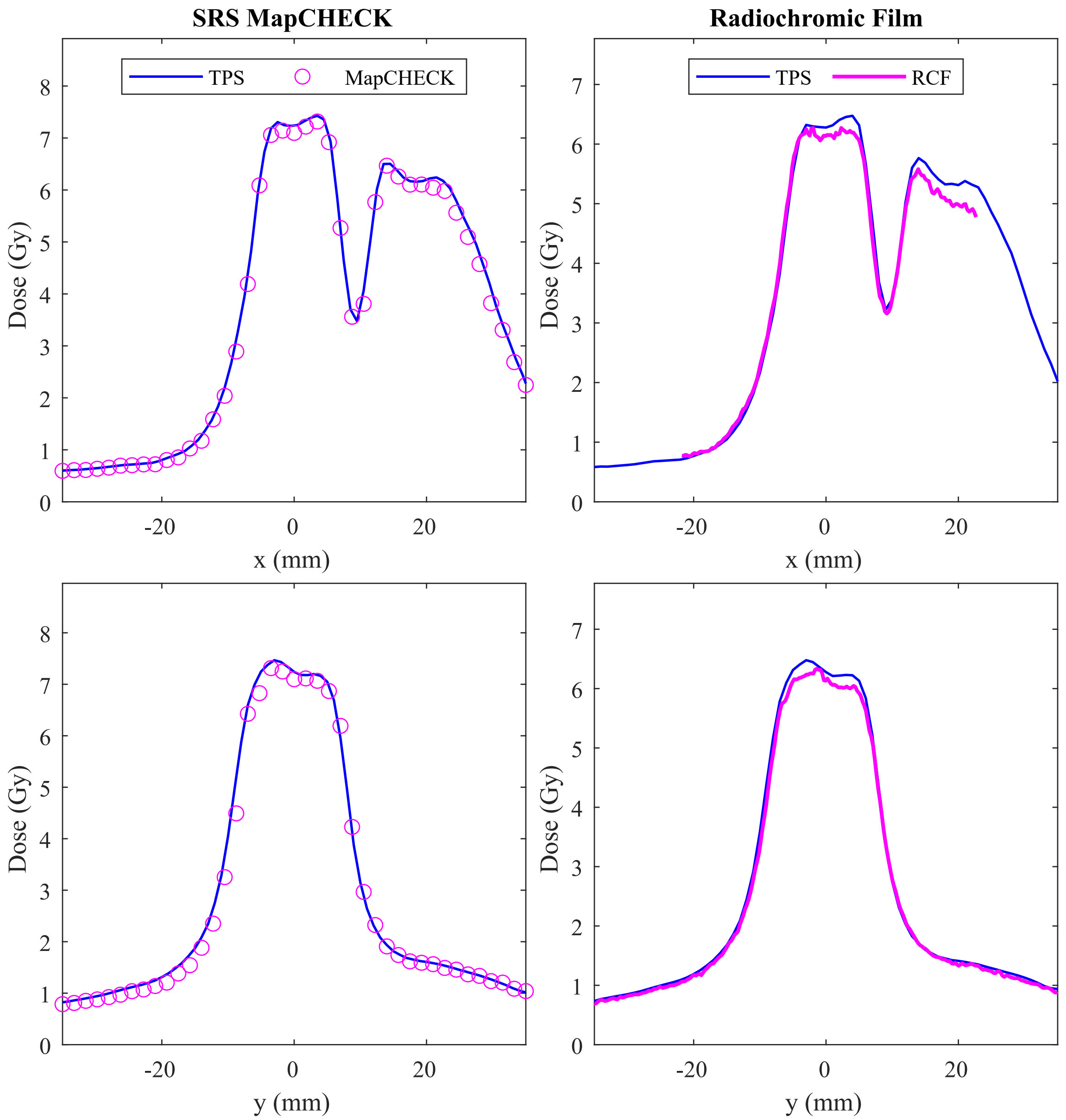

Measurement 7. Plan 5, 4 targets, equivalent diameter 23.4 mm

SRS MapCHECK fraction passing gamma 3%/1 mm = 97.9%

Radichromic film fraction passing gamma 3%/1 mm = 95.2%

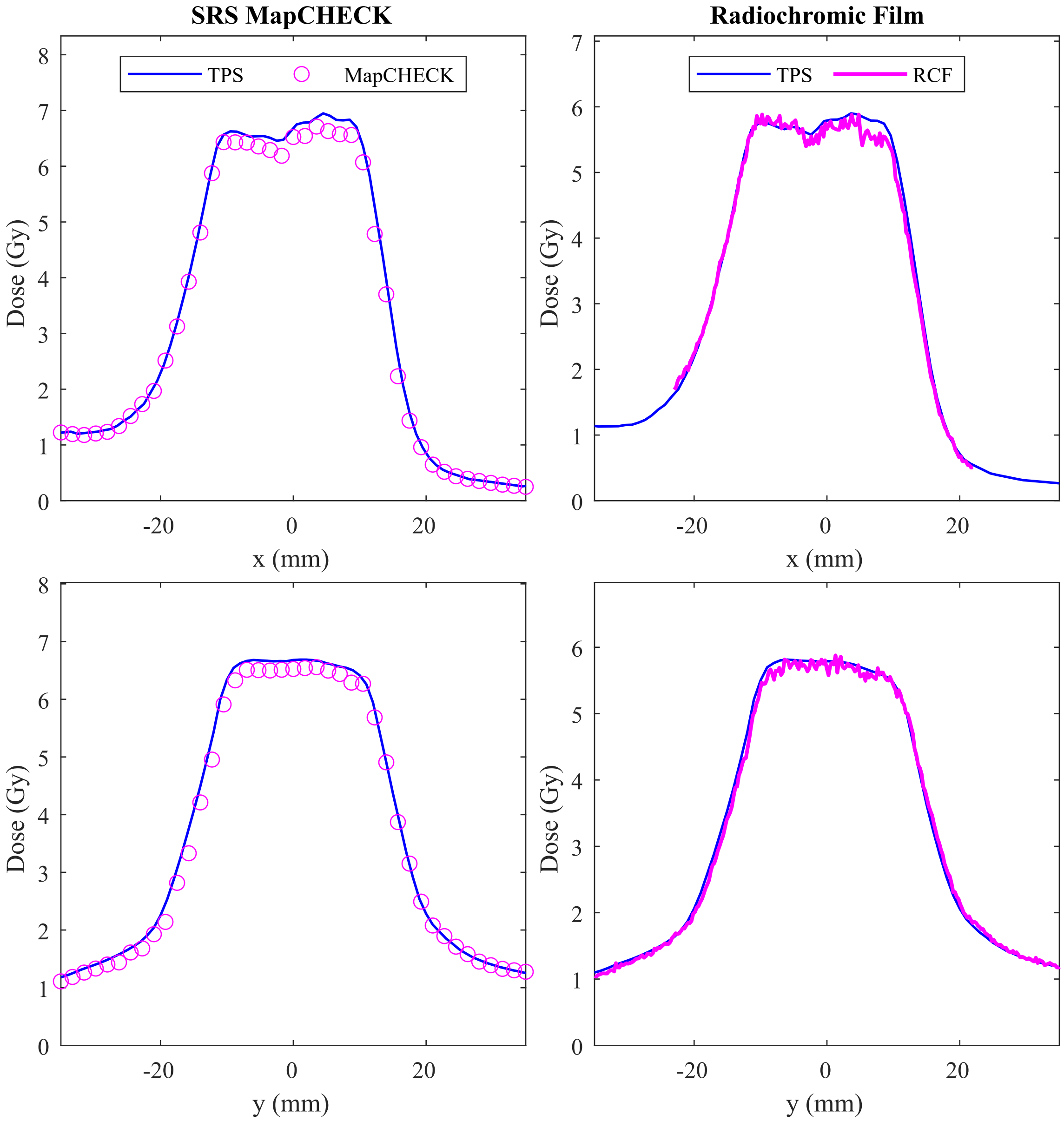

Measurement 8. Plan 6, 17 targets, equivalent diameter 2.8 mm

SRS MapCHECK fraction passing gamma 3%/1 mm = 100.0%

Radichromic film fraction passing gamma 3%/1 mm = 100.0%

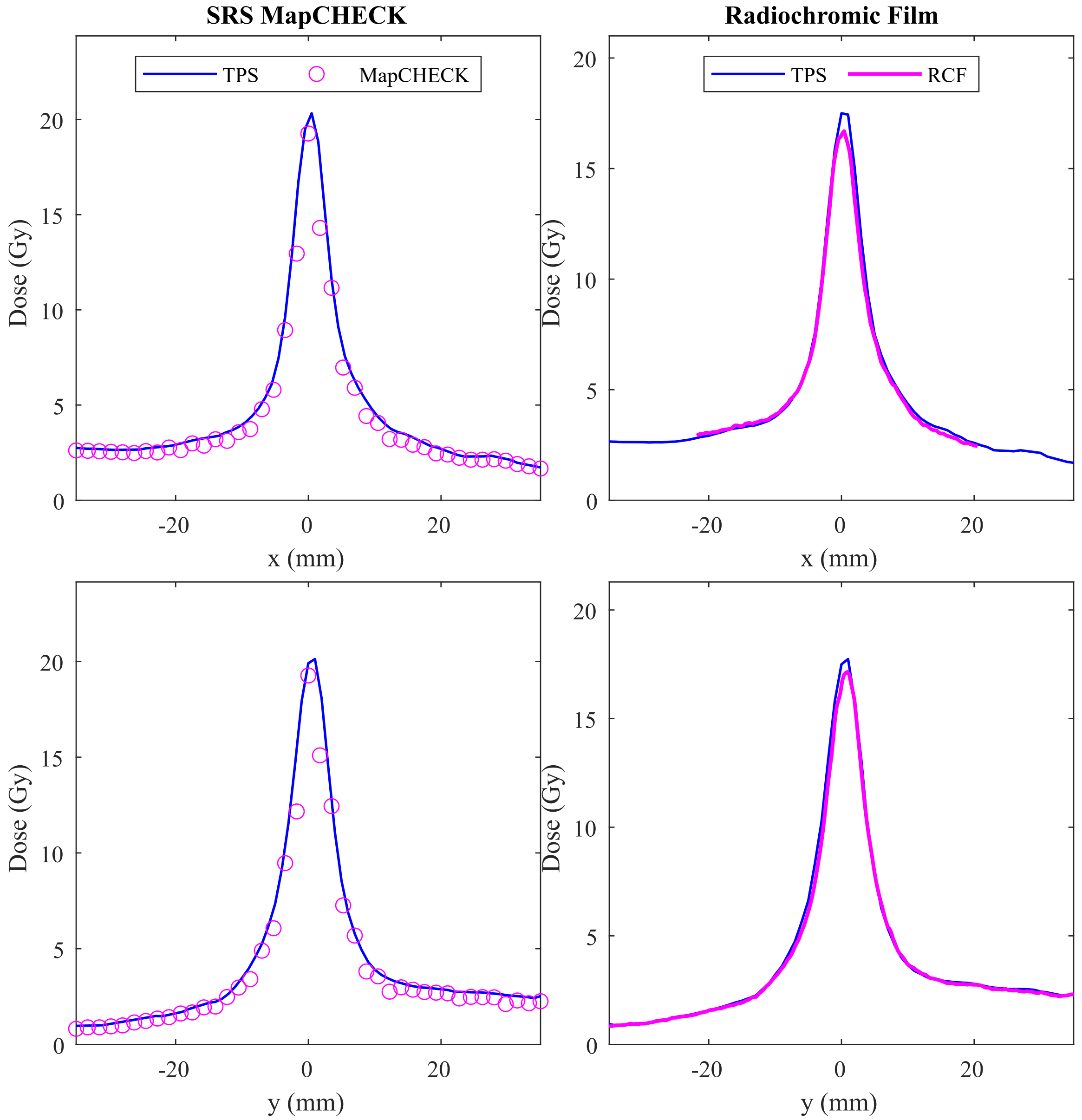

Measurement 9. Plan 6, 17 targets, equivalent diameter 5.8 mm

SRS MapCHECK fraction passing gamma 3%/1 mm = 99.8%

Radichromic film fraction passing gamma 3%/1 mm = 100.0%

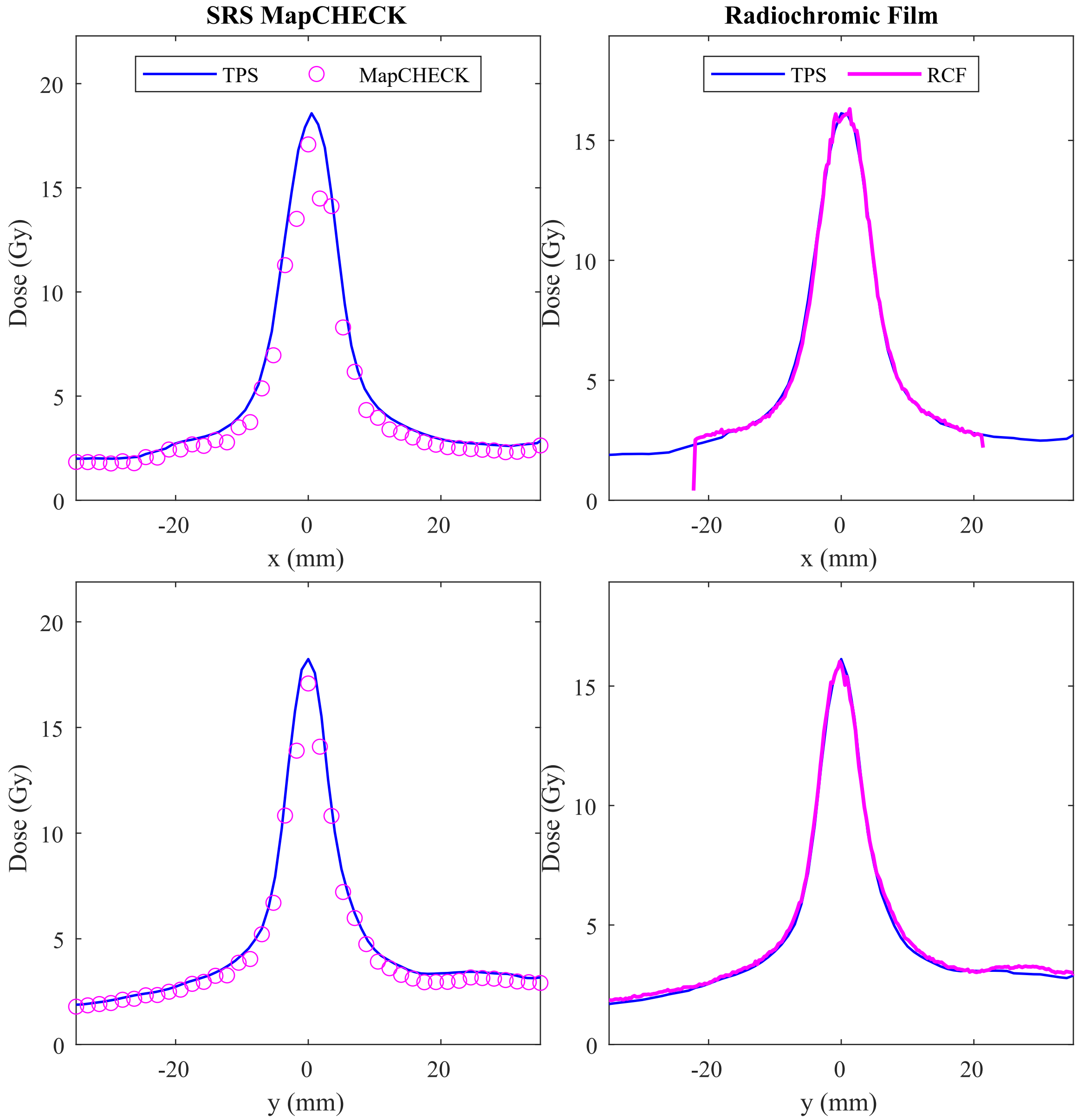

Measurement 10. Plan 7, 9 targets, equivalent diameter 2.8 mm

SRS MapCHECK fraction passing gamma 3%/1 mm = 93.4%

Radichromic film fraction passing gamma 3%/1 mm = 100.0%

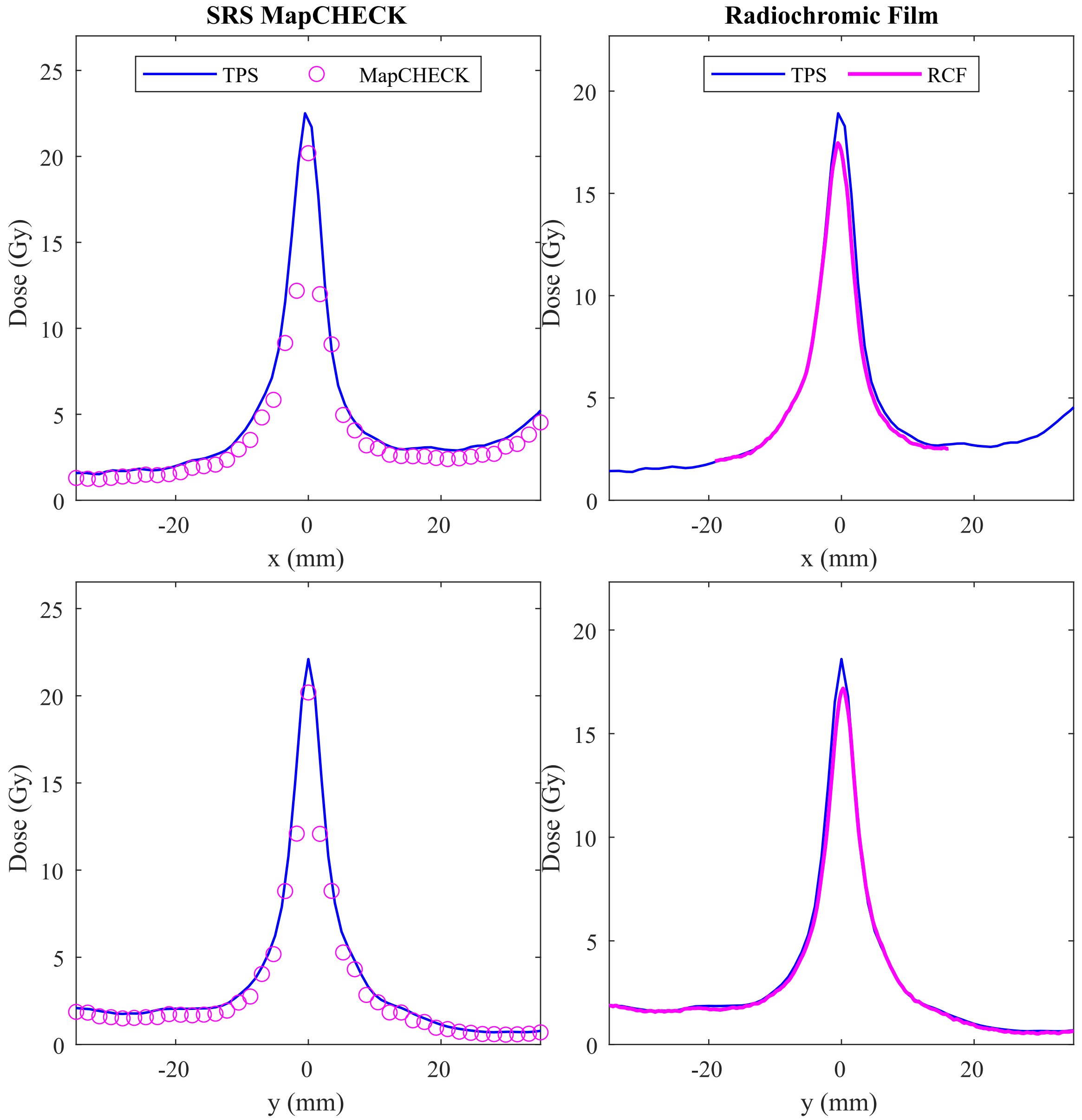

Measurement 11. Plan 7, 9 targets, equivalent diameter 5.8 mm

SRS MapCHECK fraction passing gamma 3%/1 mm = 91.4%

Radichromic film fraction passing gamma 3%/1 mm = 99.8%

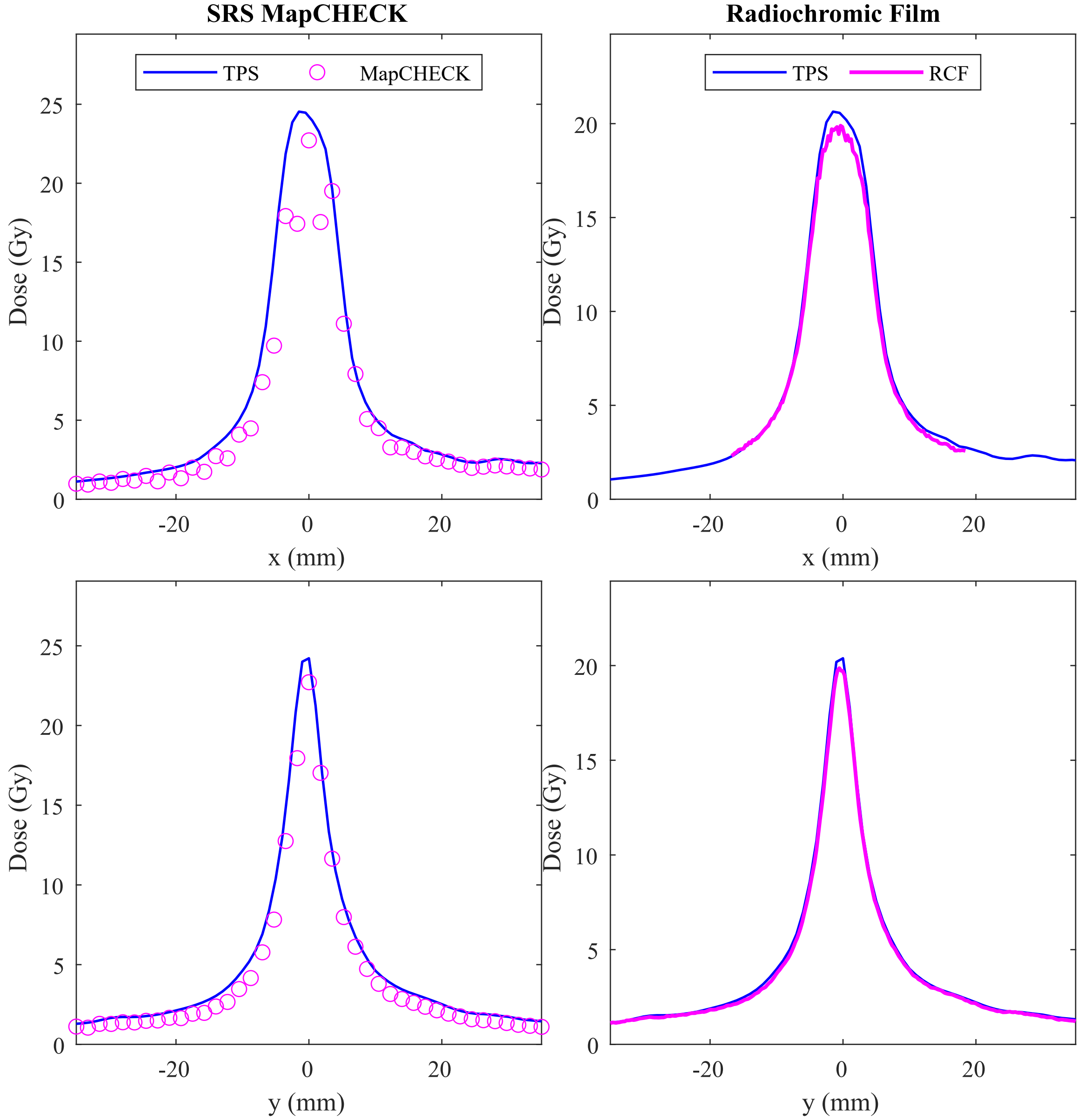

Measurement 12. Plan 8, 9 targets, equivalent diameter 5.3 mm

SRS MapCHECK fraction passing gamma 3%/1 mm = 100.0%

Radichromic film fraction passing gamma 3%/1 mm = 100.0%

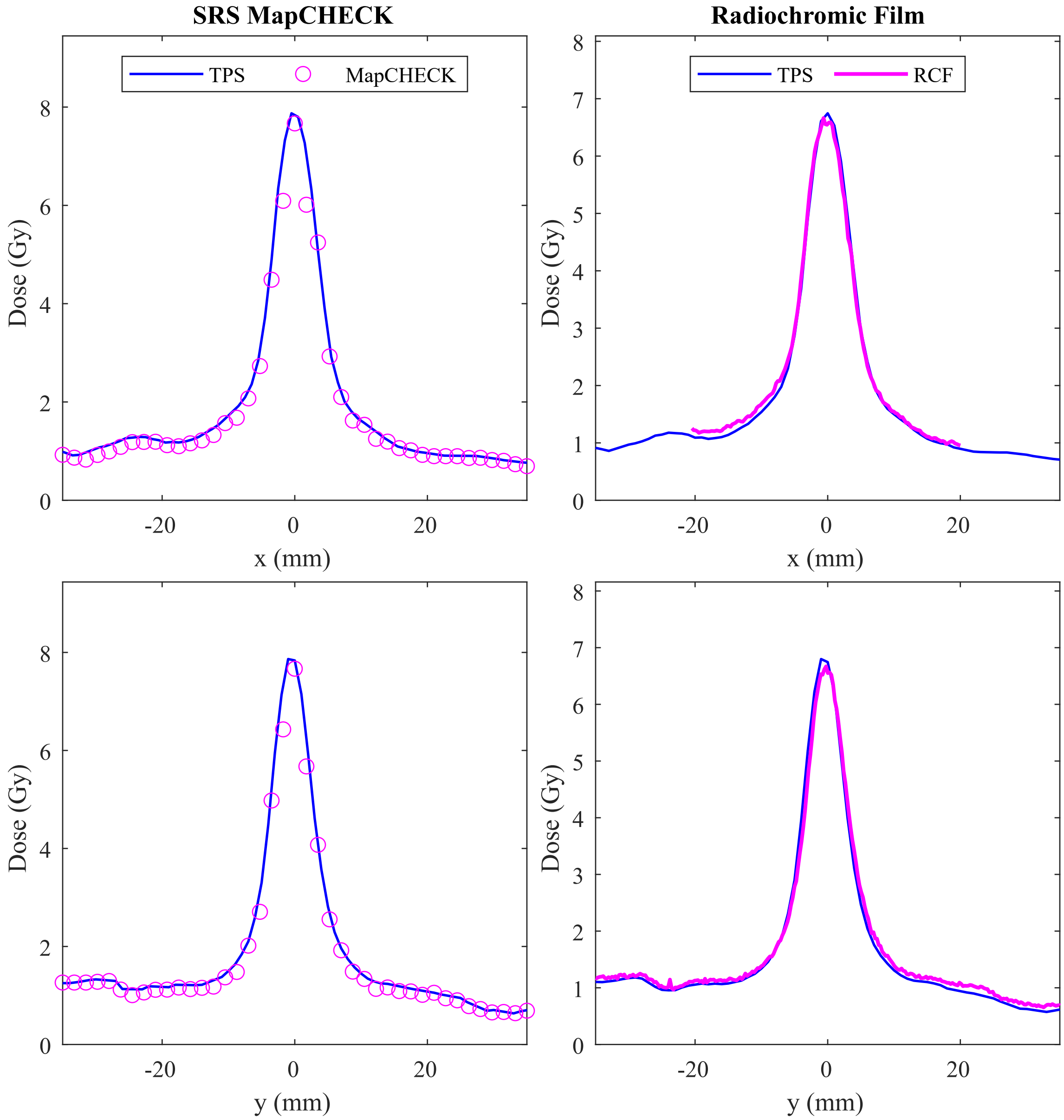

Measurement 13. Plan 8, 9 targets, equivalent diameter 20.1 mm

SRS MapCHECK fraction passing gamma 3%/1 mm = 99.2%

Radichromic film fraction passing gamma 3%/1 mm = 98.5%

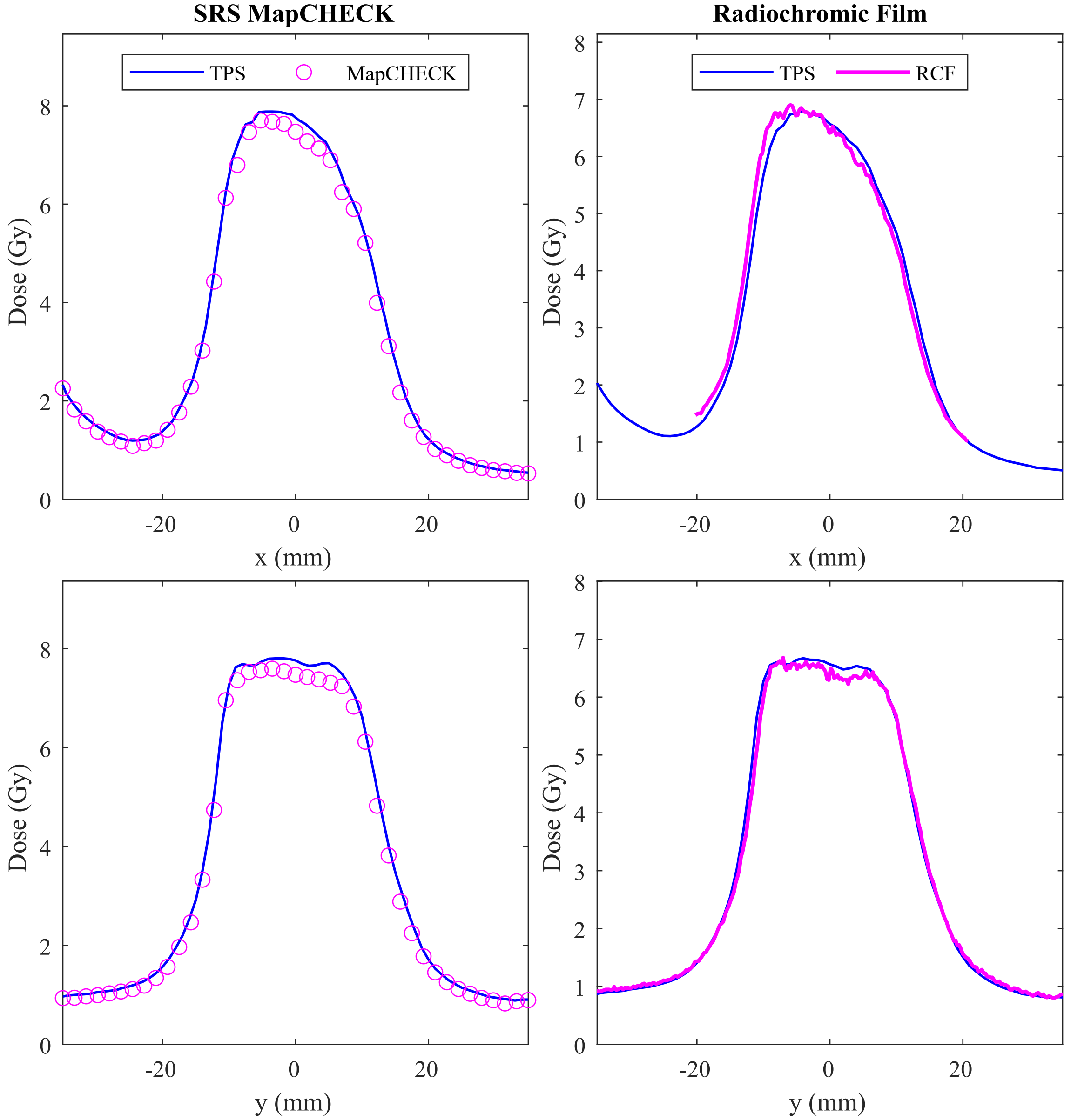

Measurement 14. Plan 9, single target, equivalent diameter 10.2 mm

SRS MapCHECK fraction passing gamma 3%/1 mm = 99.3%

Radichromic film fraction passing gamma 3%/1 mm = 99.6%

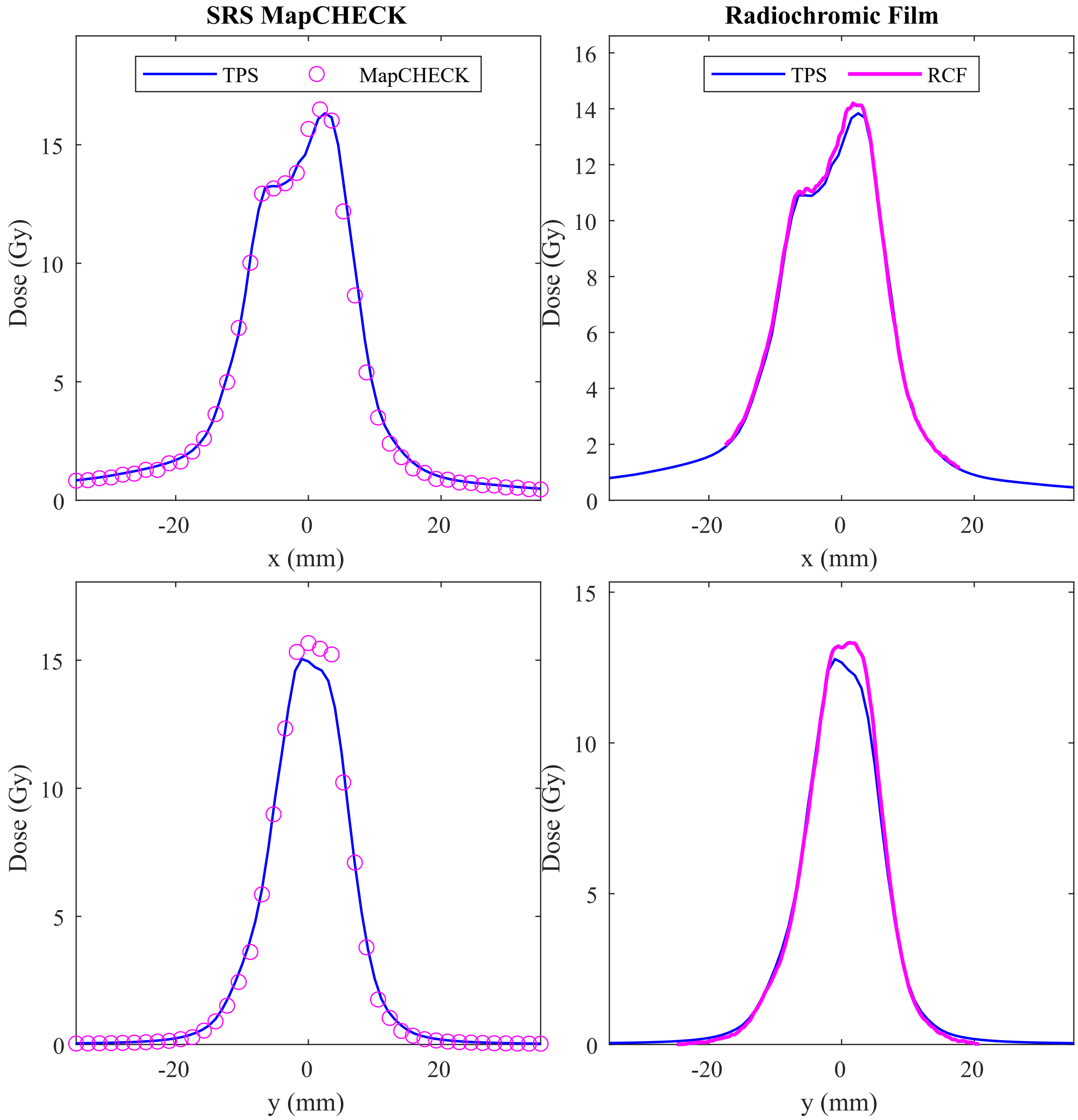

Measurement 15. Plan 10, single target, equivalent diameter 10.4 mm

SRS MapCHECK fraction passing gamma 3%/1 mm = 100.0%

Radichromic film fraction passing gamma 3%/1 mm = 88.1%

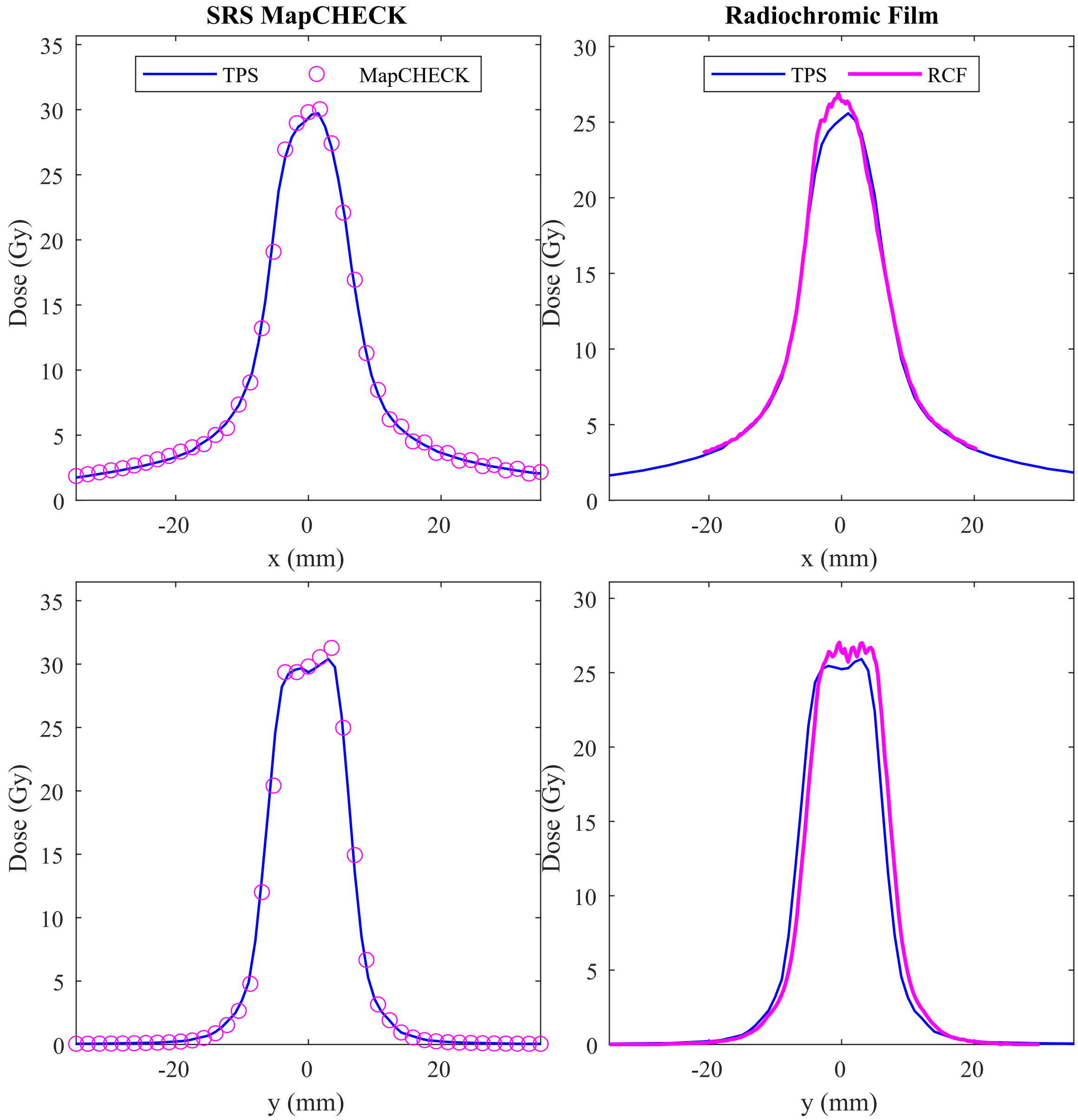

Measurement 16. Plan 11, 25 targets, equivalent diameter 3.5 mm

SRS MapCHECK fraction passing gamma 3%/1 mm = 98.9%

Radichromic film fraction passing gamma 3%/1 mm = 97.1%

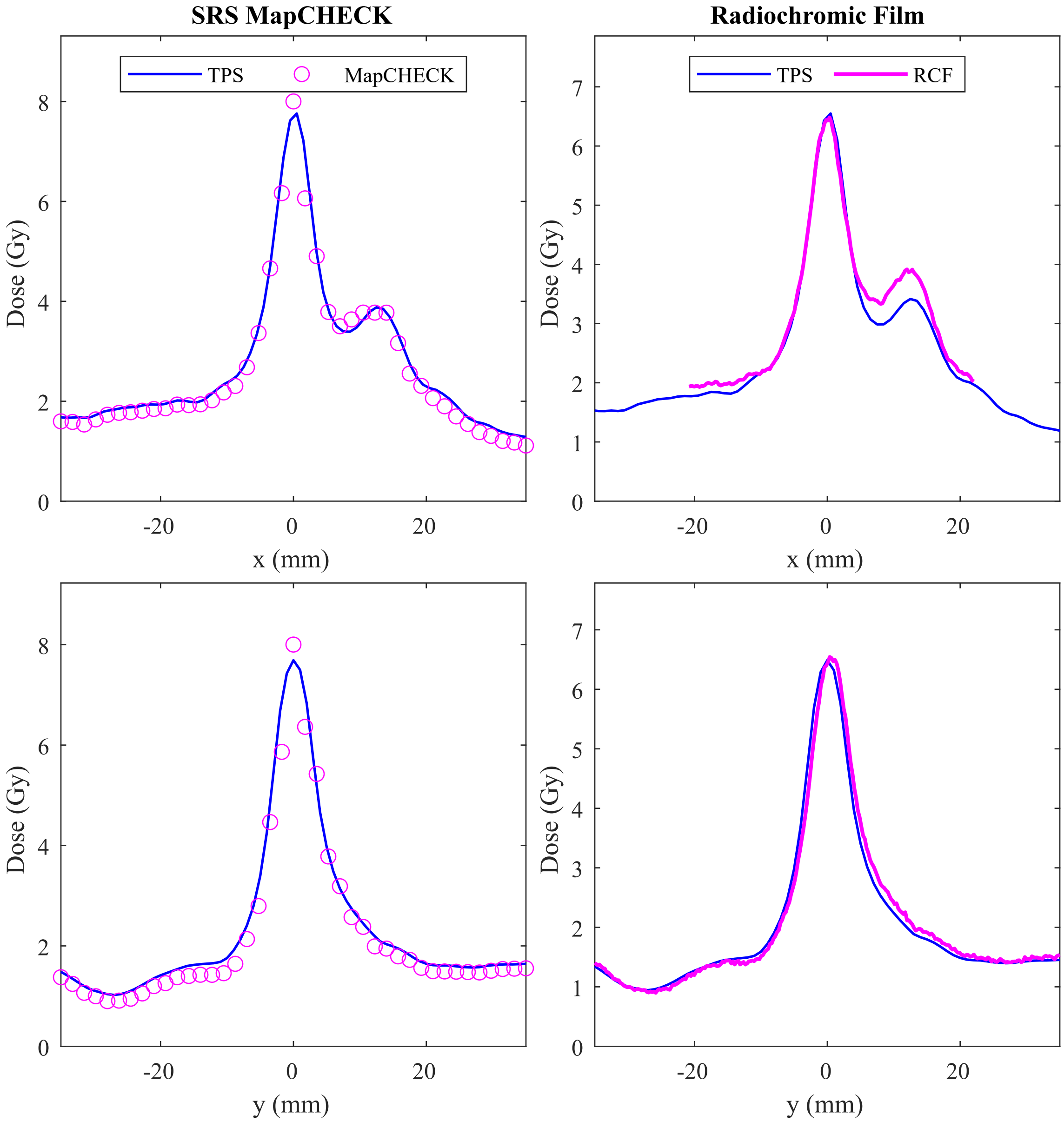

Measurement 17. Plan 11, 25 targets, equivalent diameter 18.2 mm

SRS MapCHECK fraction passing gamma 3%/1 mm = 100.0%

Radichromic film fraction passing gamma 3%/1 mm = 99.9%

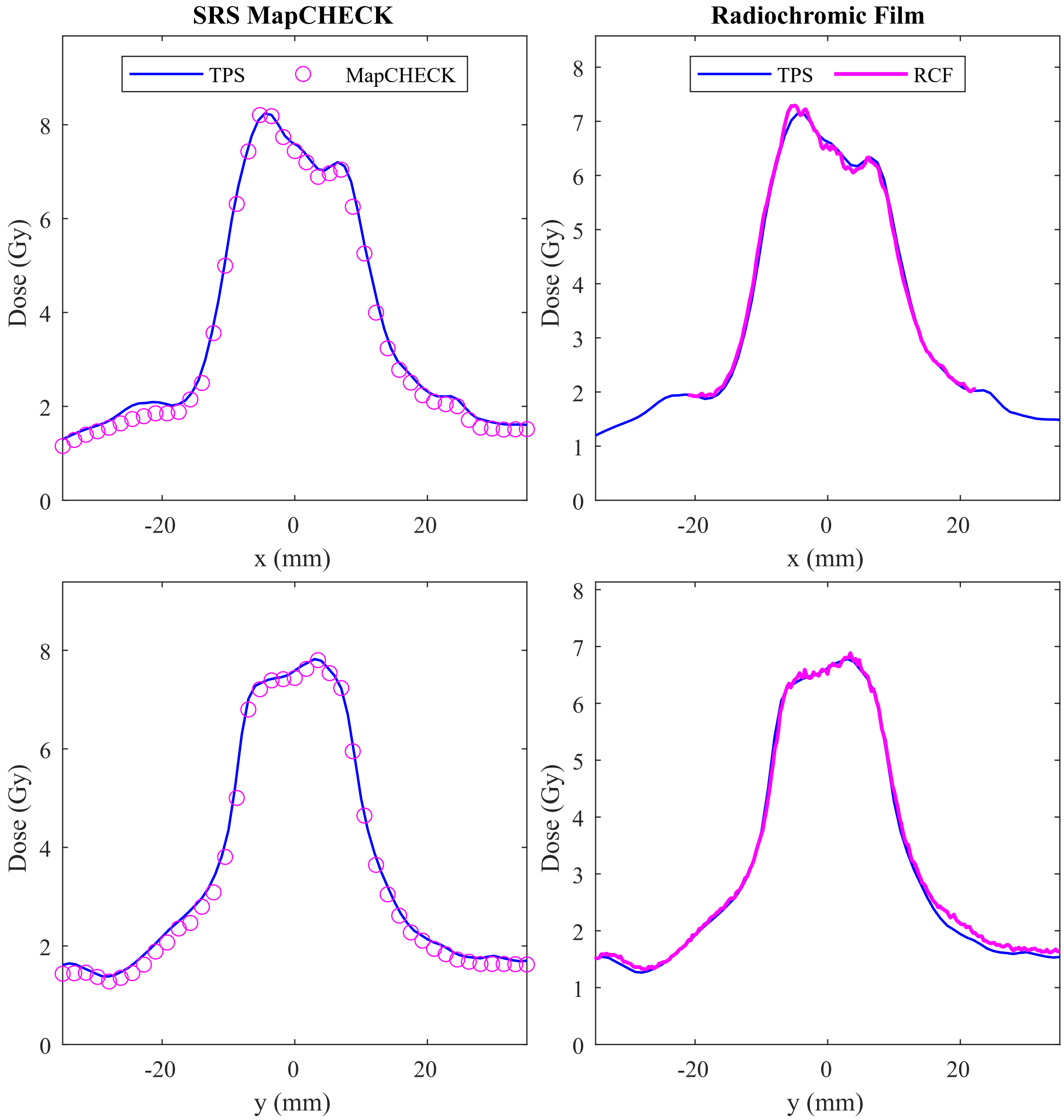

Measurement 18. Plan 12, single target, equivalent diameter 26.1 mm

SRS MapCHECK fraction passing gamma 3%/1 mm = 100.0%

Radichromic film fraction passing gamma 3%/1 mm = 99.3%

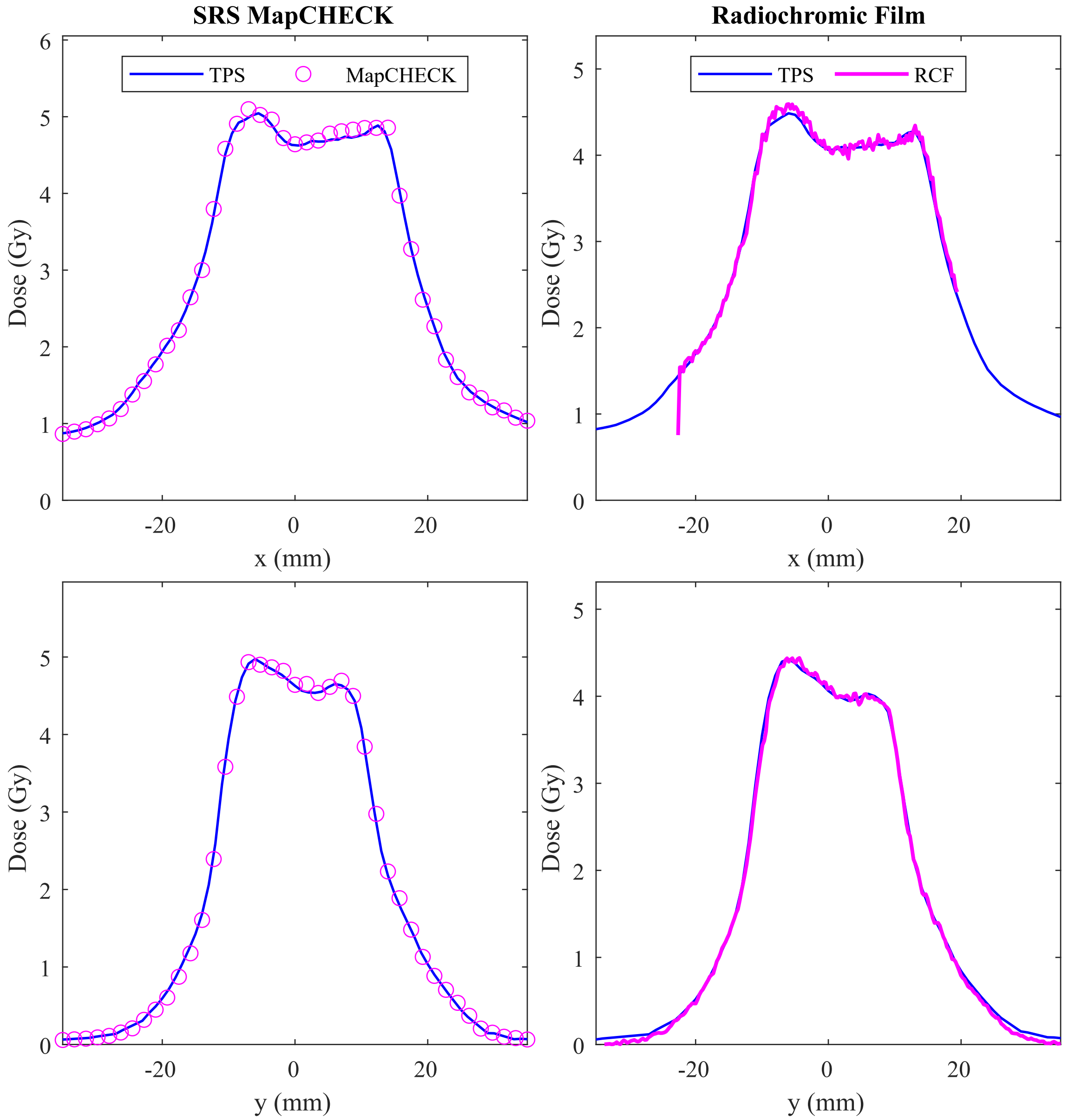

Measurement 19. Plan 13, single target, equivalent diameter 6.9 mm

SRS MapCHECK fraction passing gamma 3%/1 mm = 97.0%

Radichromic film fraction passing gamma 3%/1 mm = 99.4%

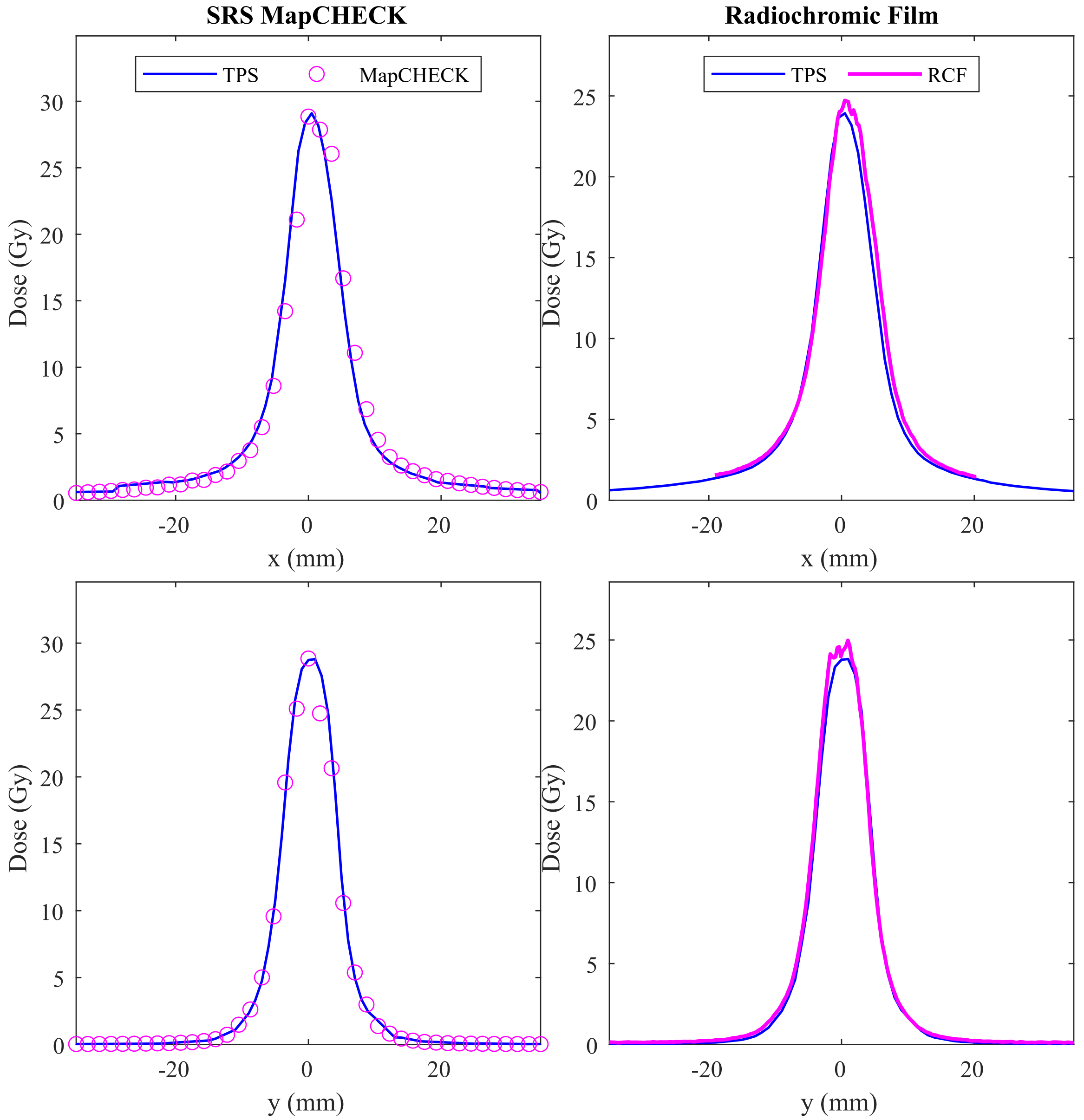

Measurement 20. Plan 14, 4 targets, equivalent diameter 6.8 mm

SRS MapCHECK fraction passing gamma 3%/1 mm = 100.0%

Radichromic film fraction passing gamma 3%/1 mm = 100.0%

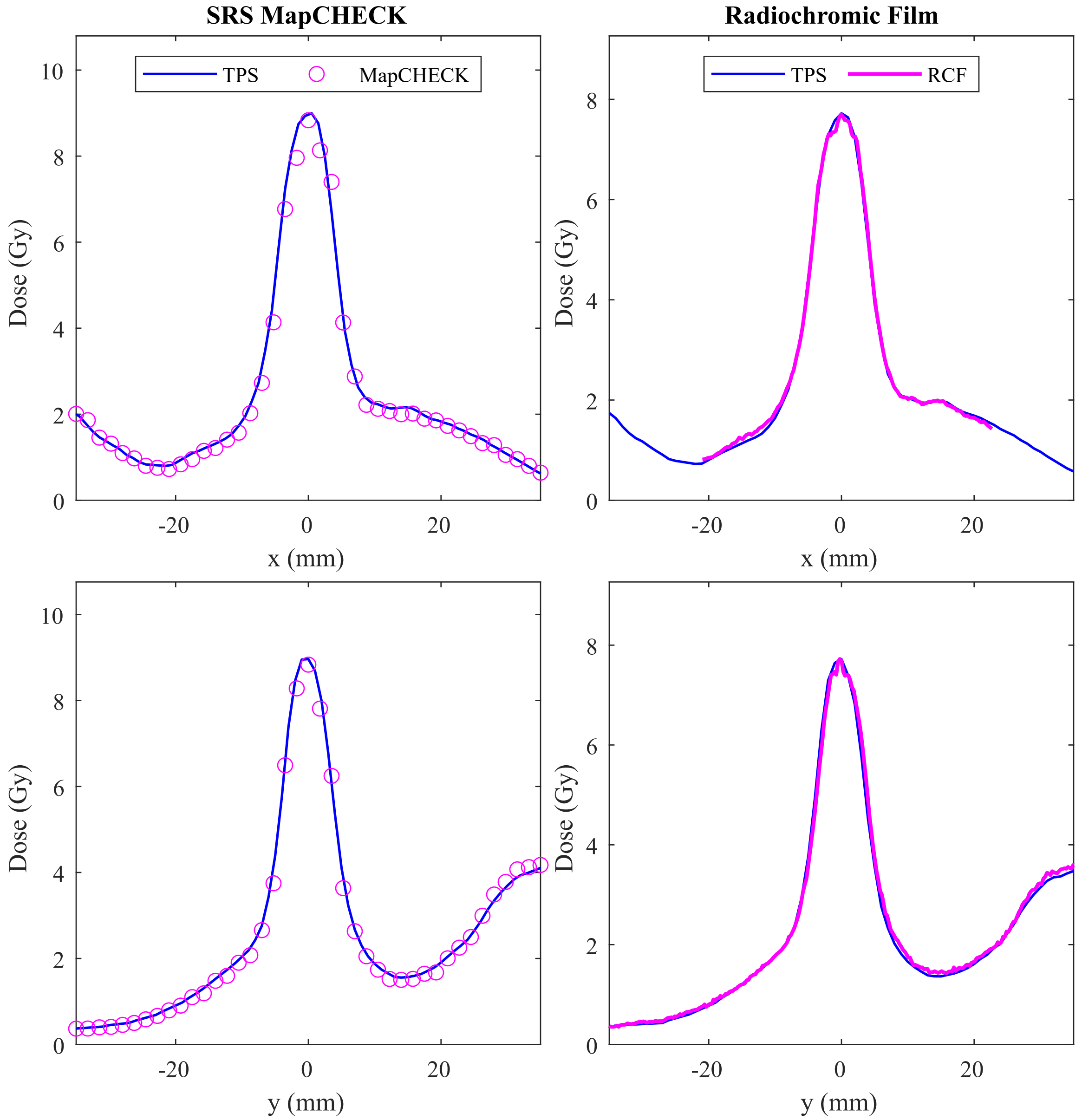

Measurement 21. Plan 14, 4 targets, equivalent diameter 16.2 mm

SRS MapCHECK fraction passing gamma 3%/1 mm = 100.0%

Radichromic film fraction passing gamma 3%/1 mm = 98.1%

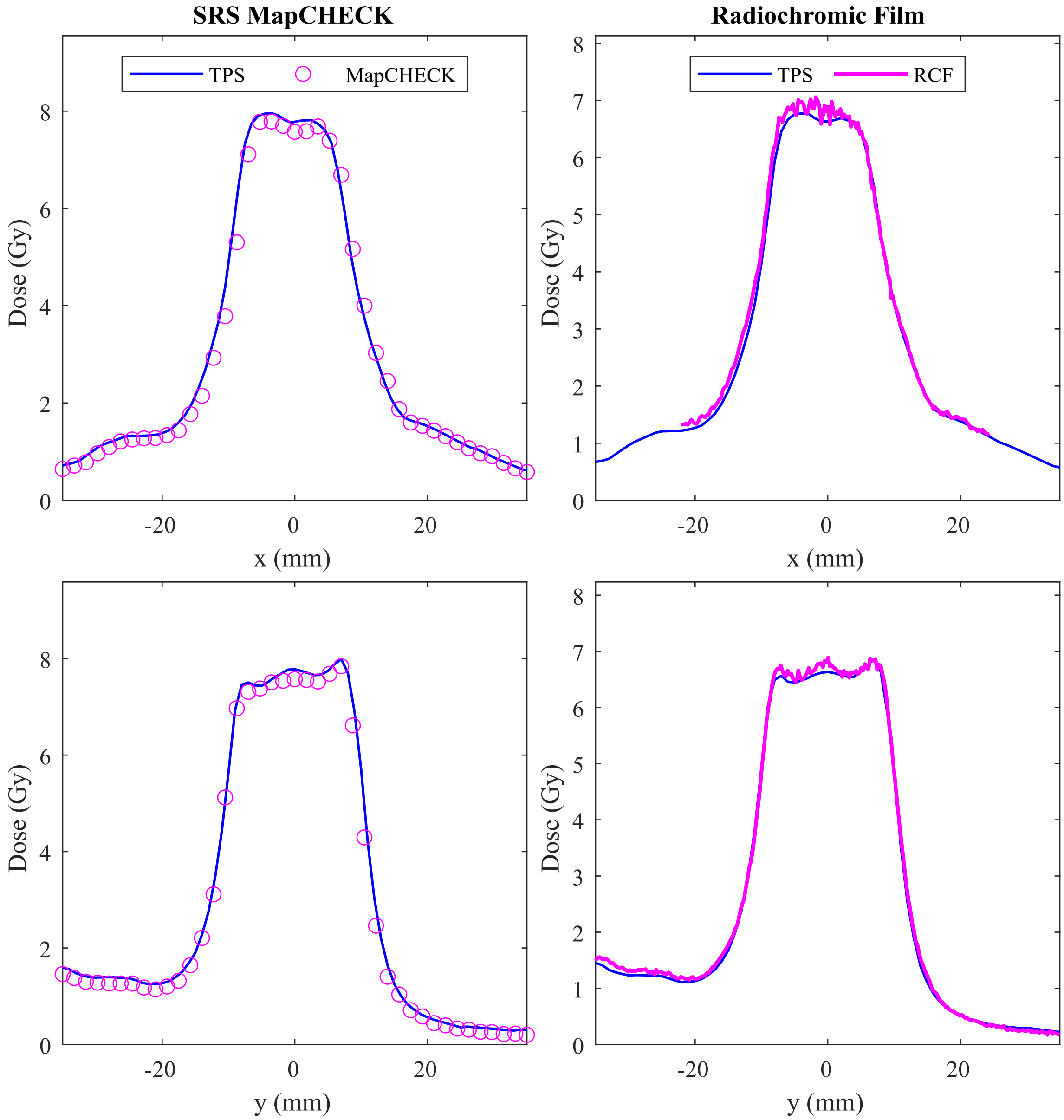

Measurement 22. Plan 15, 2 targets, equivalent diameter 3.6 mm

SRS MapCHECK fraction passing gamma 3%/1 mm = 100.0%

Radichromic film fraction passing gamma 3%/1 mm = 100.0%

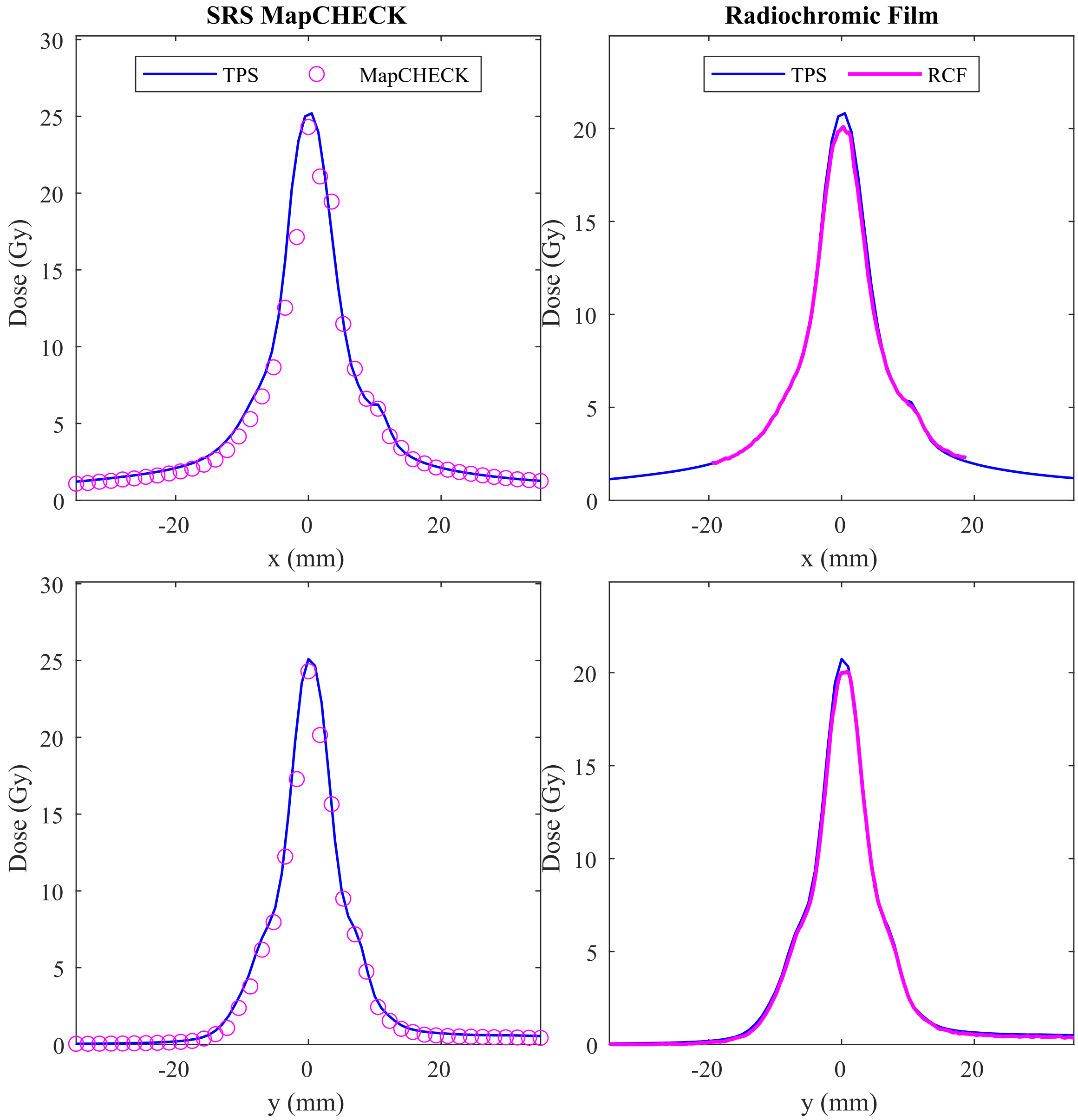

Measurement 23. Plan 15, 2 targets, equivalent diameter 7.0 mm

SRS MapCHECK fraction passing gamma 3%/1 mm = 100.0%

Radichromic film fraction passing gamma 3%/1 mm = 100.0%

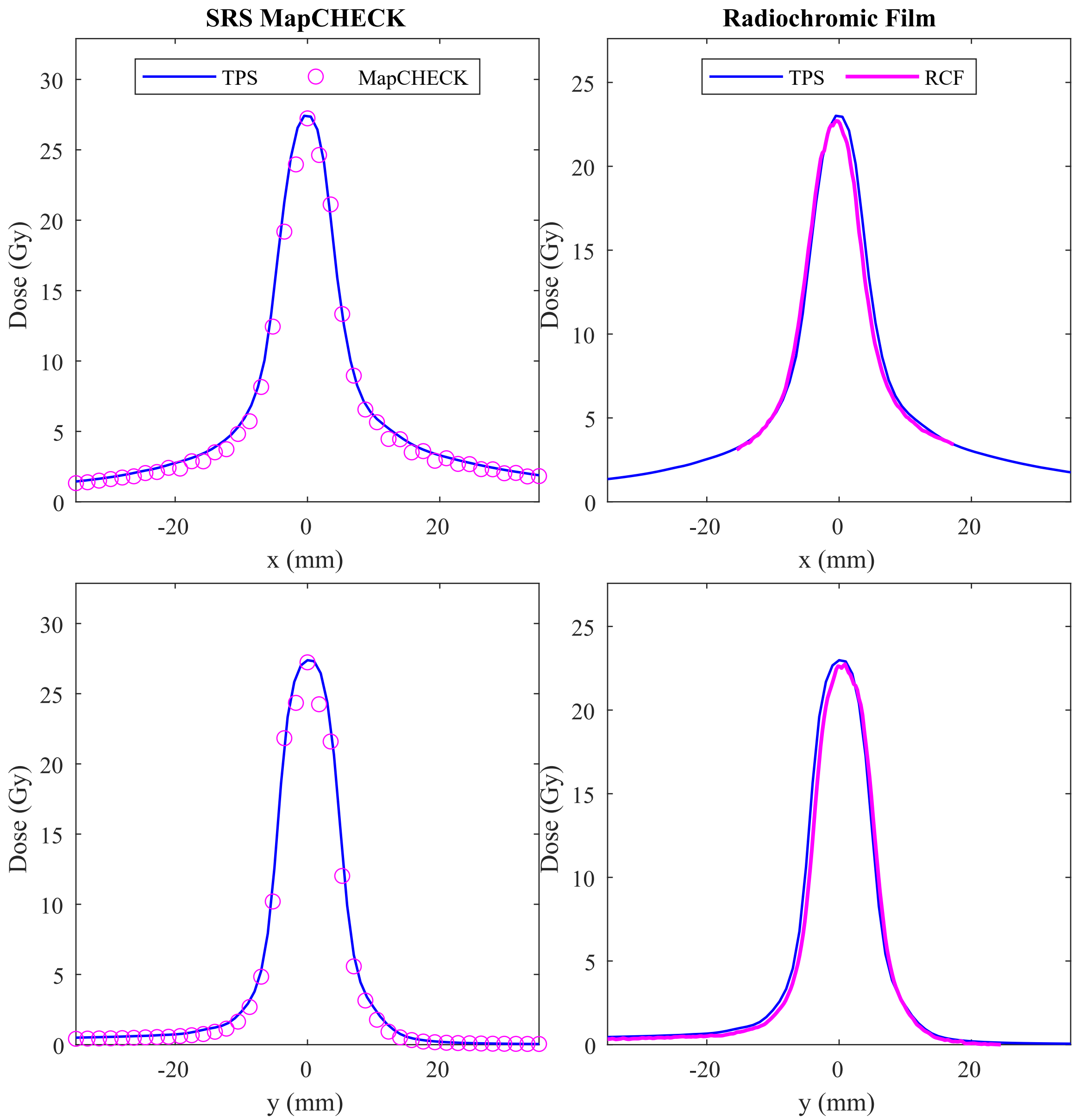

Measurement 24. Plan 16, 7 targets, equivalent diameter 4.1 mm

SRS MapCHECK fraction passing gamma 3%/1 mm = 100.0%

Radichromic film fraction passing gamma 3%/1 mm = 100.0%

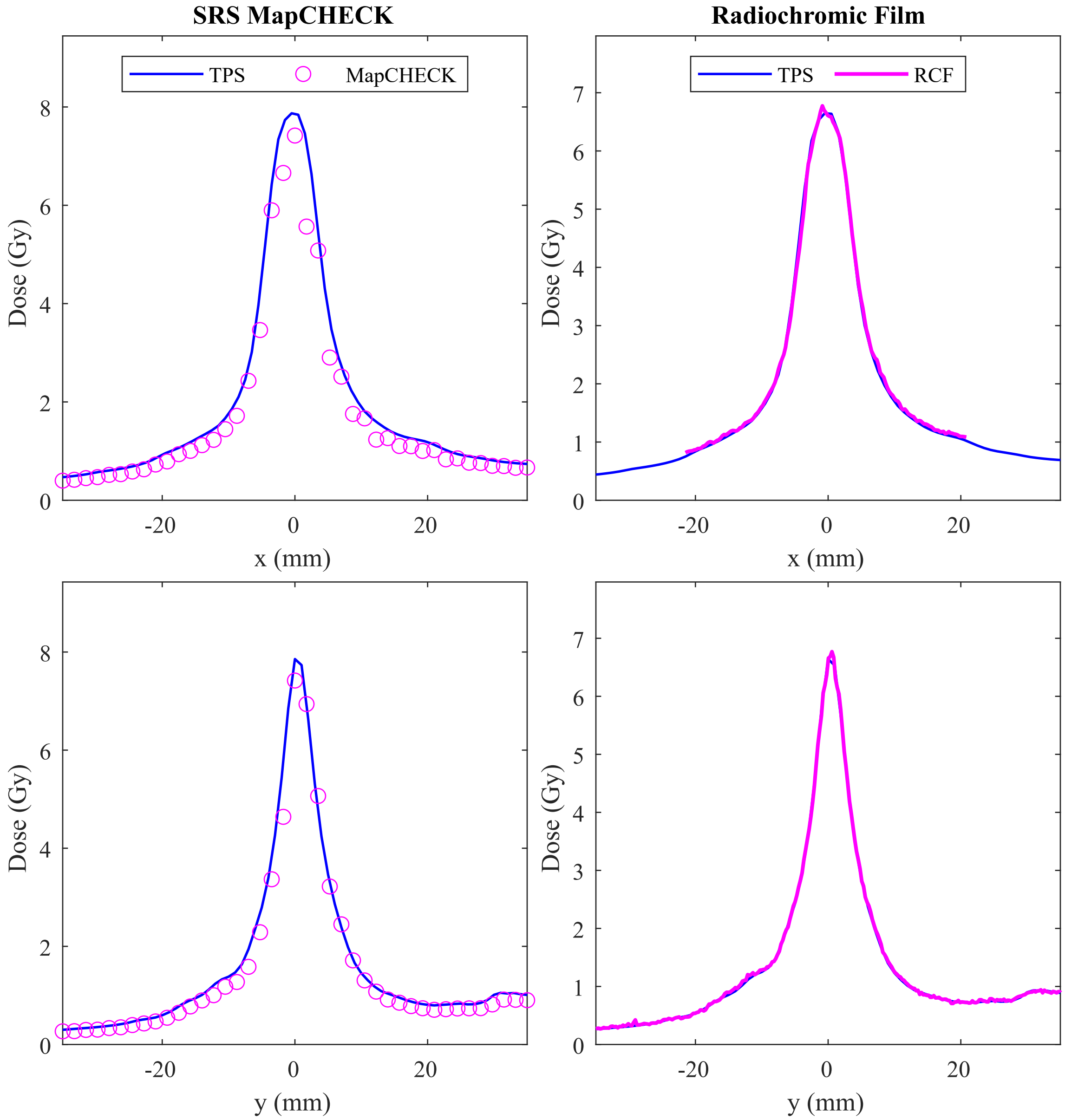

Measurement 25. Plan 16, 7 targets, equivalent diameter 17.5 mm

SRS MapCHECK fraction passing gamma 3%/1 mm = 97.9%

Radichromic film fraction passing gamma 3%/1 mm = 100.0%

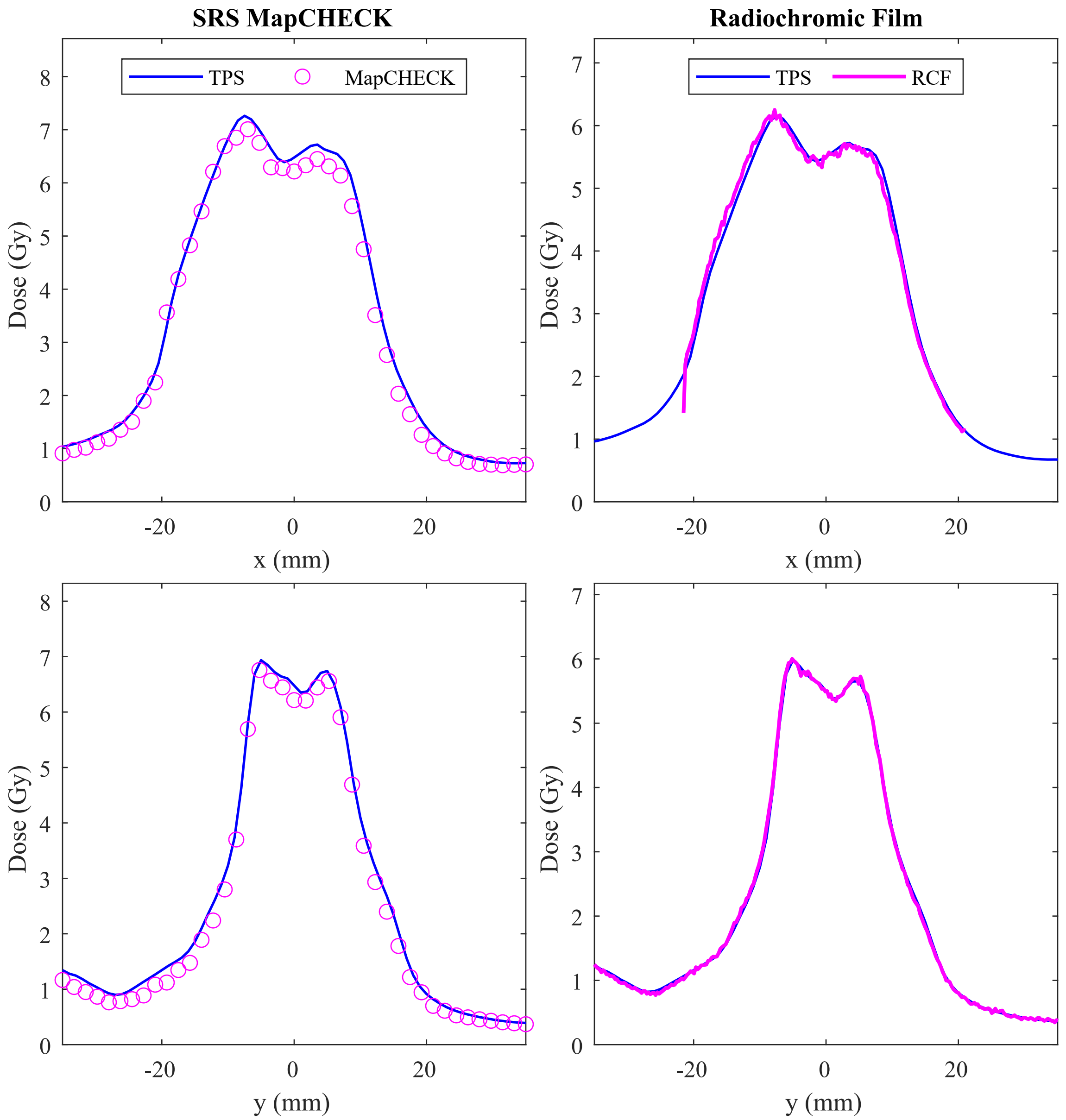

Measurement 26. Plan 17, 7 targets, equivalent diameter 3.9 mm

SRS MapCHECK fraction passing gamma 3%/1 mm = 100.0%

Radichromic film fraction passing gamma 3%/1 mm = 99.9%

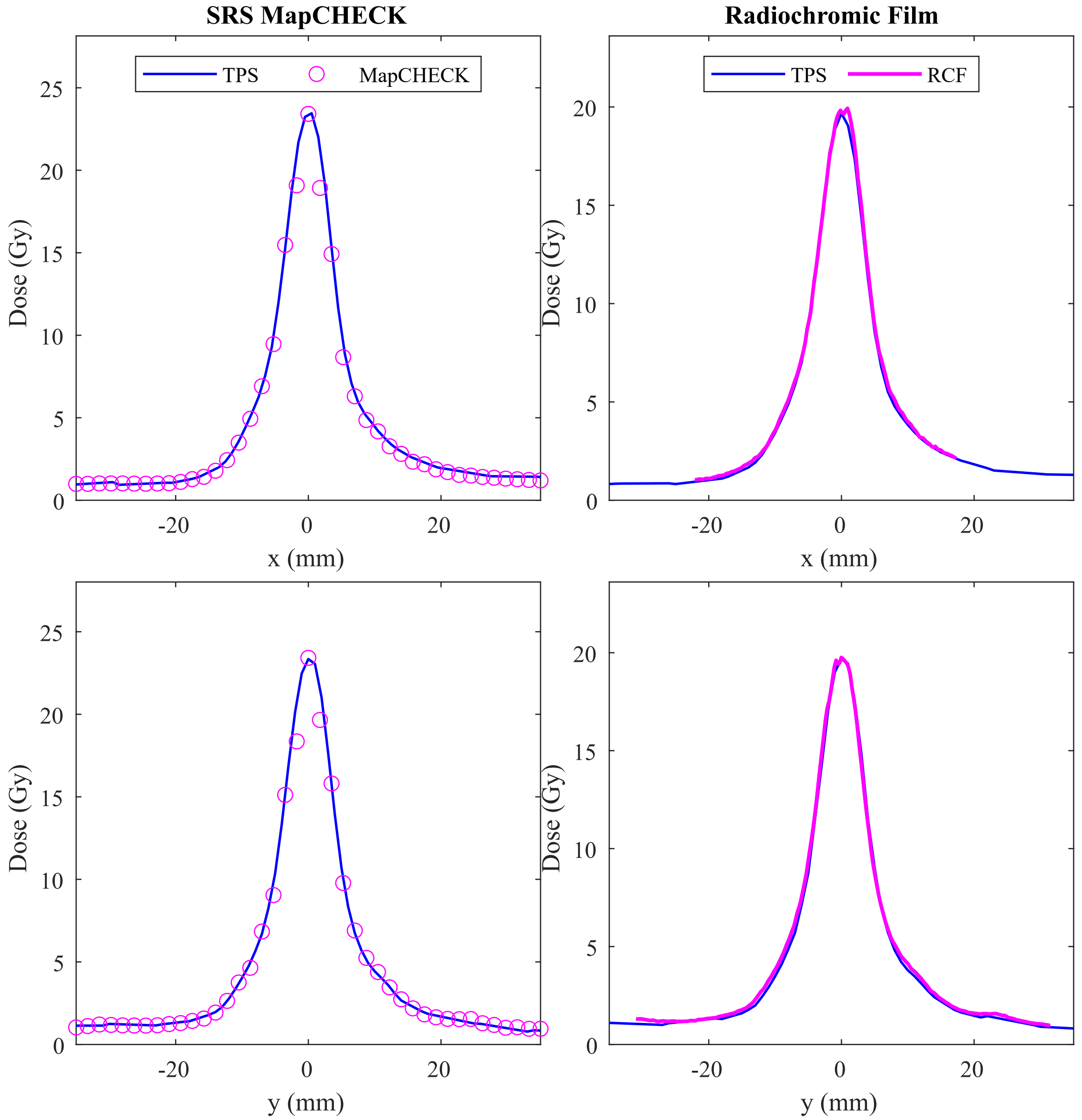

Measurement 27. Plan 17, 7 targets, equivalent diameter 11.1 mm

SRS MapCHECK fraction passing gamma 3%/1 mm = 99.5%

Radichromic film fraction passing gamma 3%/1 mm = 99.5%

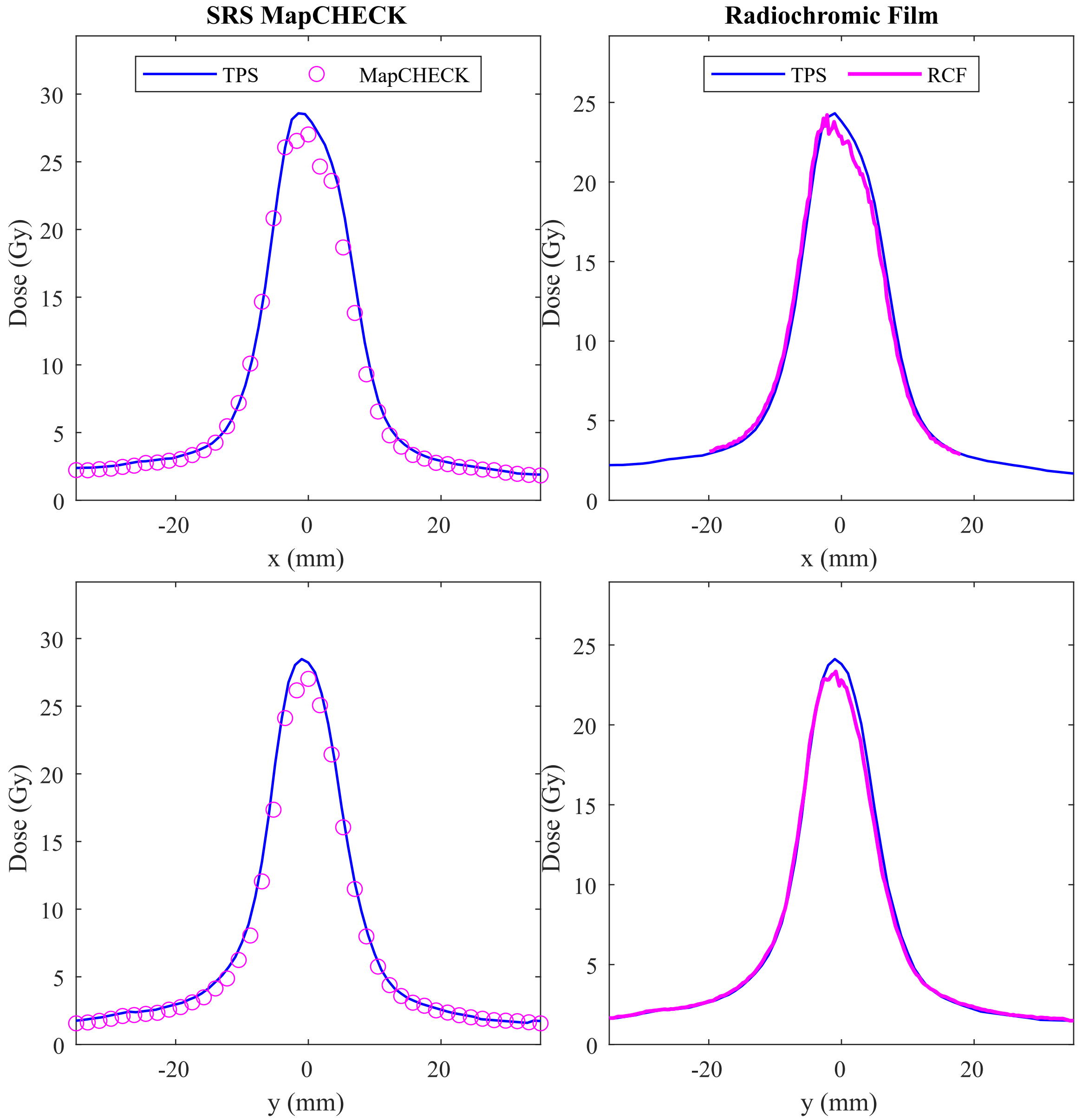

Measurement 28. Plan 18, single target, equivalent diameter 19.0 mm

SRS MapCHECK fraction passing gamma 3%/1 mm = 100.0%

Radichromic film fraction passing gamma 3%/1 mm = 99.5%

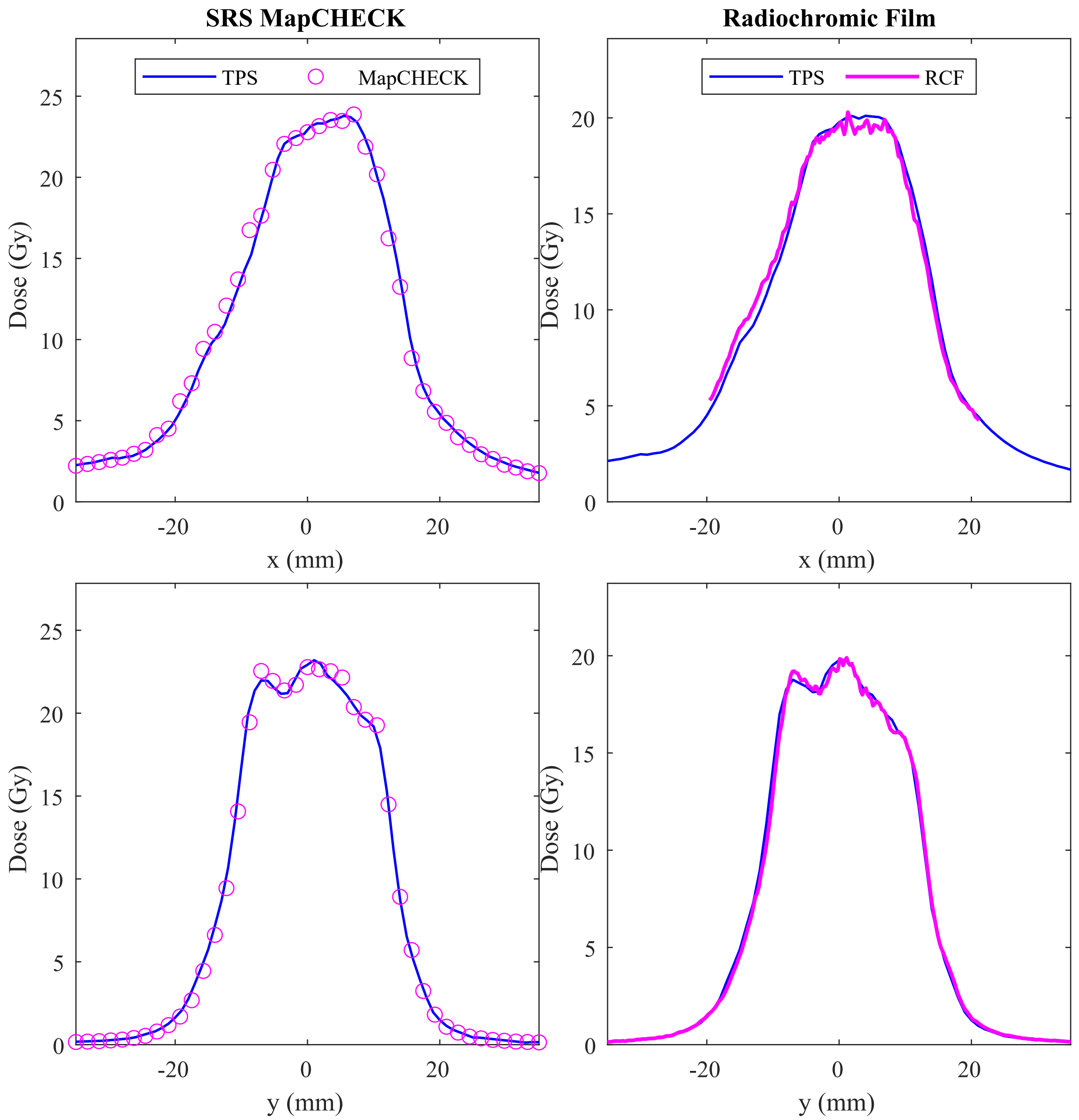

Measurement 29. Plan 19, 9 targets, equivalent diameter 4.2 mm

SRS MapCHECK fraction passing gamma 3%/1 mm = 100.0%

Radichromic film fraction passing gamma 3%/1 mm = 99.2%

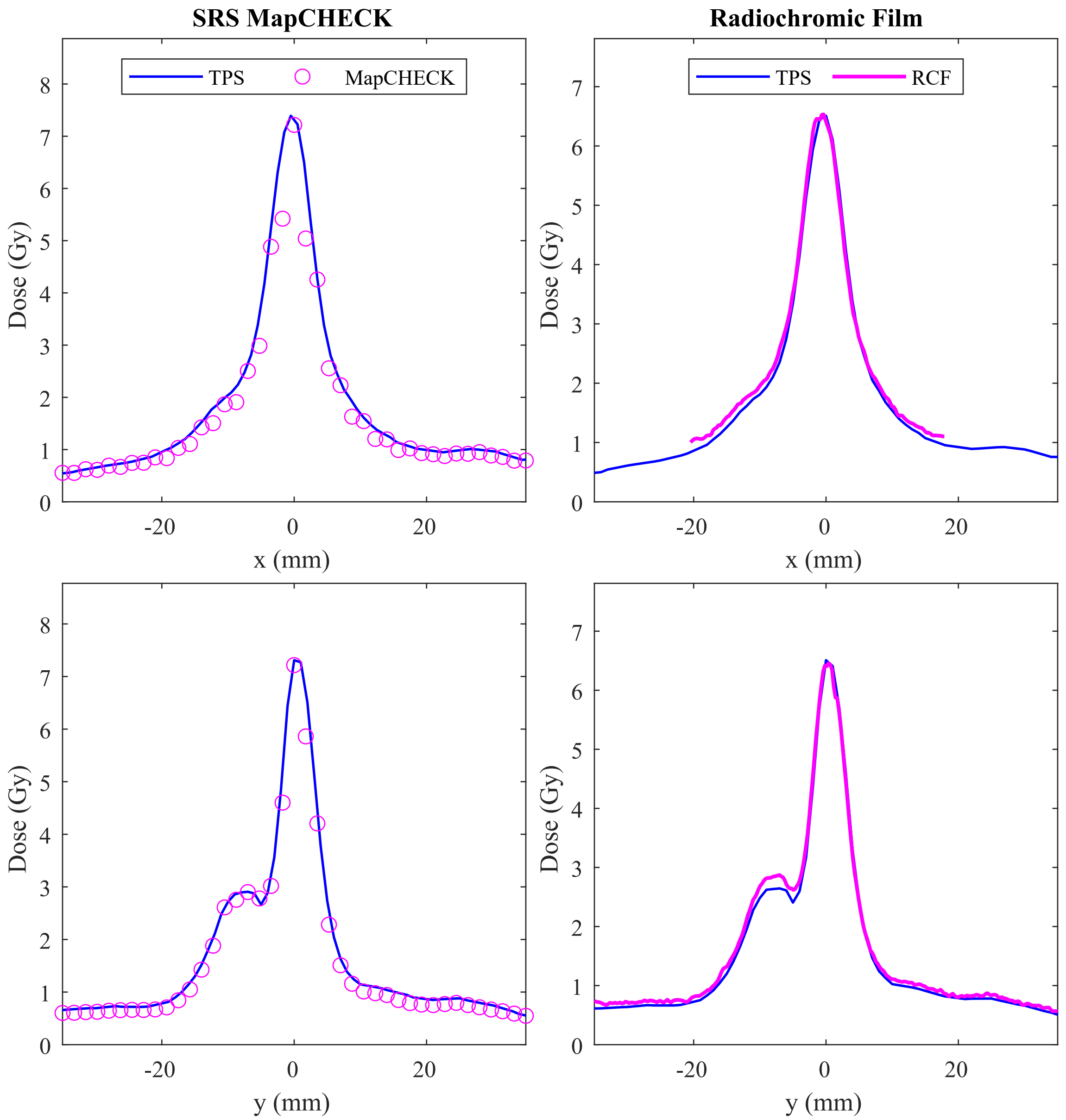

Measurement 30. Plan 19, 9 targets, equivalent diameter 17.1 mm

SRS MapCHECK fraction passing gamma 3%/1 mm = 98.6%

Radichromic film fraction passing gamma 3%/1 mm = 95.8%

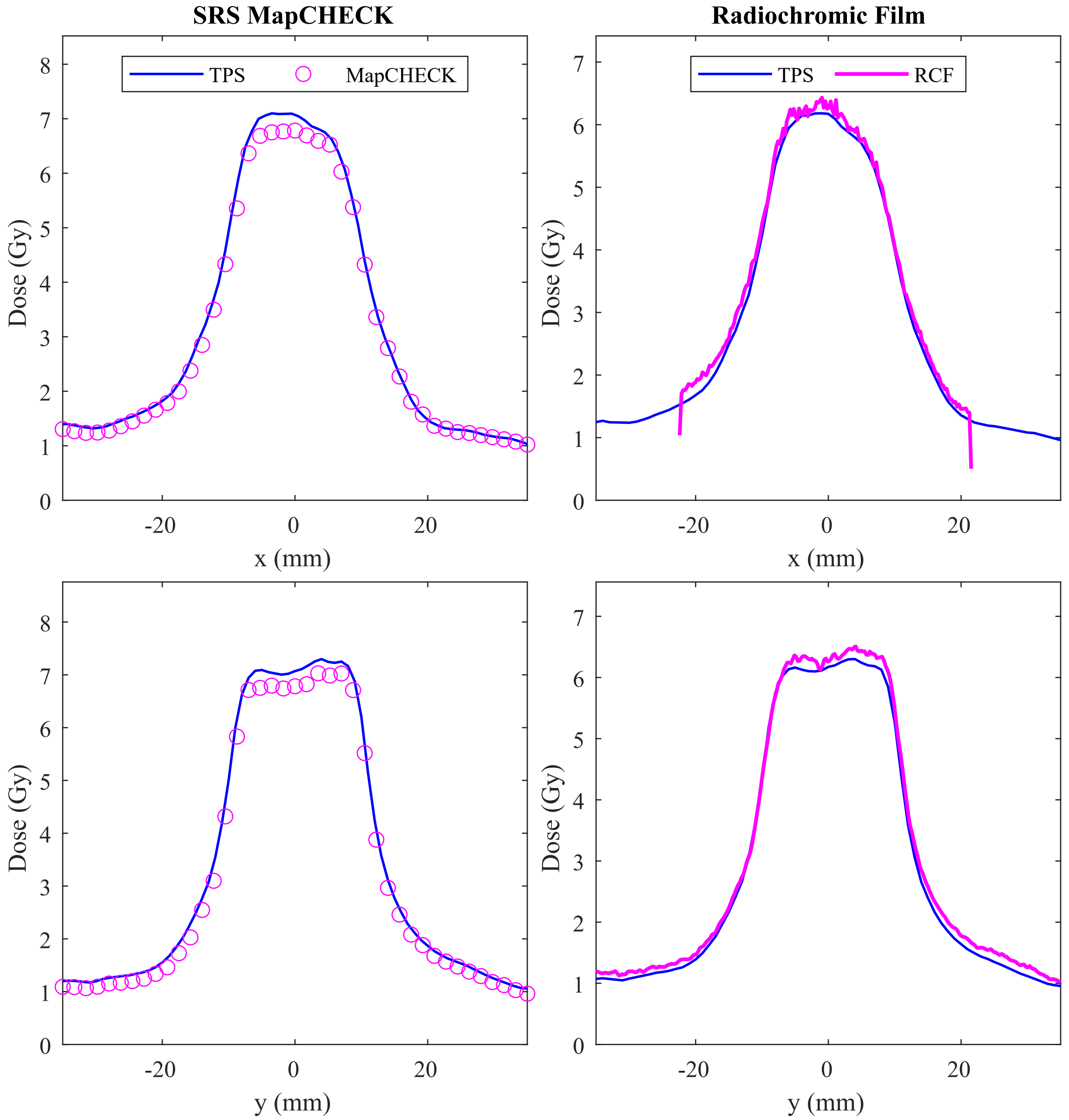

Measurement 31. Plan 20, 4 targets, equivalent diameter 5.7 mm

SRS MapCHECK fraction passing gamma 3%/1 mm = 99.3%

Radichromic film fraction passing gamma 3%/1 mm = 100.0%

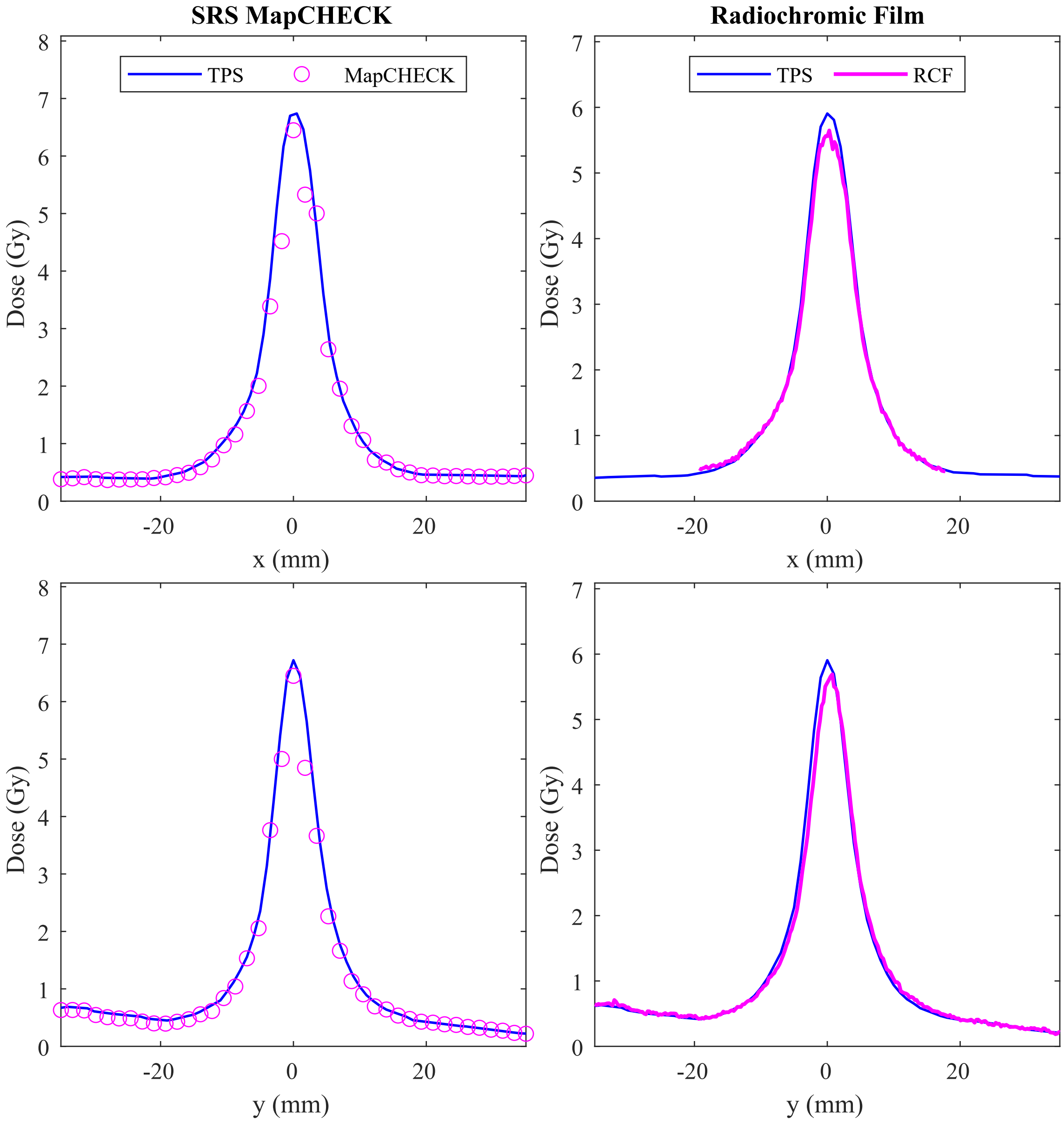

Measurement 32. Plan 20, 4 targets, equivalent diameter 14.7 mm

SRS MapCHECK fraction passing gamma 3%/1 mm = 100.0%

Radichromic film fraction passing gamma 3%/1 mm = 99.4%

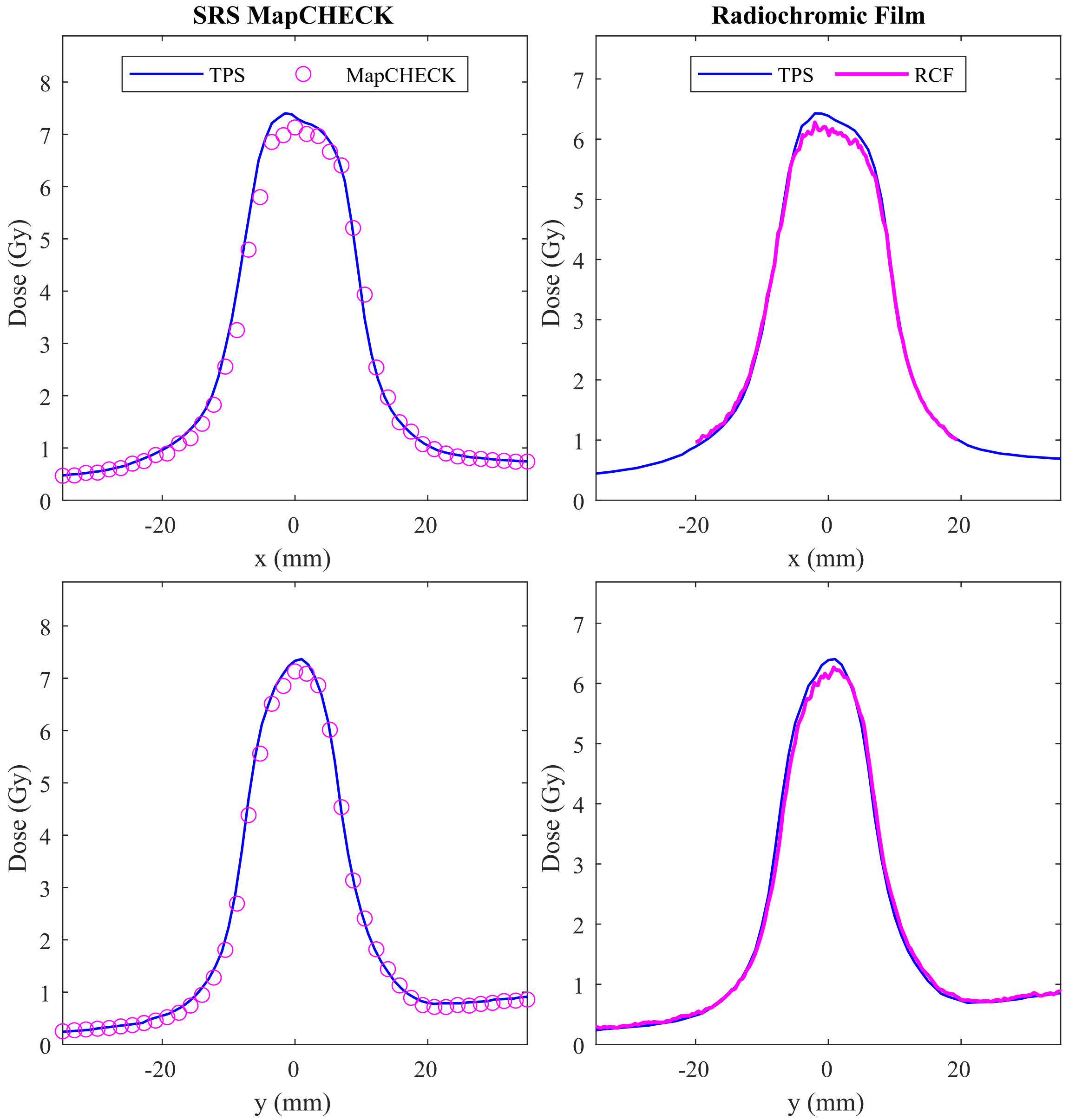

Measurement 33. Plan 21, single target, equivalent diameter 10.5 mm

SRS MapCHECK fraction passing gamma 3%/1 mm = 100.0%

Radichromic film fraction passing gamma 3%/1 mm = 99.5%

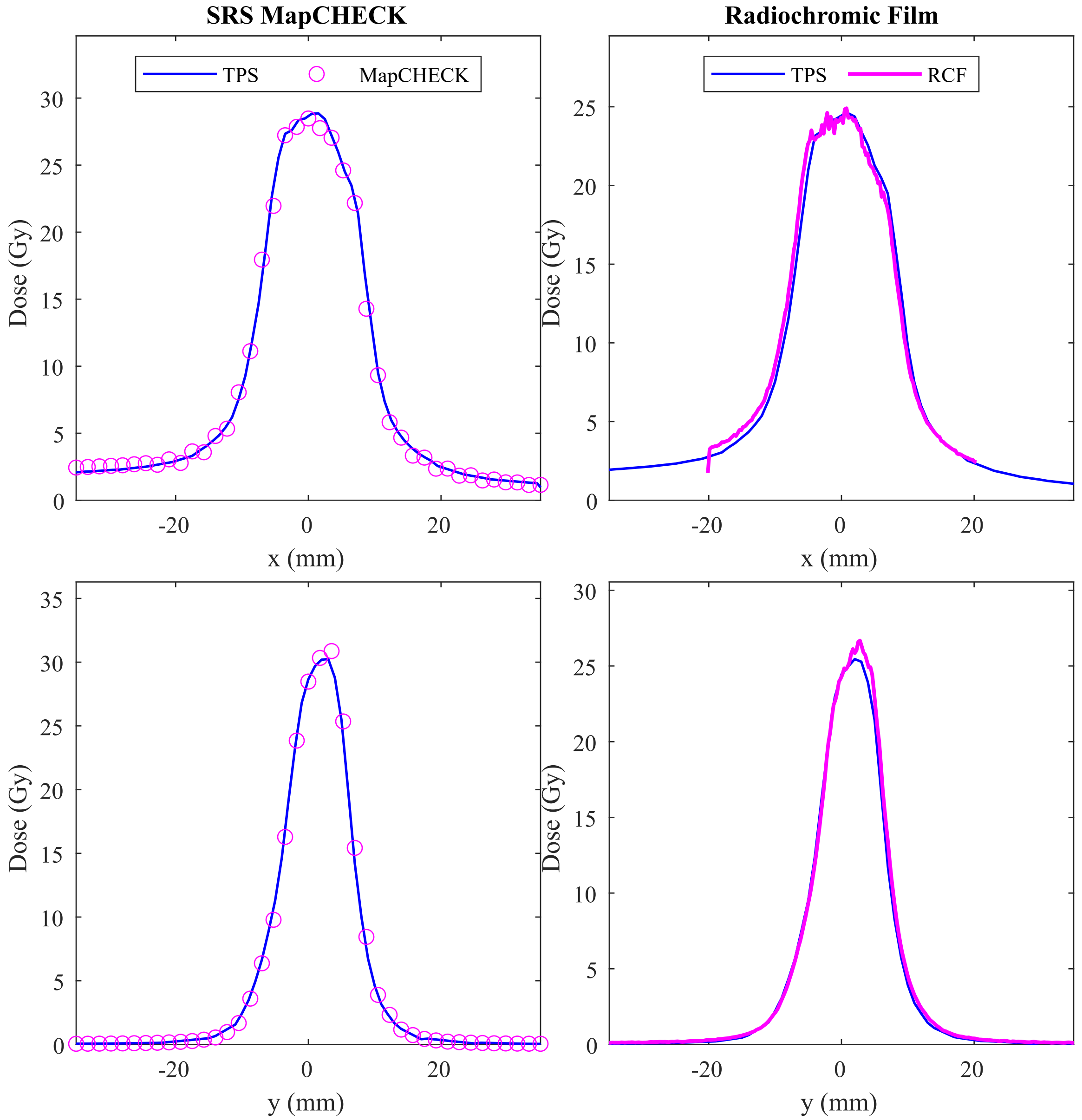

Measurement 34. Plan 22, 6 targets, equivalent diameter 4.0 mm

SRS MapCHECK fraction passing gamma 3%/1 mm = 100.0%

Radichromic film fraction passing gamma 3%/1 mm = 100.0%

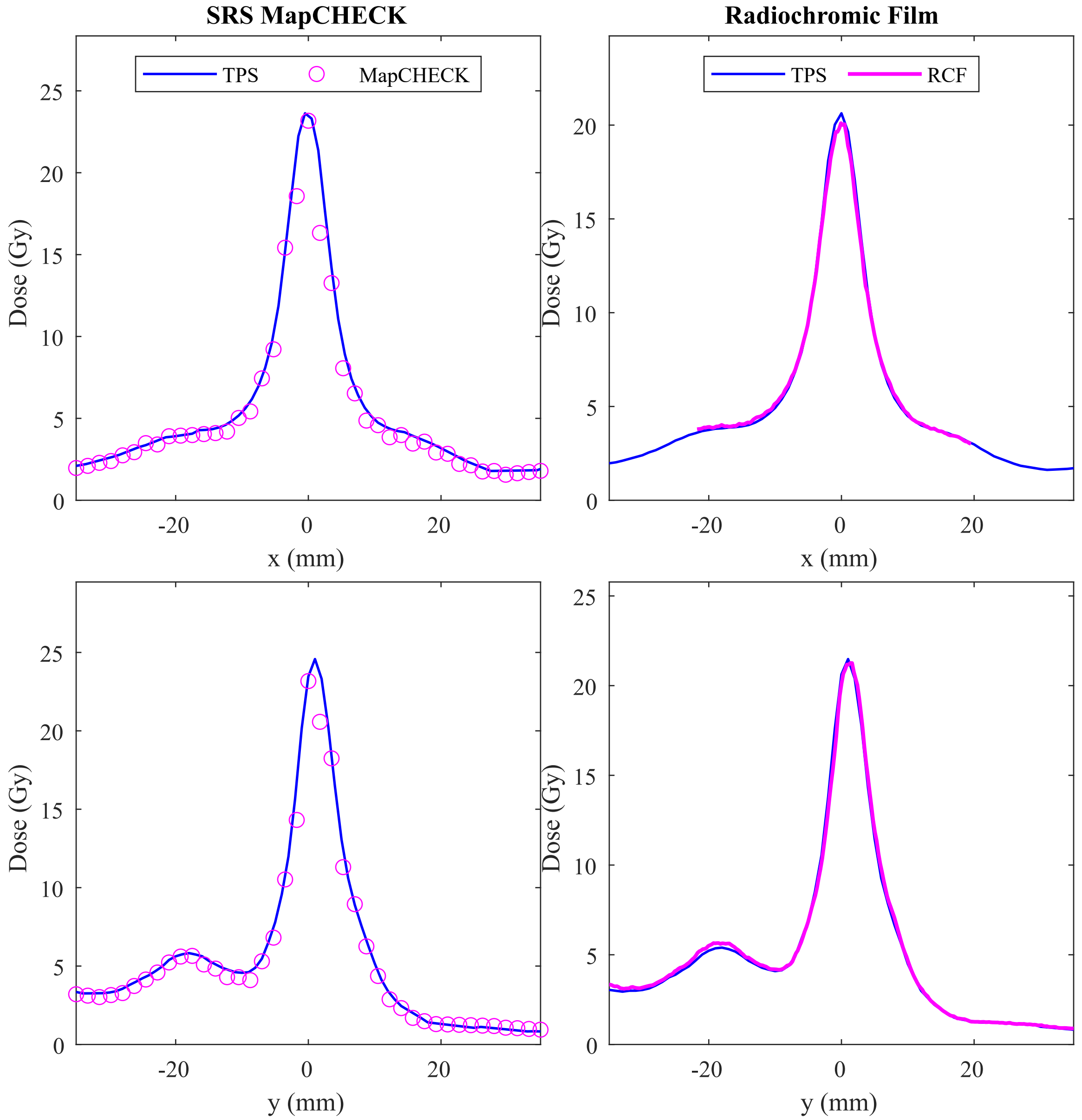

Measurement 35. Plan 22, 6 targets, equivalent diameter 8.7 mm

SRS MapCHECK fraction passing gamma 3%/1 mm = 100.0%

Radichromic film fraction passing gamma 3%/1 mm = 99.8%

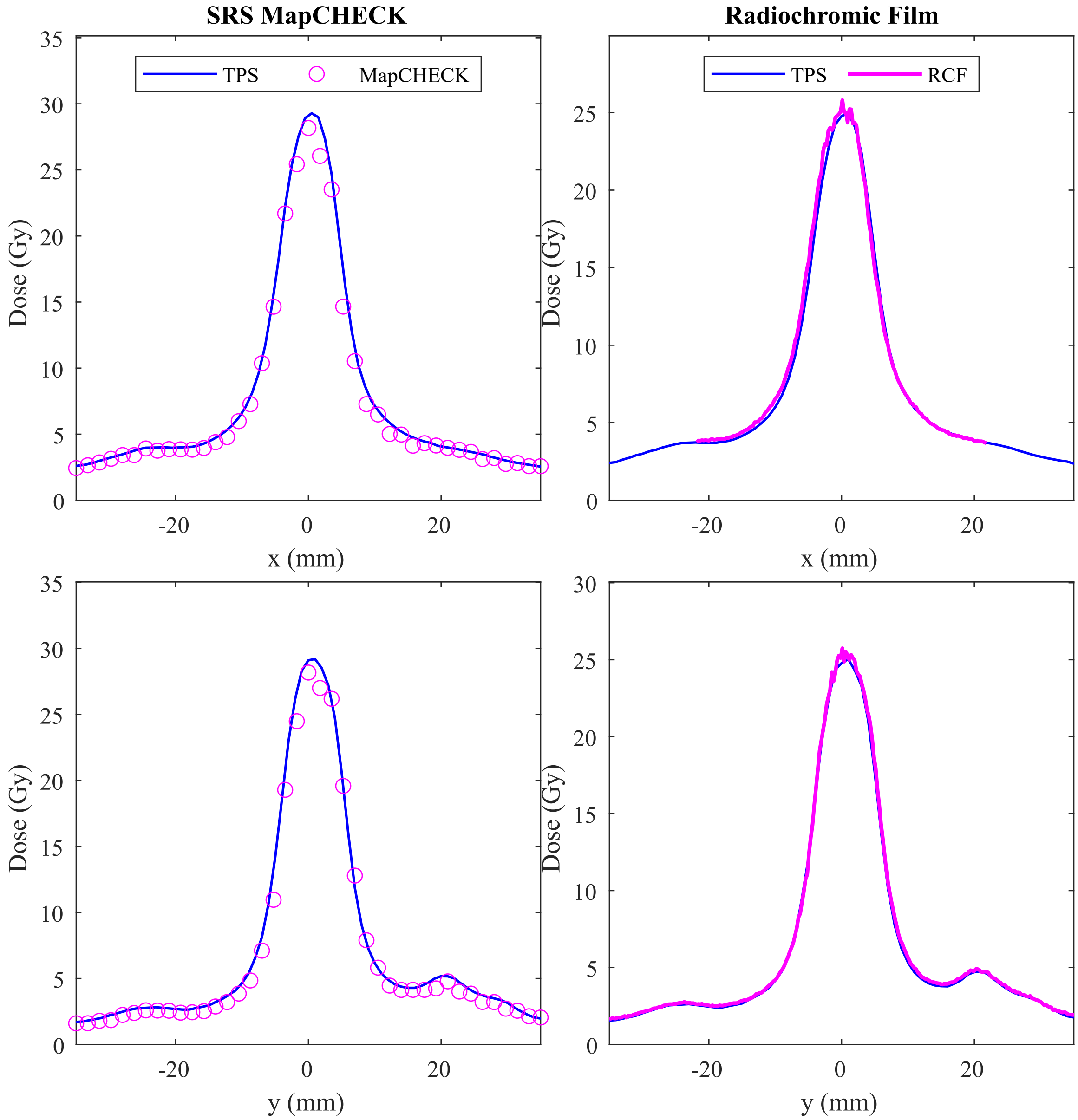

Measurement 36. Plan 23, 9 targets, equivalent diameter 3.0 mm

SRS MapCHECK fraction passing gamma 3%/1 mm = 95.3%

Radichromic film fraction passing gamma 3%/1 mm = 100.0%

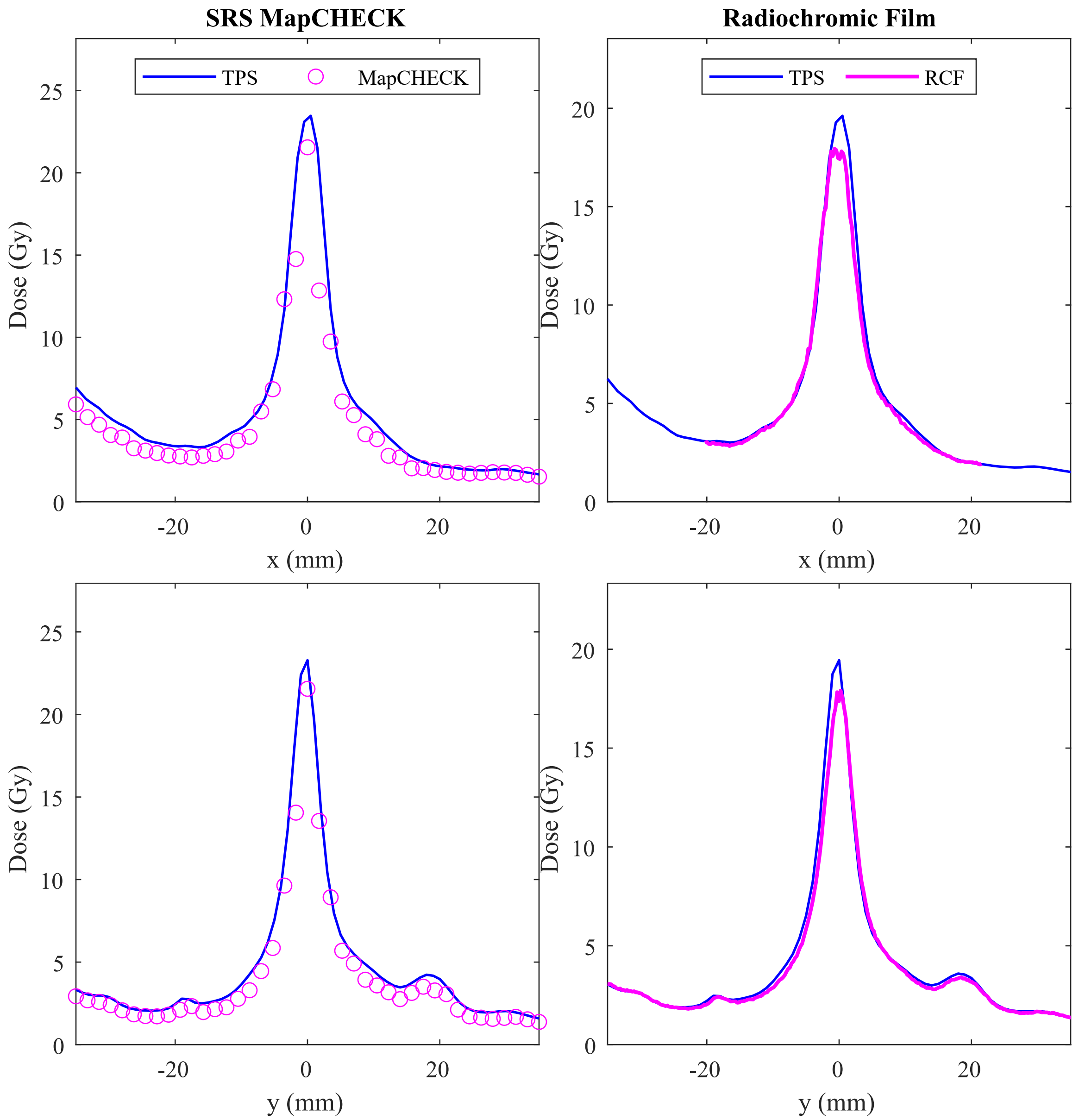

Measurement 37. Plan 23, 9 targets, equivalent diameter 6.5 mm

SRS MapCHECK fraction passing gamma 3%/1 mm = 100.0%

Radichromic film fraction passing gamma 3%/1 mm = 100.0%

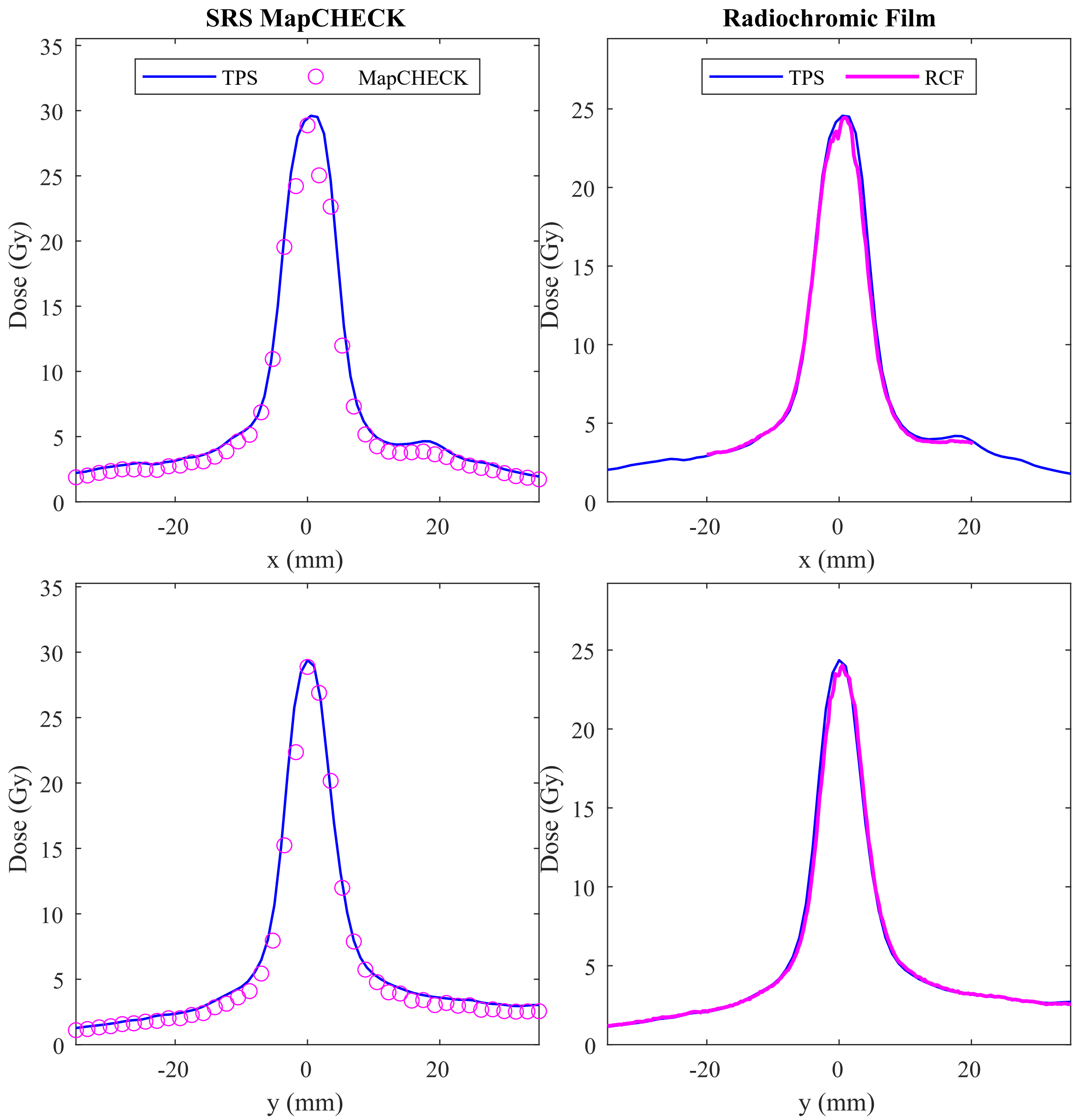

Measurement 38. Plan 24, single target, equivalent diameter 6.0 mm

SRS MapCHECK fraction passing gamma 3%/1 mm = 100.0%

Radichromic film fraction passing gamma 3%/1 mm = 100.0%

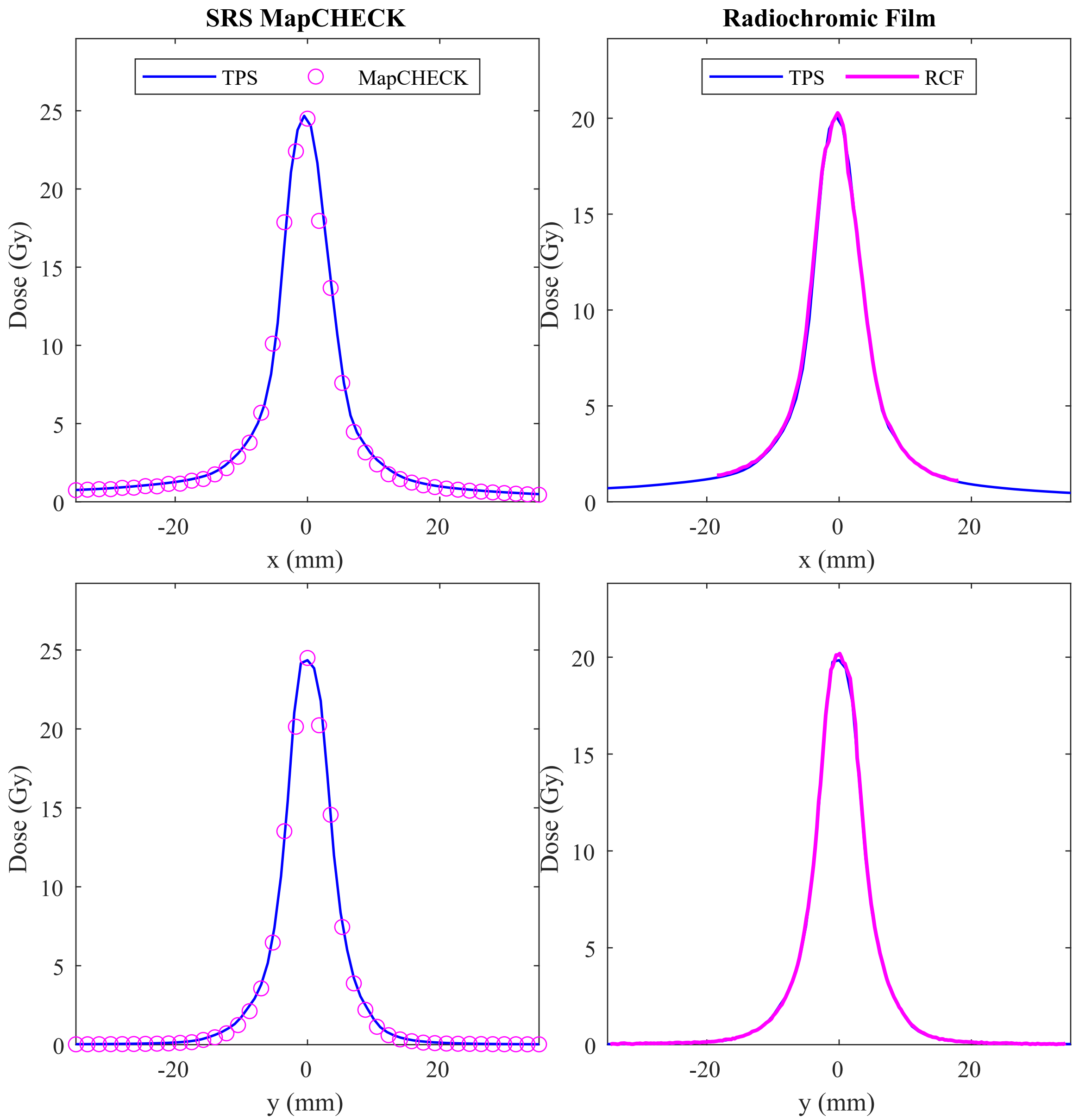

Measurement 39. Plan 25, single target, equivalent diameter 3.0 mm

SRS MapCHECK fraction passing gamma 3%/1 mm = 100.0%

Radichromic film fraction passing gamma 3%/1 mm = 100.0%

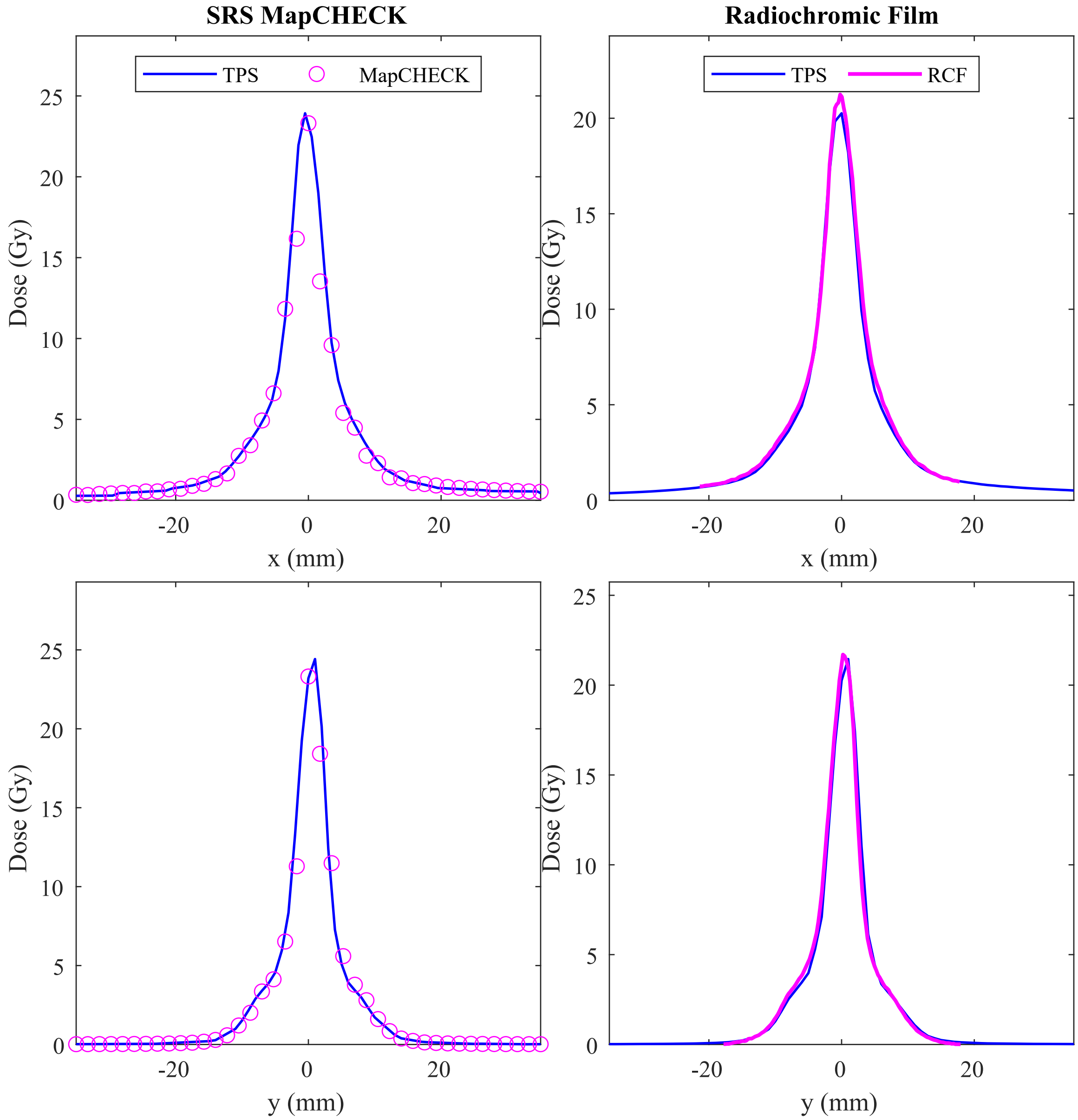

Measurement 40. Plan 26, 5 targets, equivalent diameter 3.0 mm

SRS MapCHECK fraction passing gamma 3%/1 mm = 100.0%

Radichromic film fraction passing gamma 3%/1 mm = 100.0%

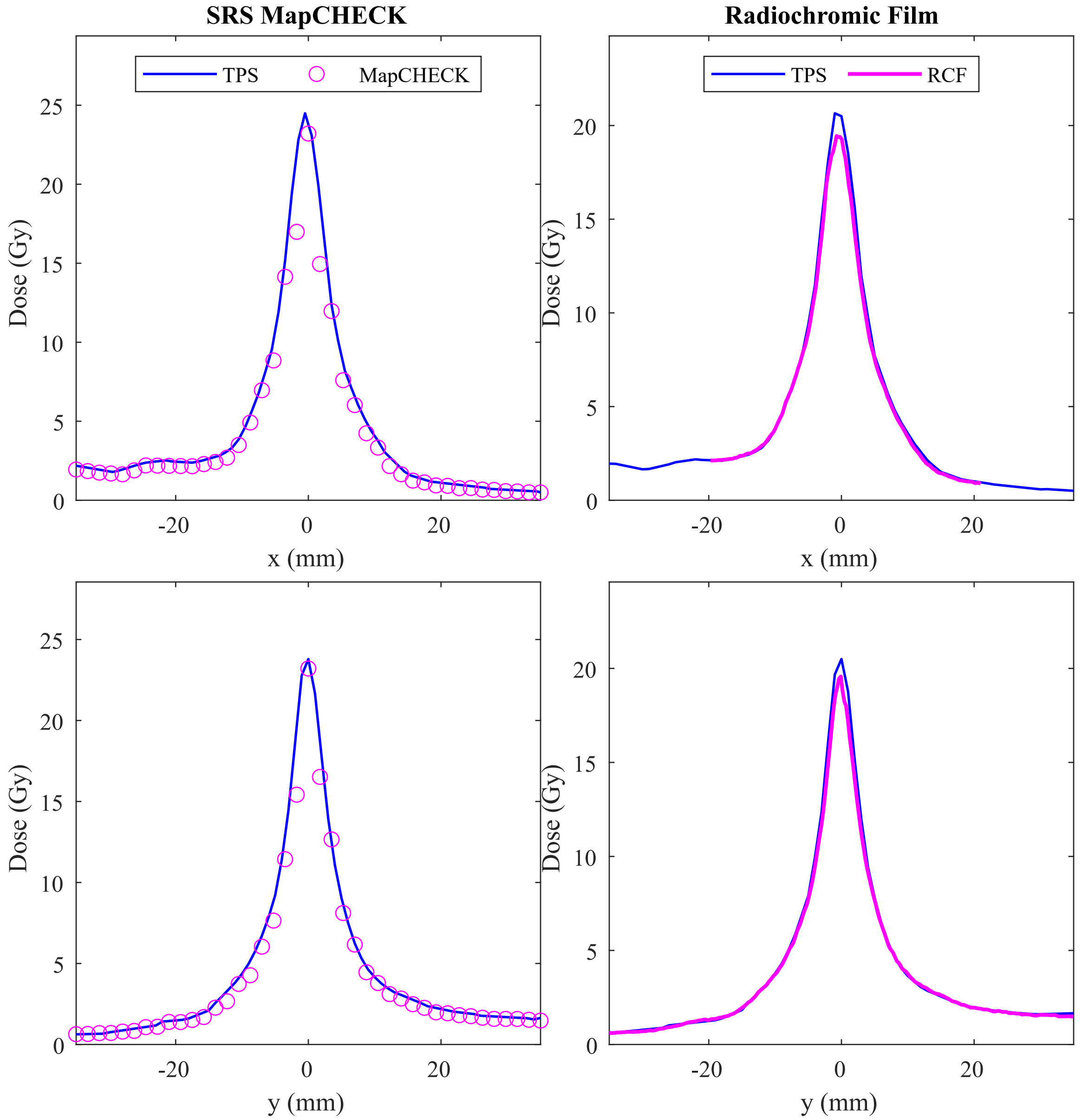

Measurement 41. Plan 26, 5 targets, equivalent diameter 11.8 mm

SRS MapCHECK fraction passing gamma 3%/1 mm = 98.1%

Radichromic film fraction passing gamma 3%/1 mm = 99.2%

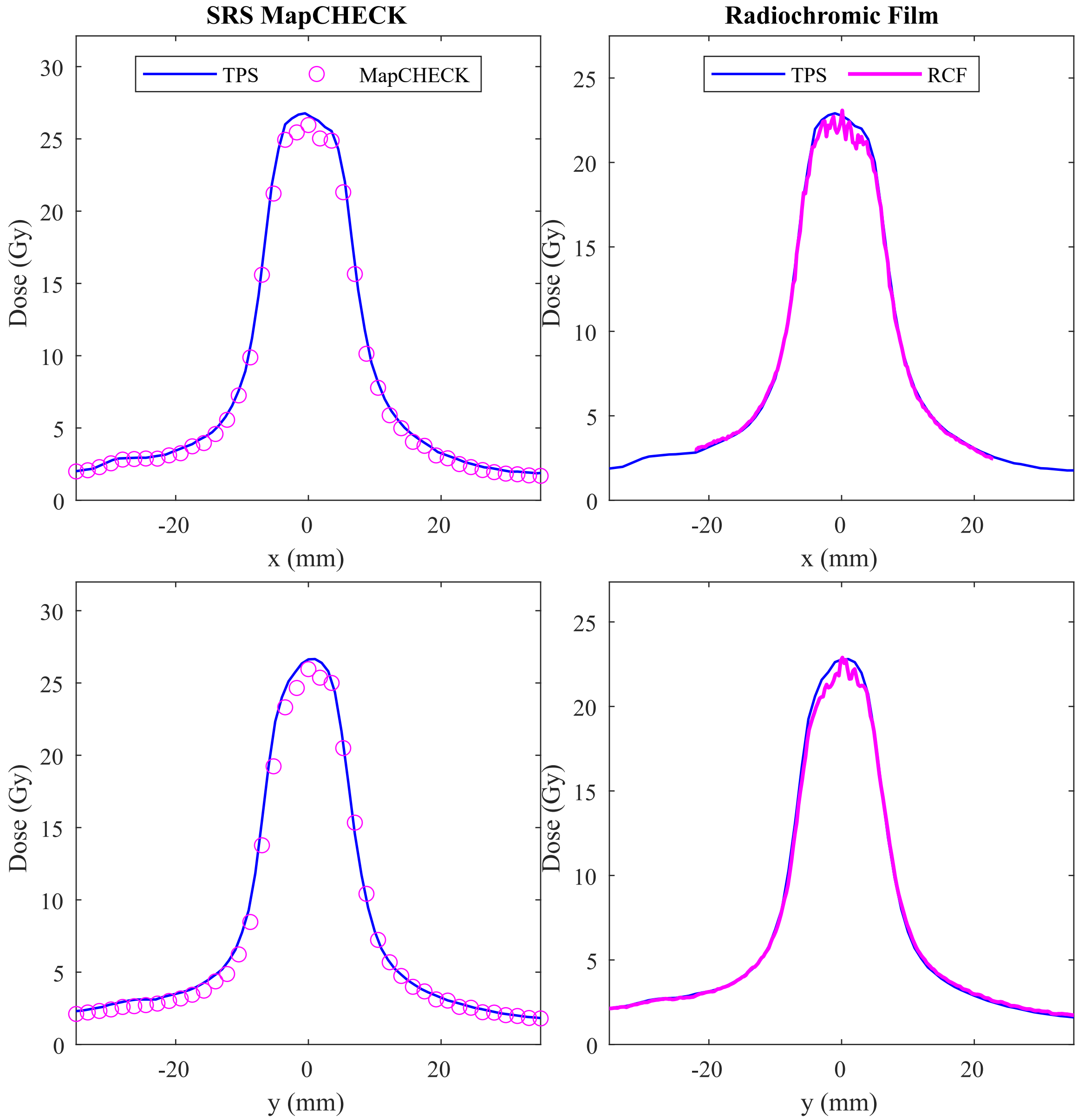

Measurement 42. Plan 27, single target, equivalent diameter 16.2 mm

SRS MapCHECK fraction passing gamma 3%/1 mm = 100.0%

Radichromic film fraction passing gamma 3%/1 mm = 99.9%

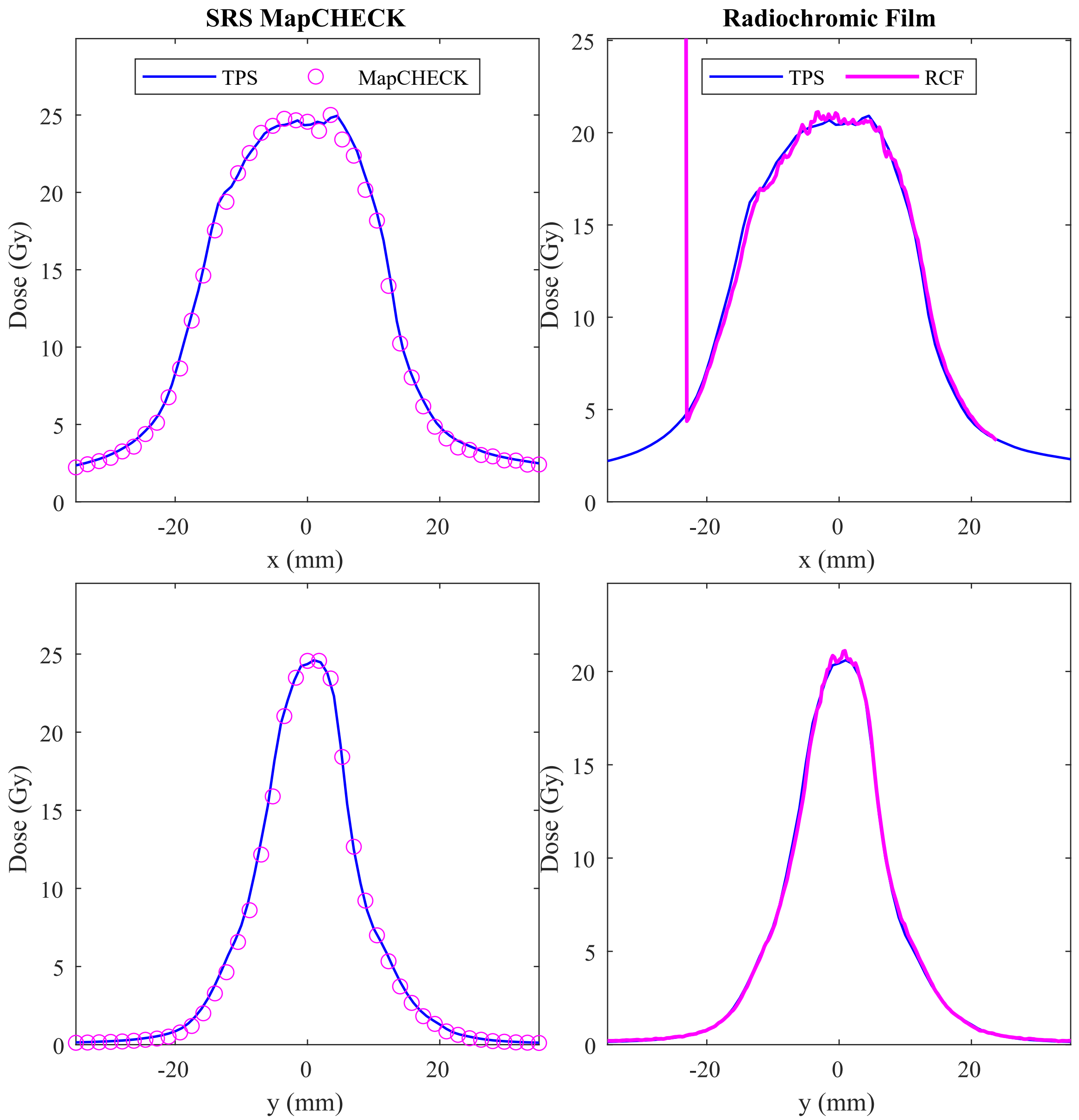

Measurement 43. Plan 28, single target, equivalent diameter 8.6 mm

SRS MapCHECK fraction passing gamma 3%/1 mm = 100.0%

Radichromic film fraction passing gamma 3%/1 mm = 99.0%

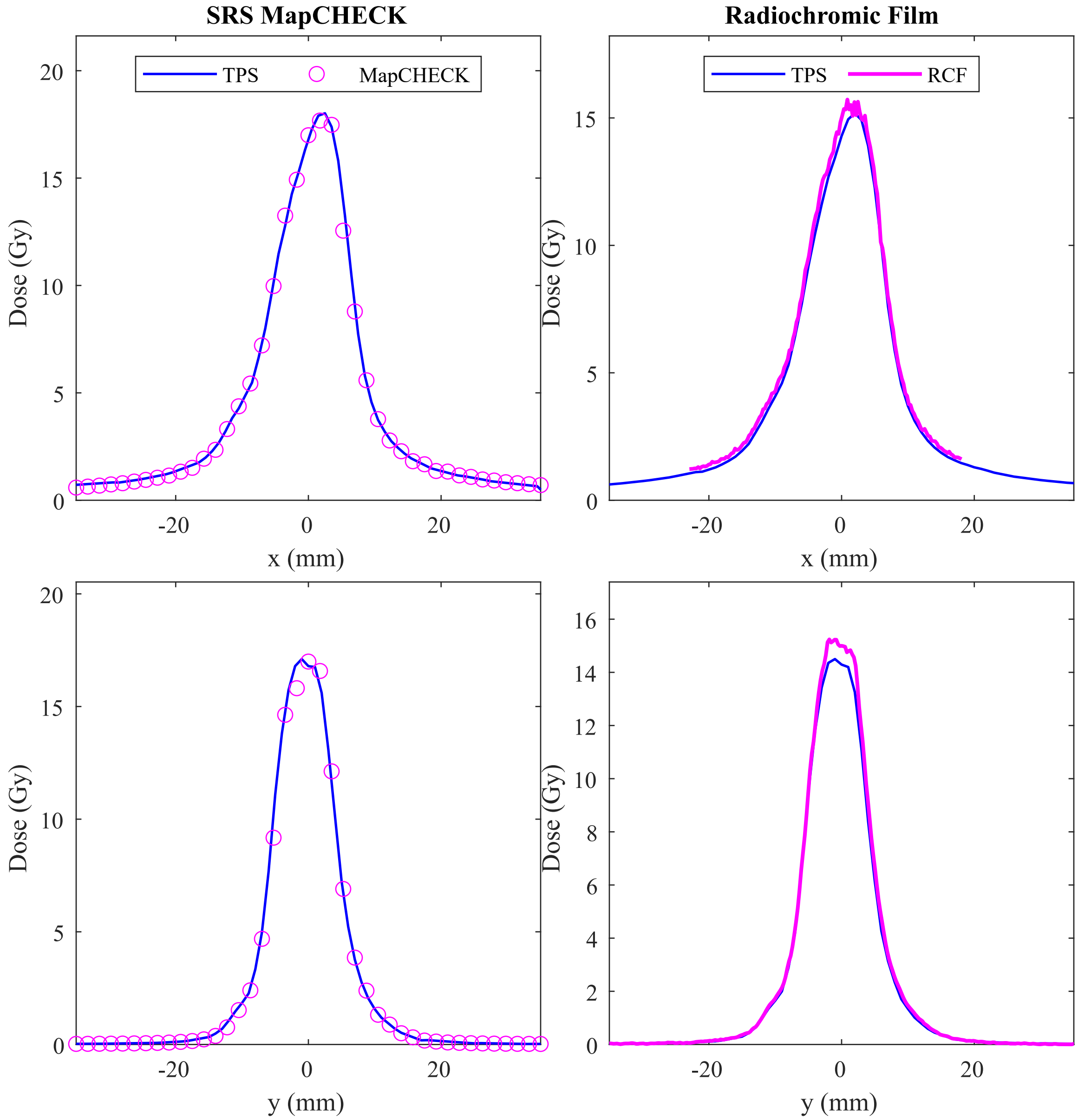

Measurement 44. Plan 29, single target, equivalent diameter 23.2 mm

SRS MapCHECK fraction passing gamma 3%/1 mm = 100.0%

Radichromic film fraction passing gamma 3%/1 mm = 96.9%

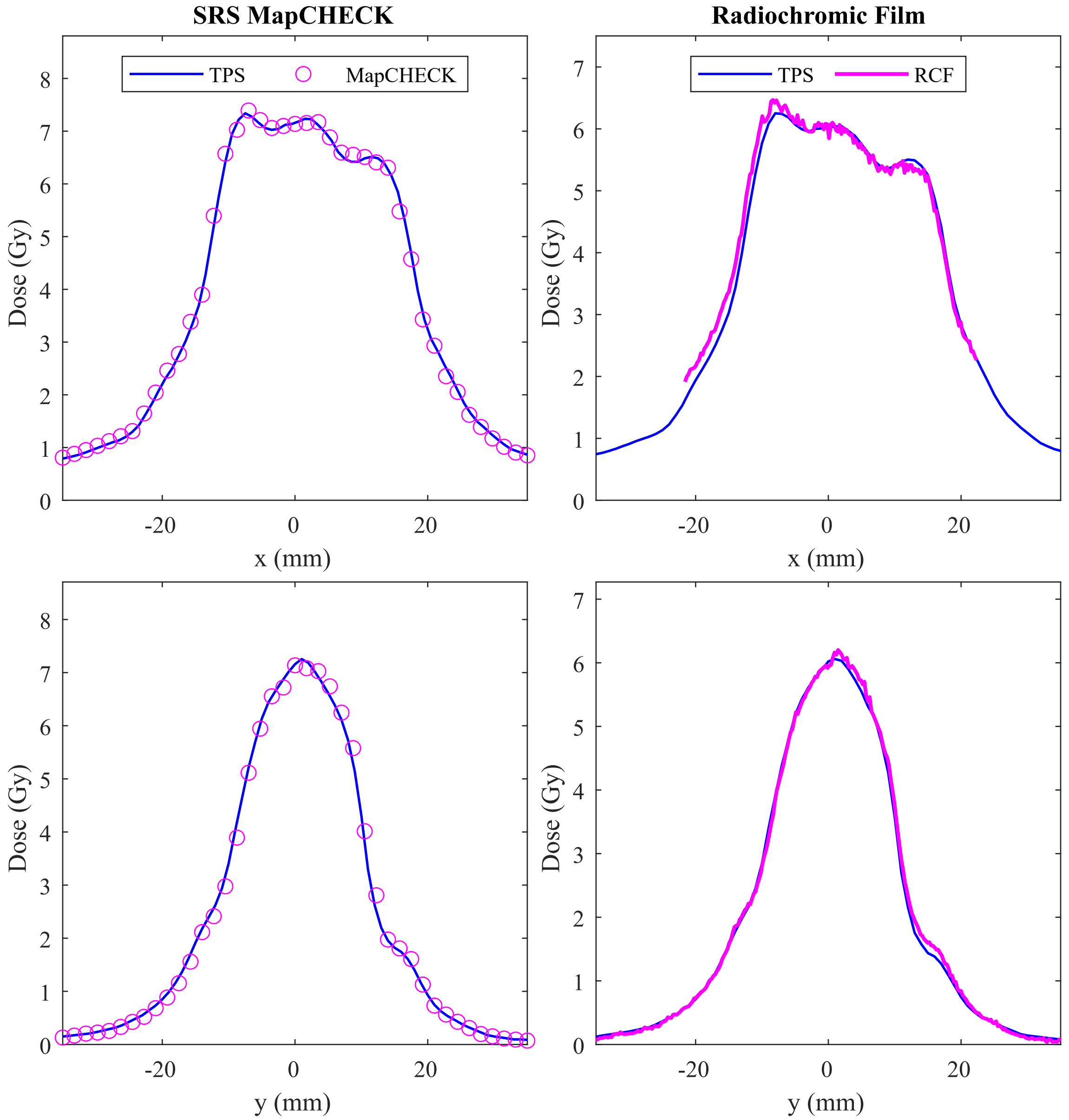

Measurement 45. Plan 30, 4 targets, equivalent diameter 4.9 mm

SRS MapCHECK fraction passing gamma 3%/1 mm = 100.0%

Radichromic film fraction passing gamma 3%/1 mm = 99.9%

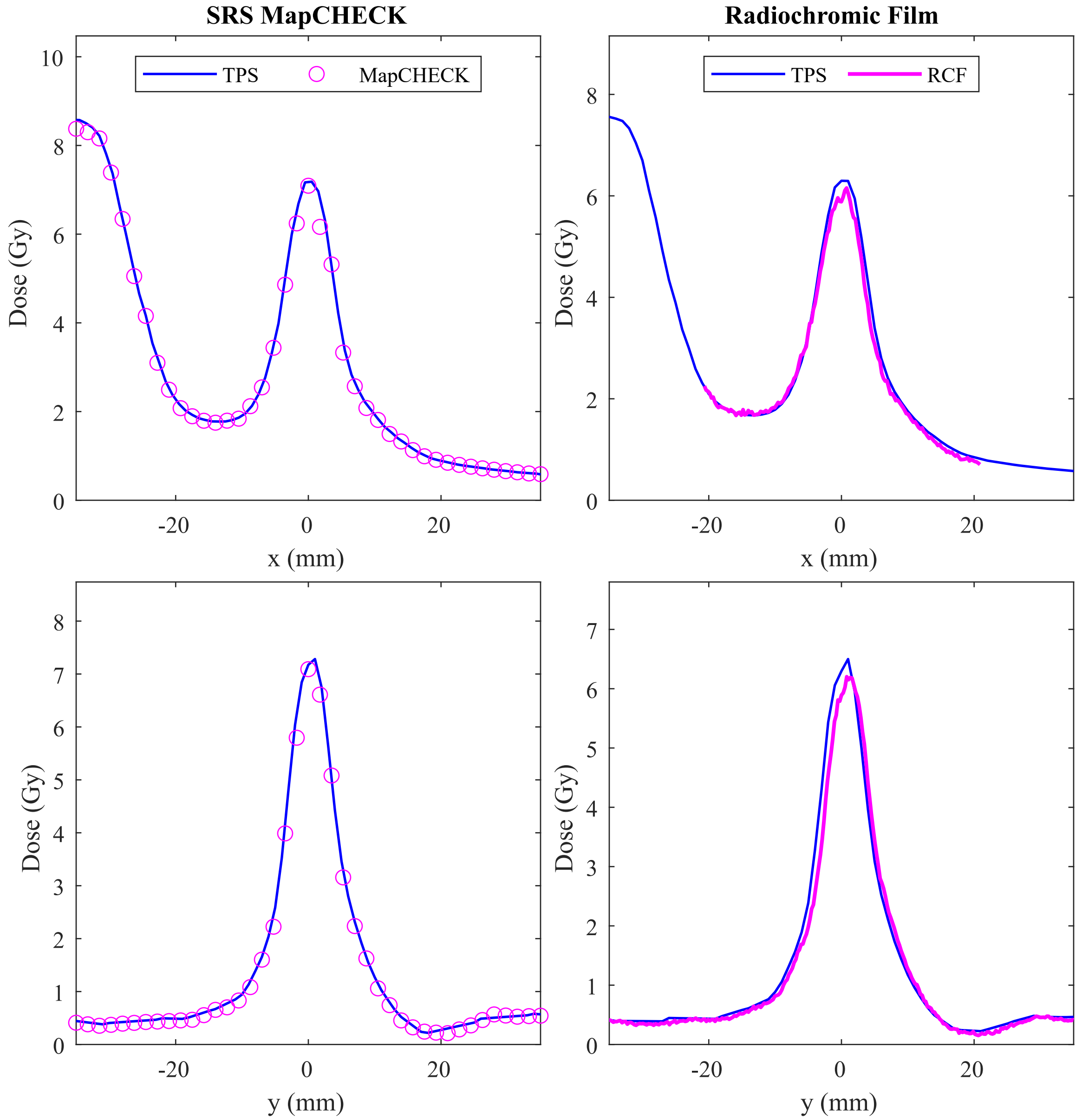

Measurement 46. Plan 30, 4 targets, equivalent diameter 19.9 mm

SRS MapCHECK fraction passing gamma 3%/1 mm = 100.0%

Radichromic film fraction passing gamma 3%/1 mm = 99.3%

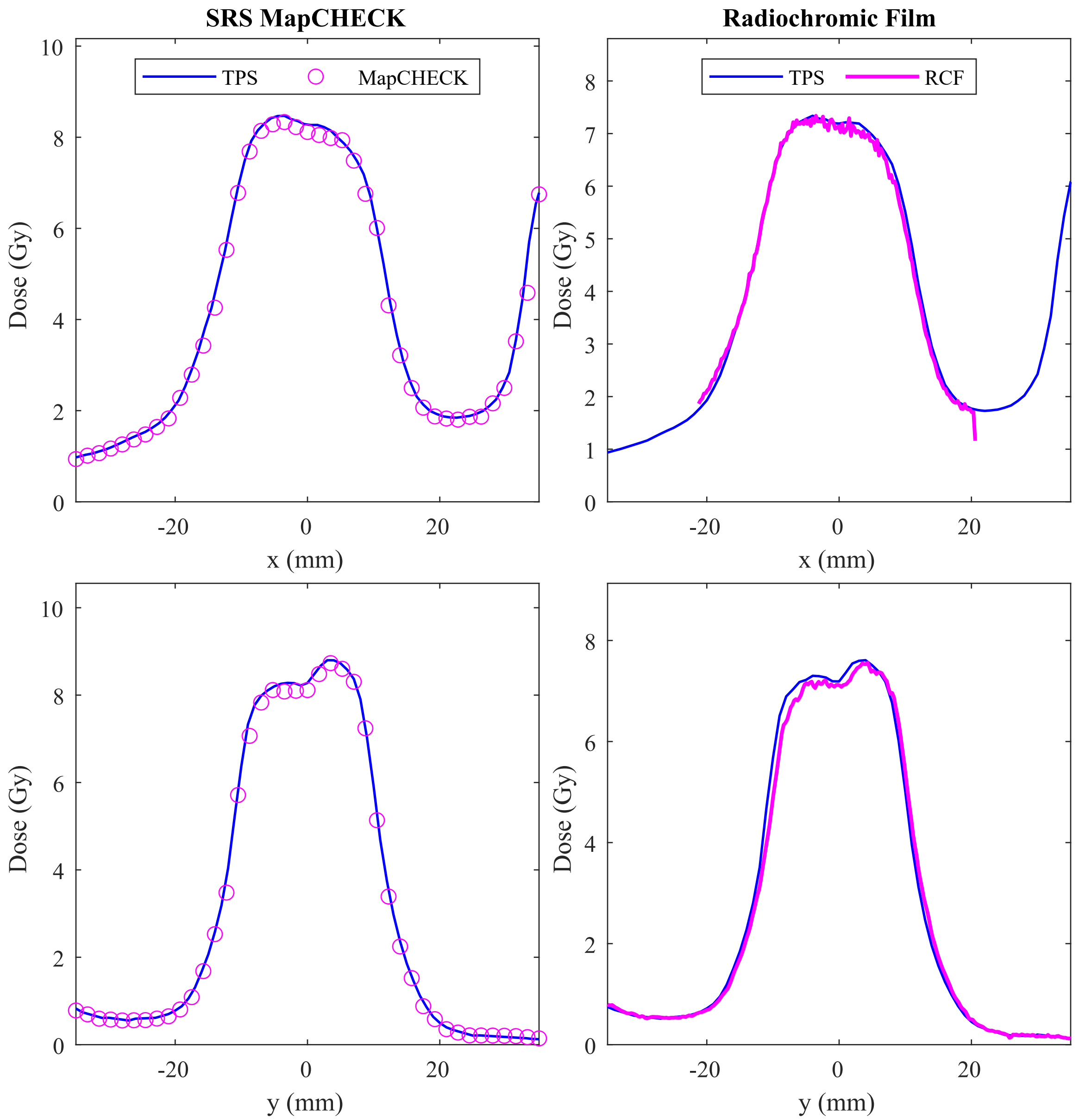

Measurement 47. Plan 31, 7 targets, equivalent diameter 3.2 mm

SRS MapCHECK fraction passing gamma 3%/1 mm = 99.2%

Radichromic film fraction passing gamma 3%/1 mm = 99.9%

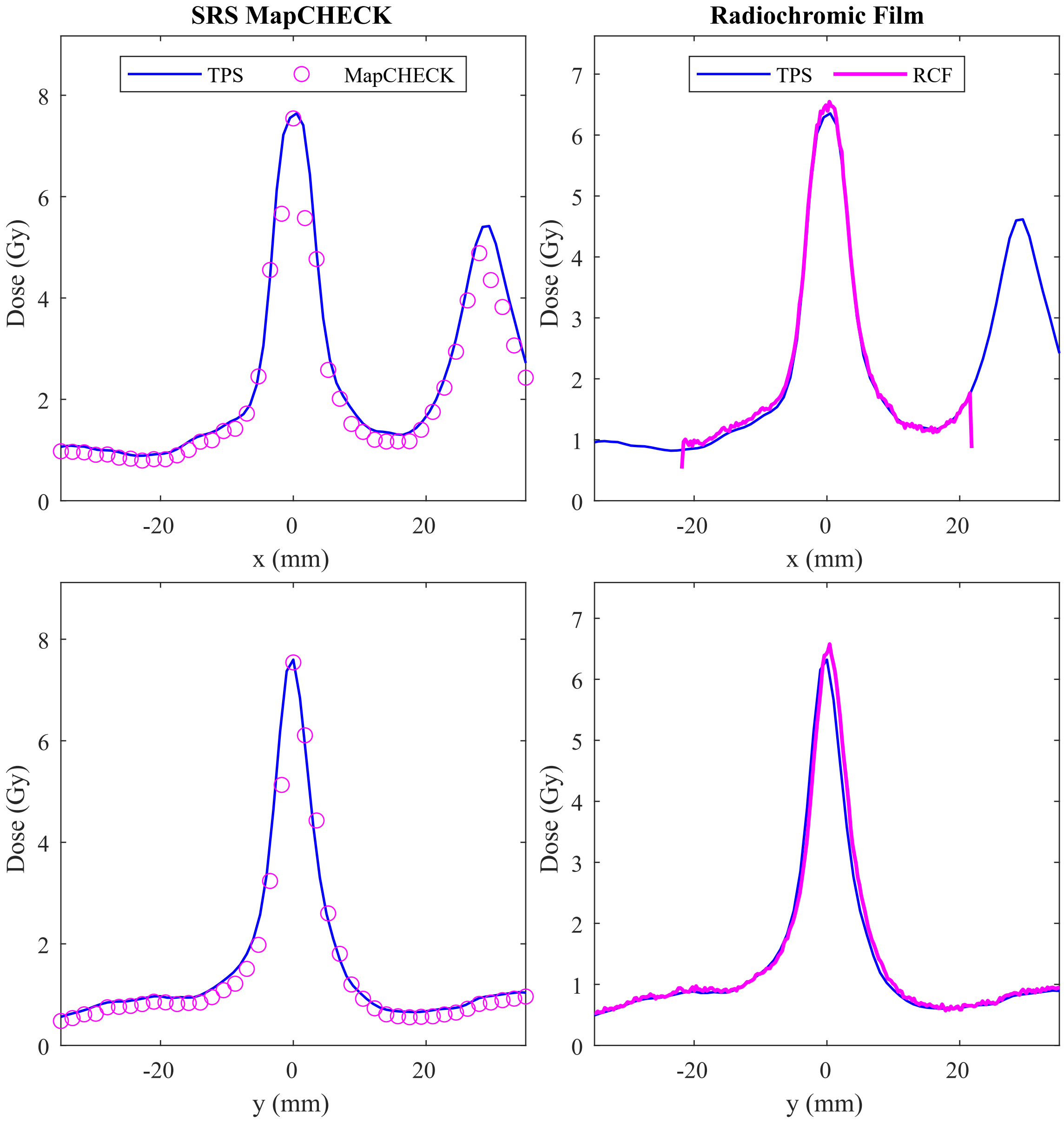

Measurement 48. Plan 31, 7 targets, equivalent diameter 20.4 mm

SRS MapCHECK fraction passing gamma 3%/1 mm = 99.0%

Radichromic film fraction passing gamma 3%/1 mm = 99.8%

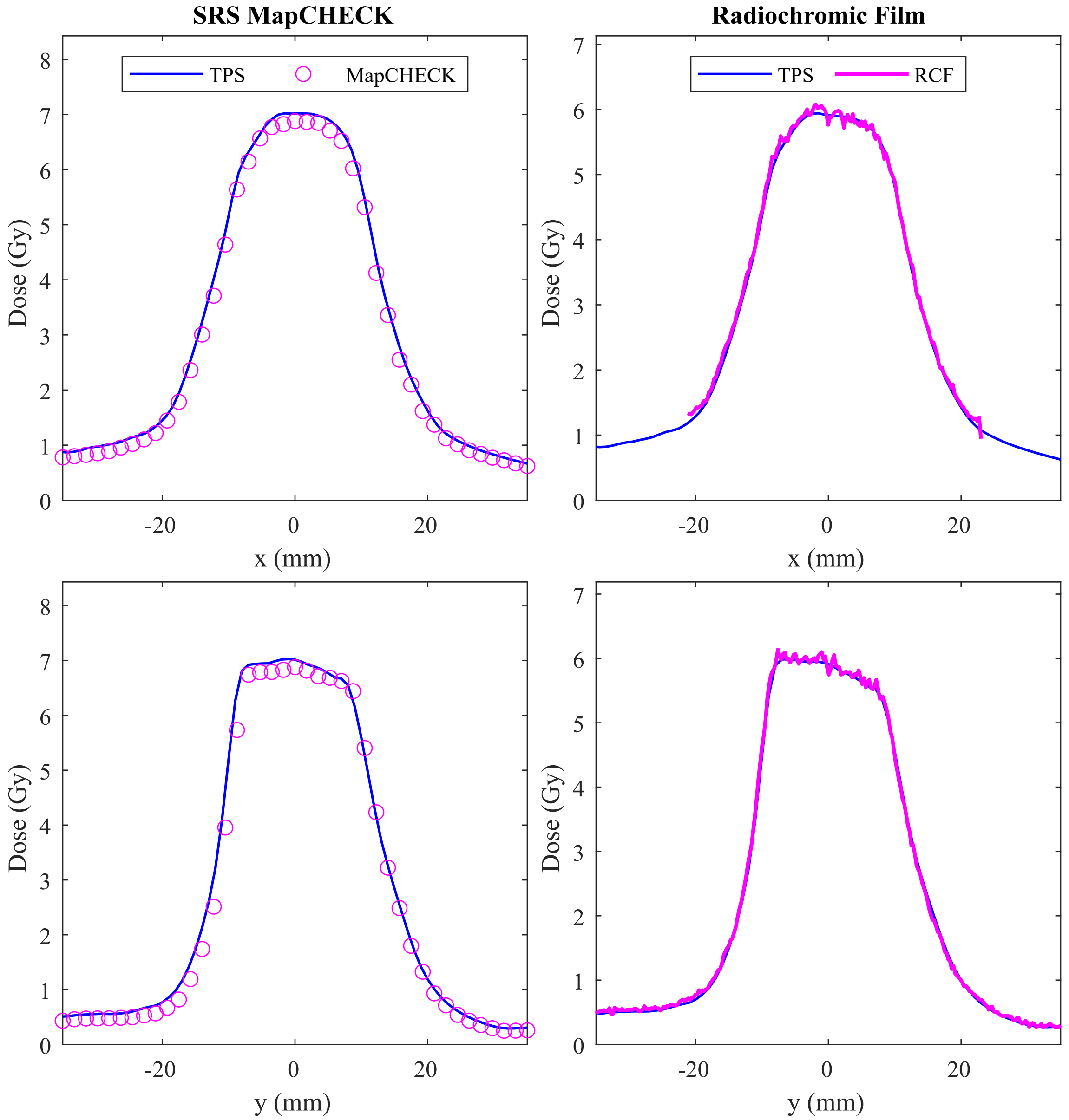

Measurement 49. Plan 32, 10 targets, equivalent diameter 2.4 mm

SRS MapCHECK fraction passing gamma 3%/1 mm = 94.4%

Radichromic film fraction passing gamma 3%/1 mm = 100.0%

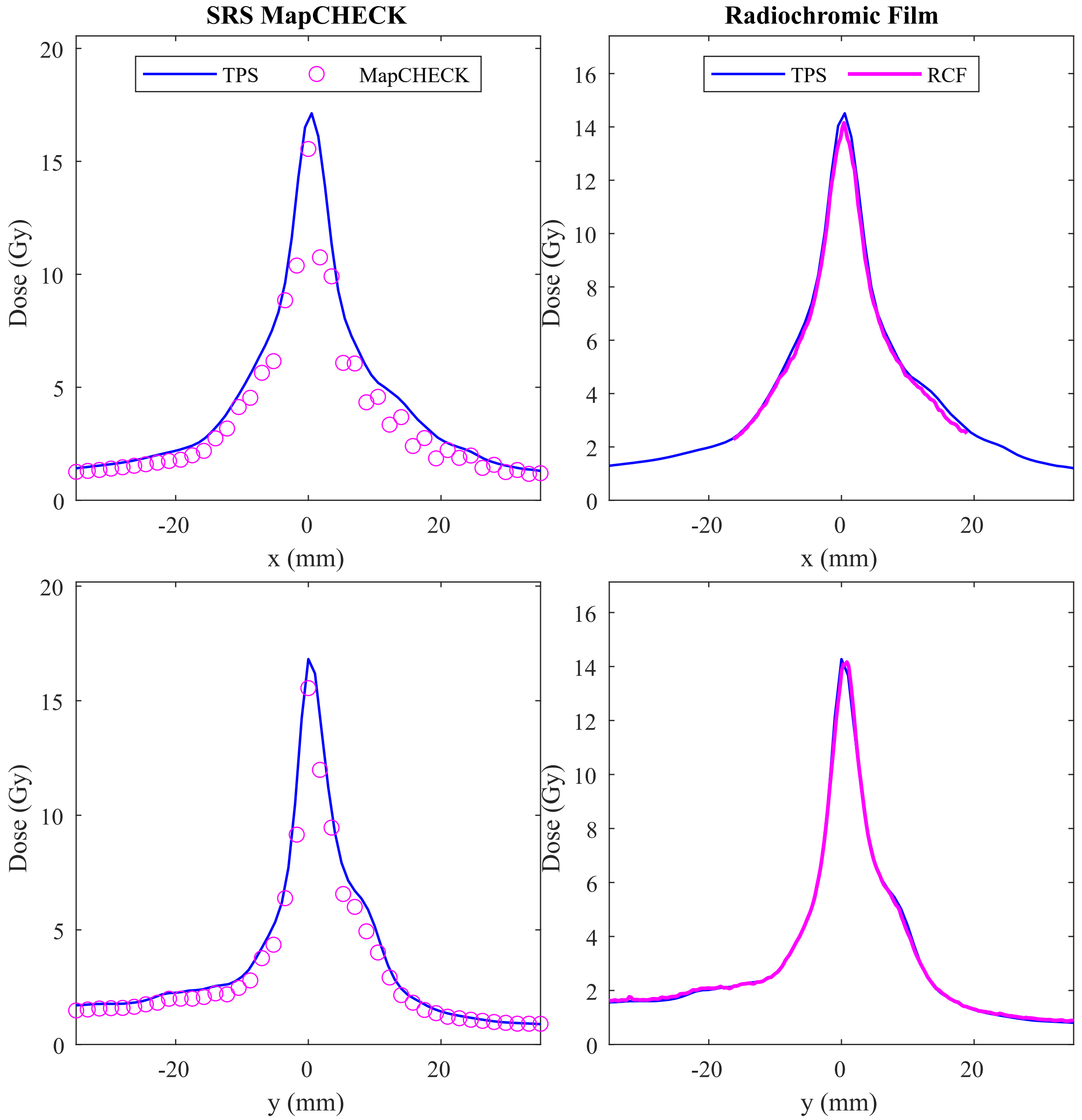

Measurement 50. Plan 32, 10 targets, equivalent diameter 3.8 mm

SRS MapCHECK fraction passing gamma 3%/1 mm = 87.9%

Radichromic film fraction passing gamma 3%/1 mm = 98.6%

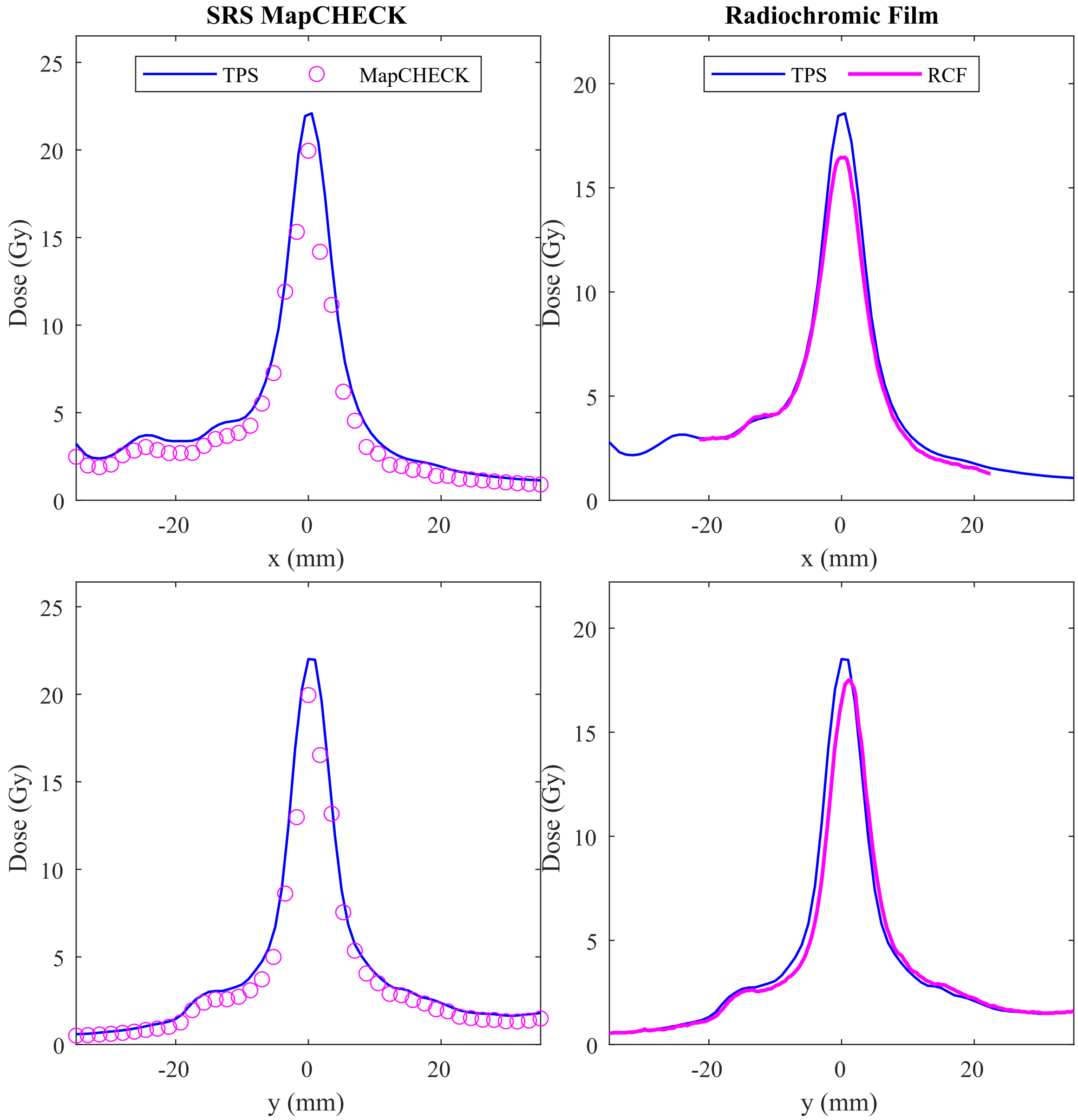

Measurement 51. Plan 33, 8 targets, equivalent diameter 8.3 mm

SRS MapCHECK fraction passing gamma 3%/1 mm = 98.7%

Radichromic film fraction passing gamma 3%/1 mm = 100.0%

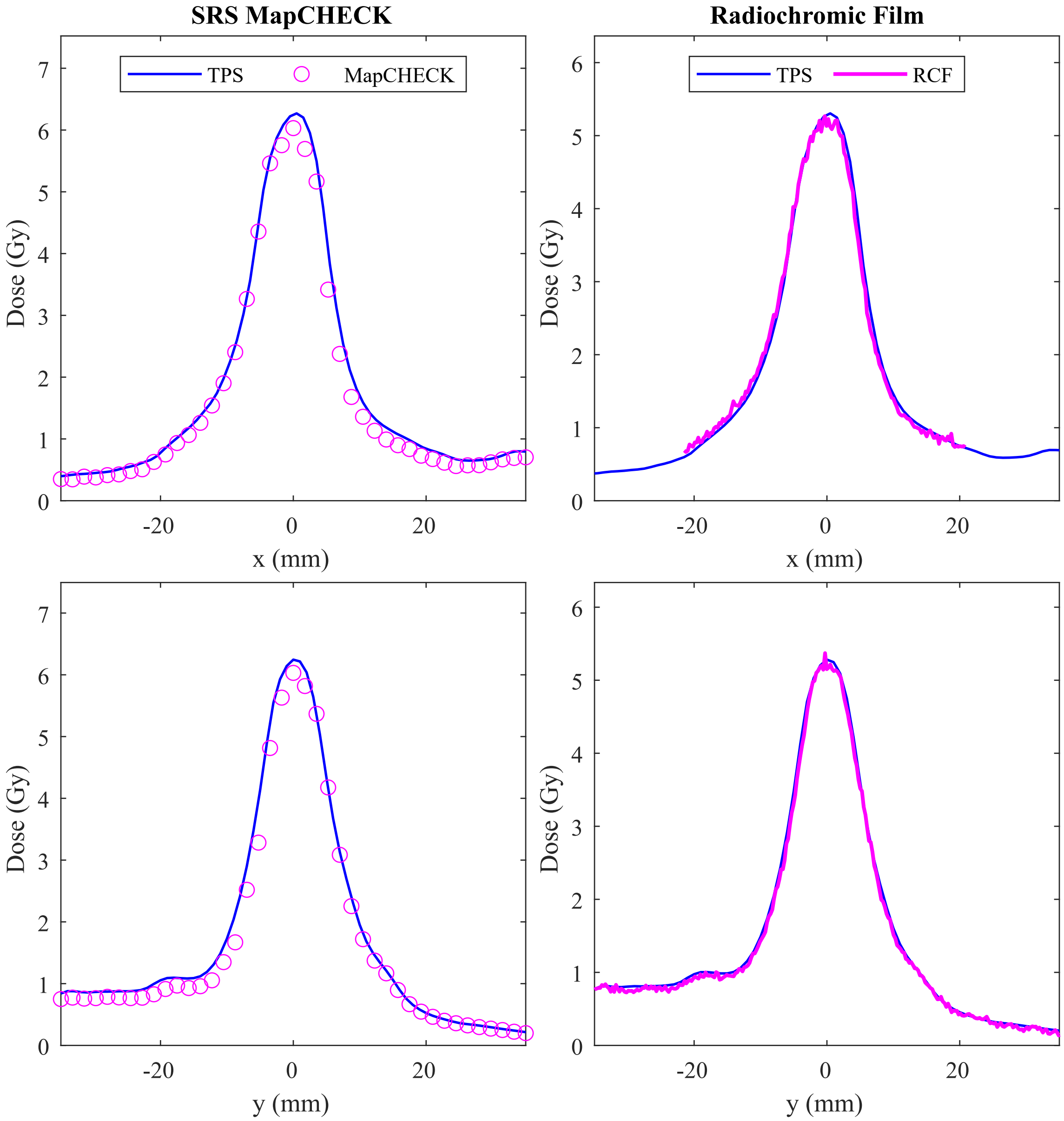

Measurement 52. Plan 33, 8 targets, equivalent diameter 15.7 mm

SRS MapCHECK fraction passing gamma 3%/1 mm = 99.8%

Radichromic film fraction passing gamma 3%/1 mm = 98.6%

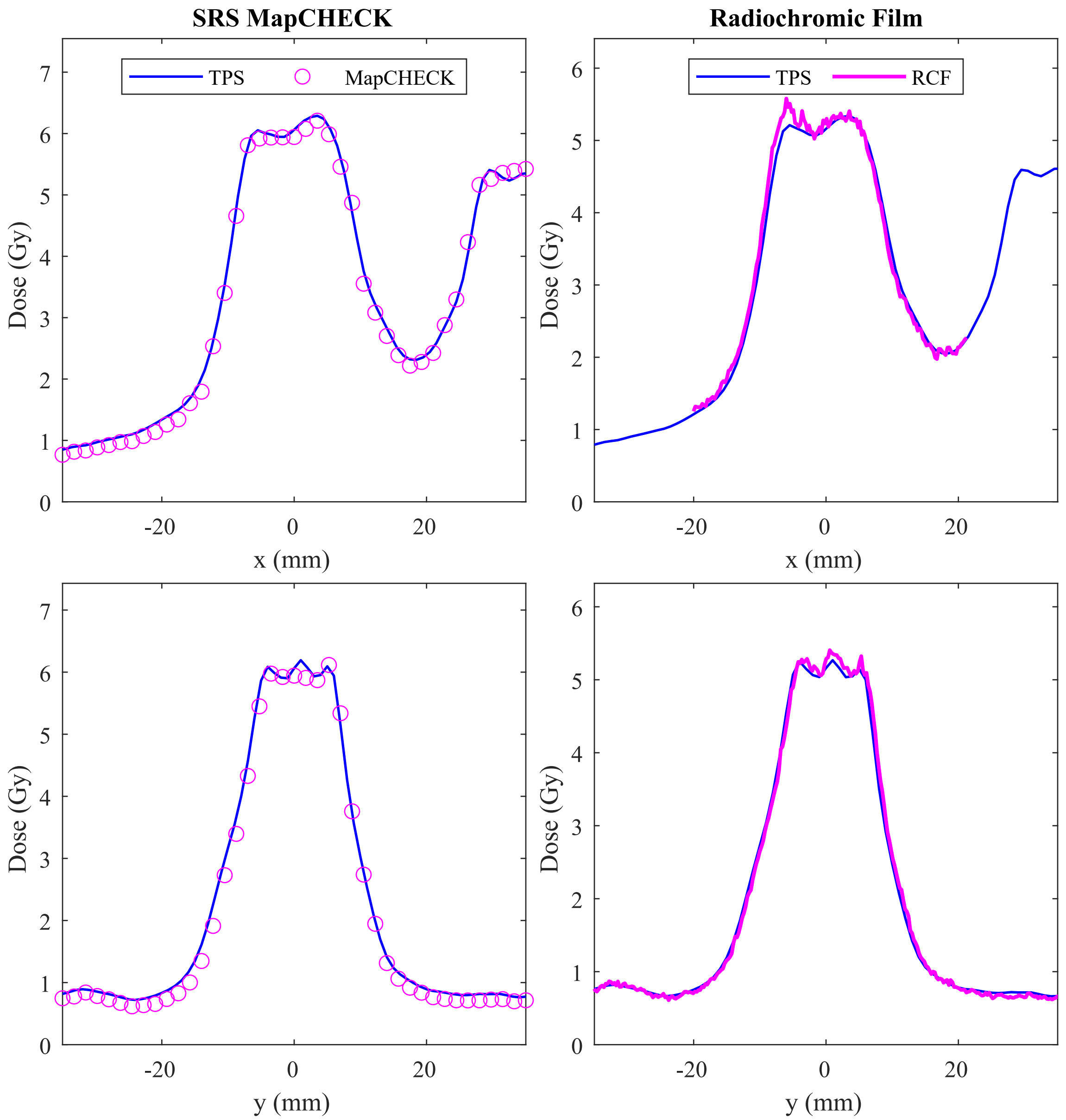

Measurement 53. Plan 34, 2 targets, equivalent diameter 10.8 mm

SRS MapCHECK fraction passing gamma 3%/1 mm = 100.0%

Radichromic film fraction passing gamma 3%/1 mm = 99.7%

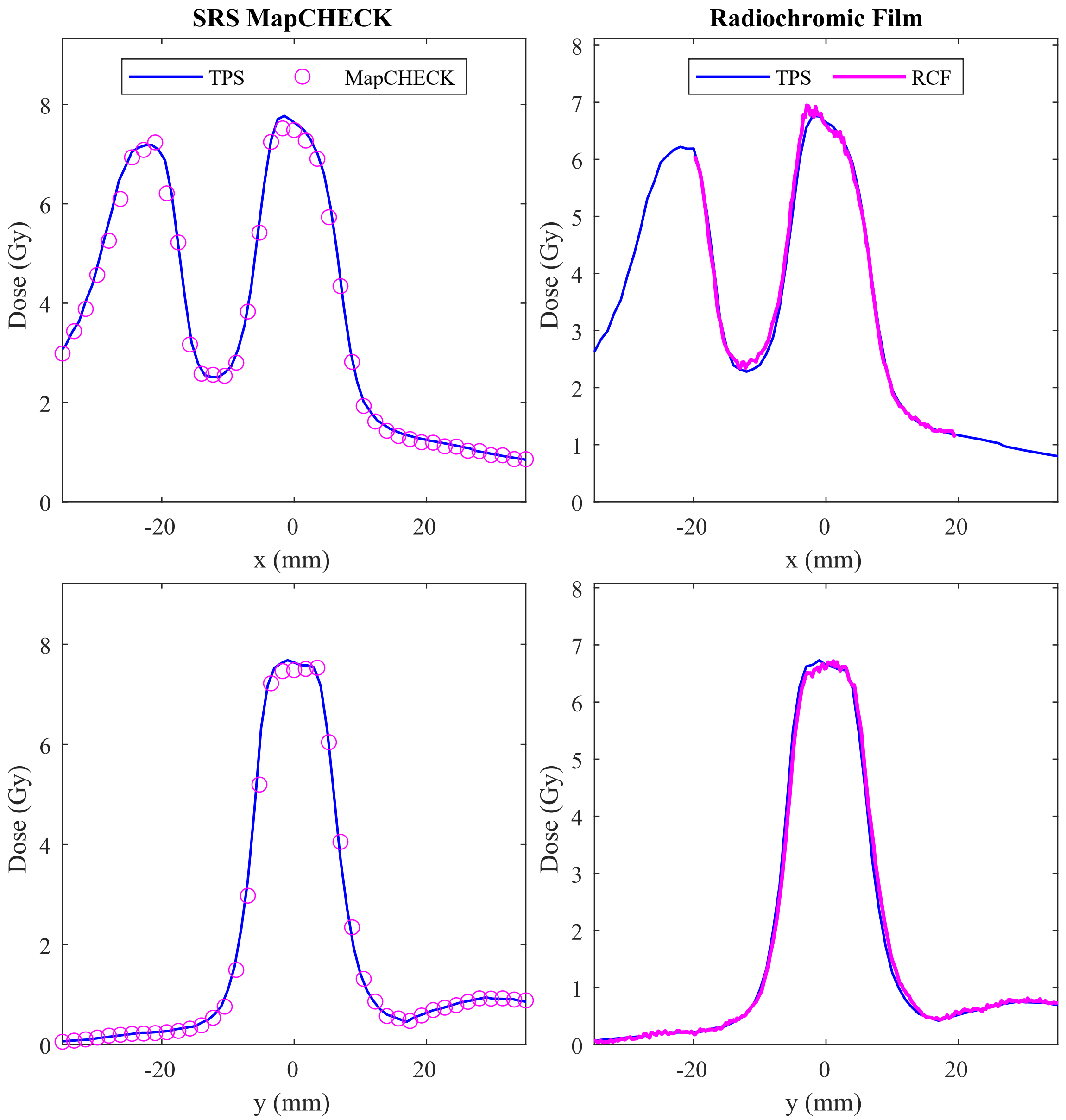

Measurement 54. Plan 34, 2 targets, equivalent diameter 15.7 mm

SRS MapCHECK fraction passing gamma 3%/1 mm = 99.7%

Radichromic film fraction passing gamma 3%/1 mm = 98.6%

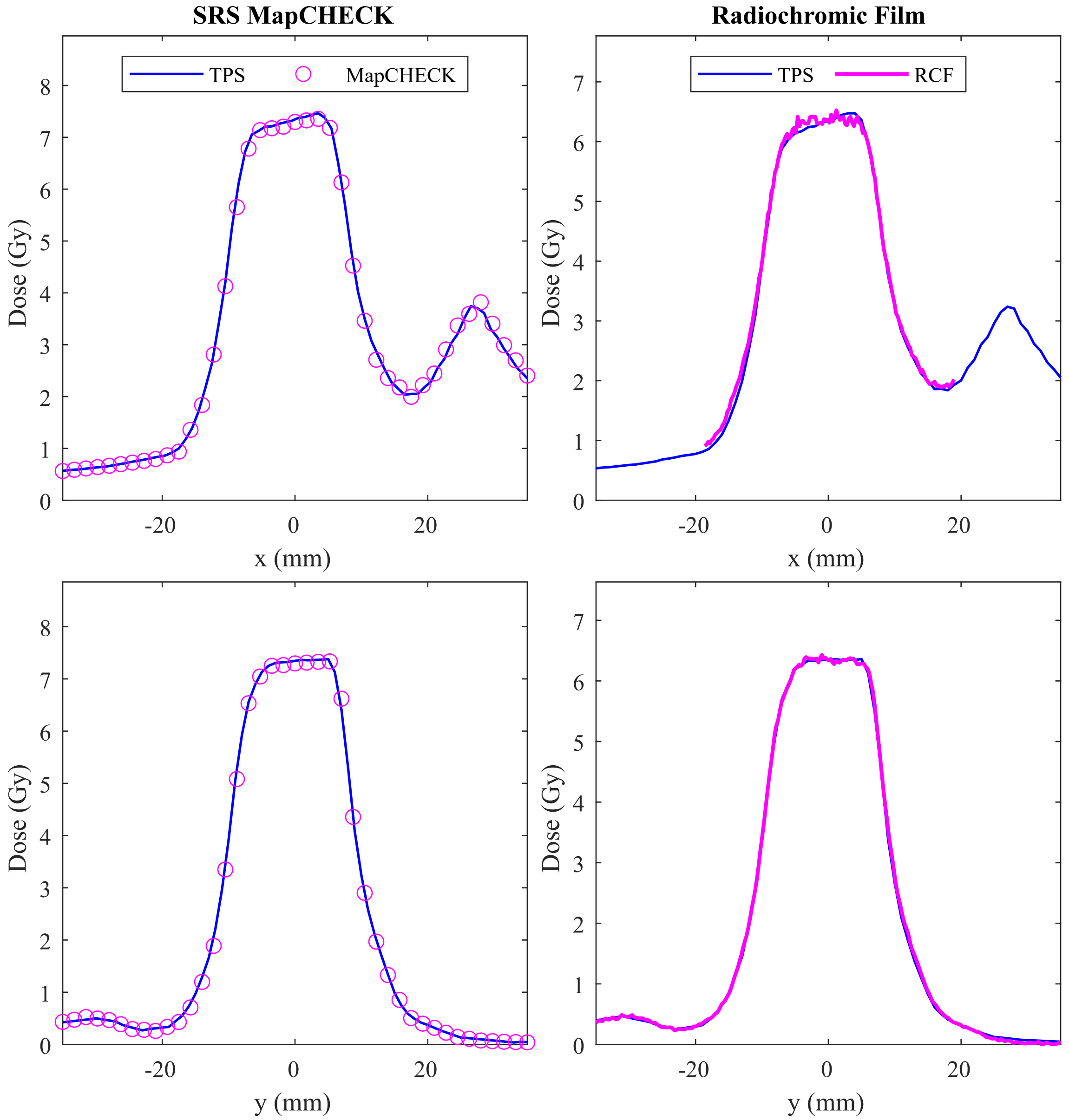

Measurement 55. Plan 35, single target, equivalent diameter 17.0 mm

SRS MapCHECK fraction passing gamma 3%/1 mm = 99.6%

Radichromic film fraction passing gamma 3%/1 mm = 99.4%

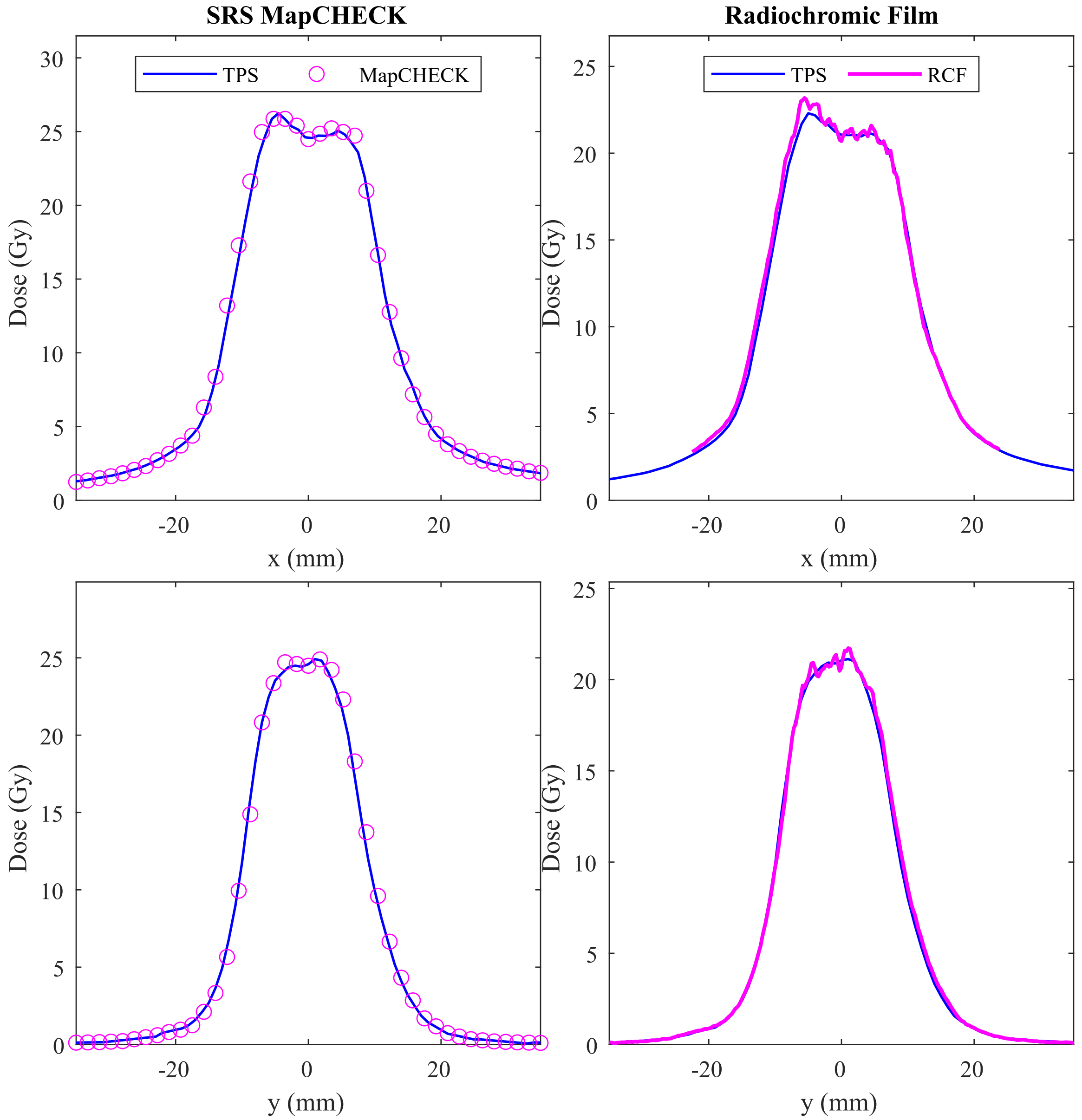

Measurement 56. Plan 36, single target, equivalent diameter 20.0 mm

SRS MapCHECK fraction passing gamma 3%/1 mm = 98.6%

Radichromic film fraction passing gamma 3%/1 mm = 92.6%

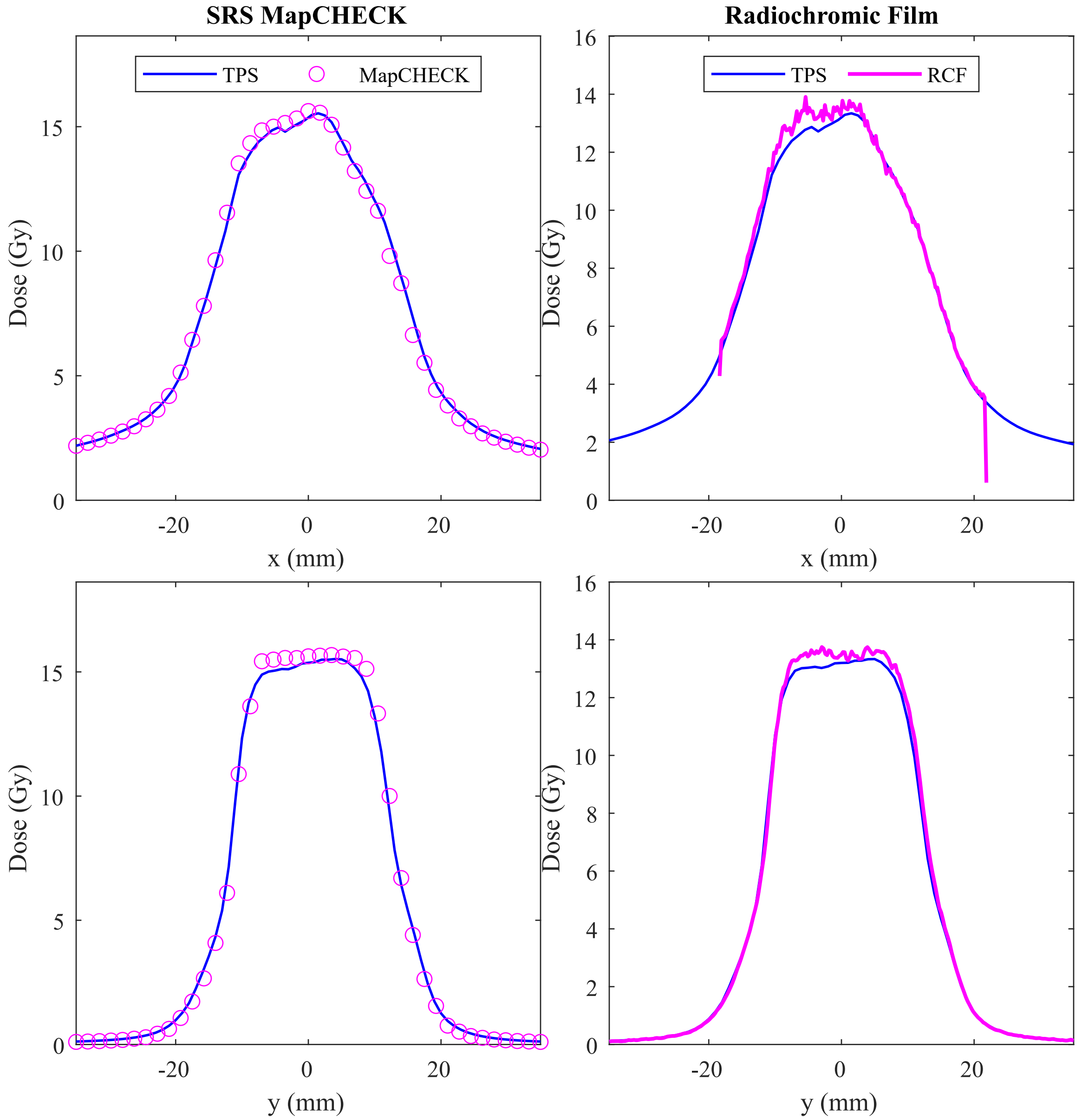

Measurement 57. Plan 37, 2 targets, equivalent diameter 3.8 mm

SRS MapCHECK fraction passing gamma 3%/1 mm = 100.0%

Radichromic film fraction passing gamma 3%/1 mm = 100.0%

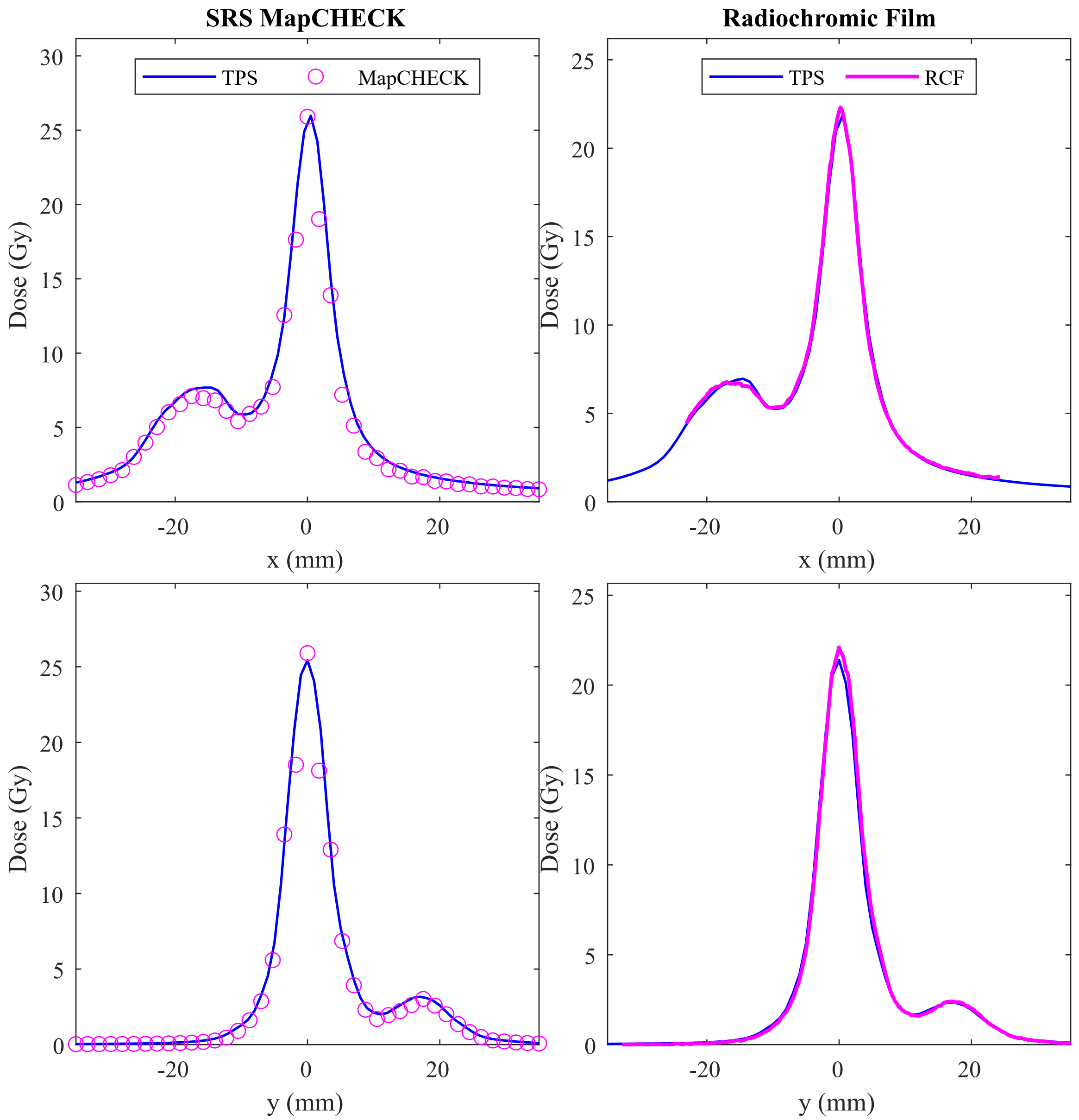

Measurement 58. Plan 37, 2 targets, equivalent diameter 6.6 mm

SRS MapCHECK fraction passing gamma 3%/1 mm = 100.0%

Radichromic film fraction passing gamma 3%/1 mm = 100.0%

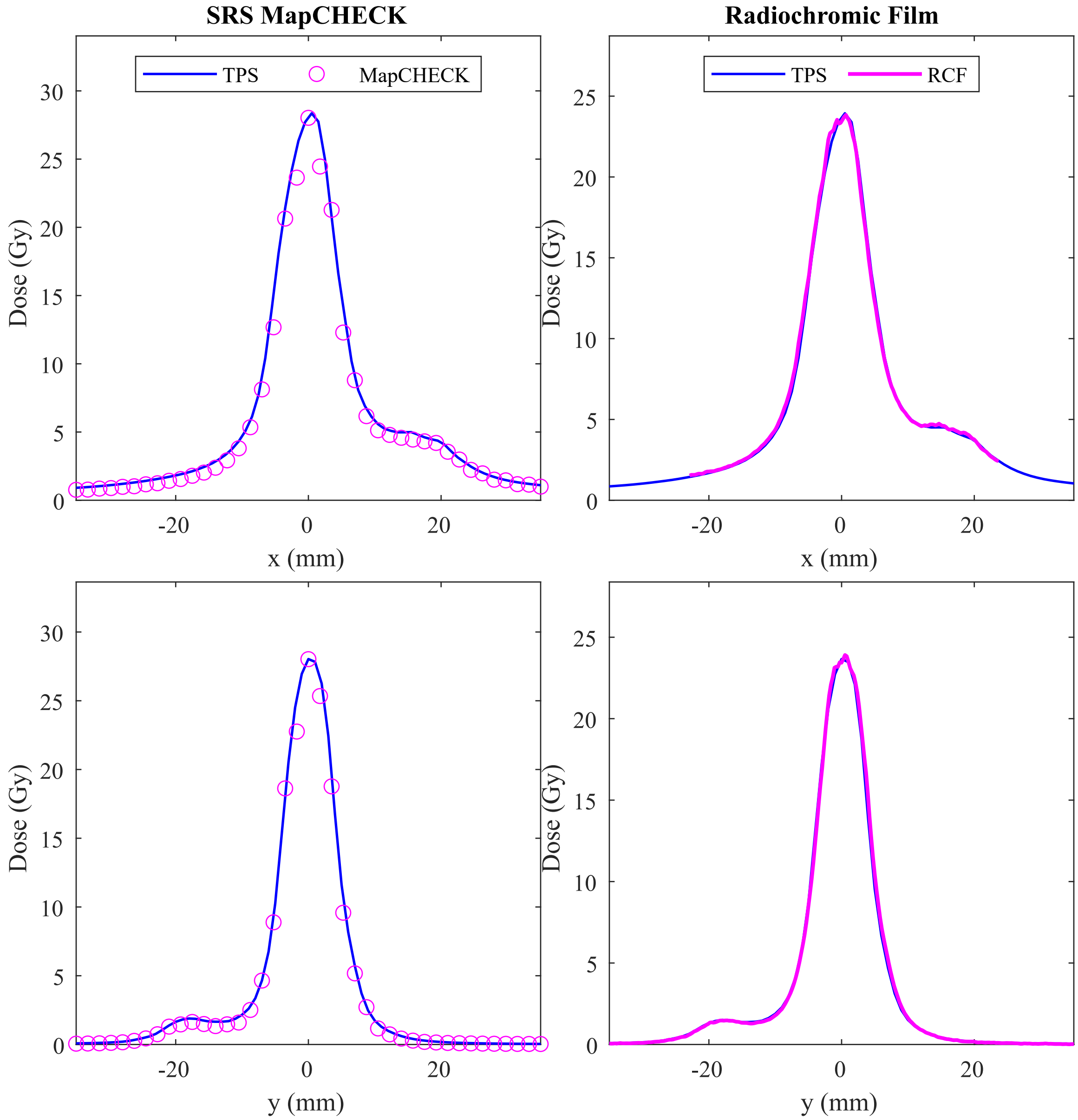

Measurement 59. Plan 38, 2 targets, equivalent diameter 4.6 mm

SRS MapCHECK fraction passing gamma 3%/1 mm = 100.0%

Radichromic film fraction passing gamma 3%/1 mm = 93.1%

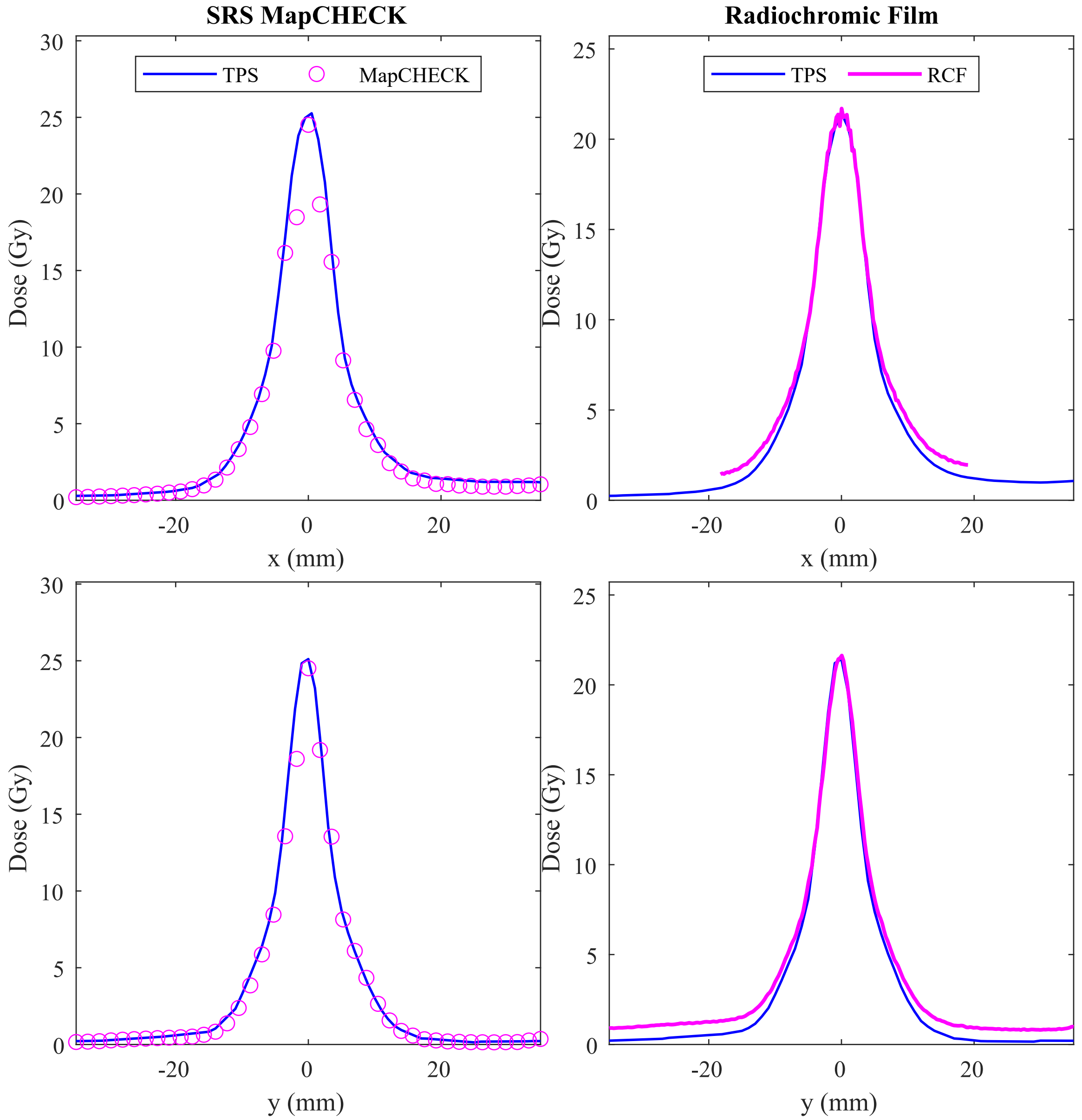

Measurement 60. Plan 38, 2 targets, equivalent diameter 5.9 mm

SRS MapCHECK fraction passing gamma 3%/1 mm = 98.8%

Radichromic film fraction passing gamma 3%/1 mm = 99.9%

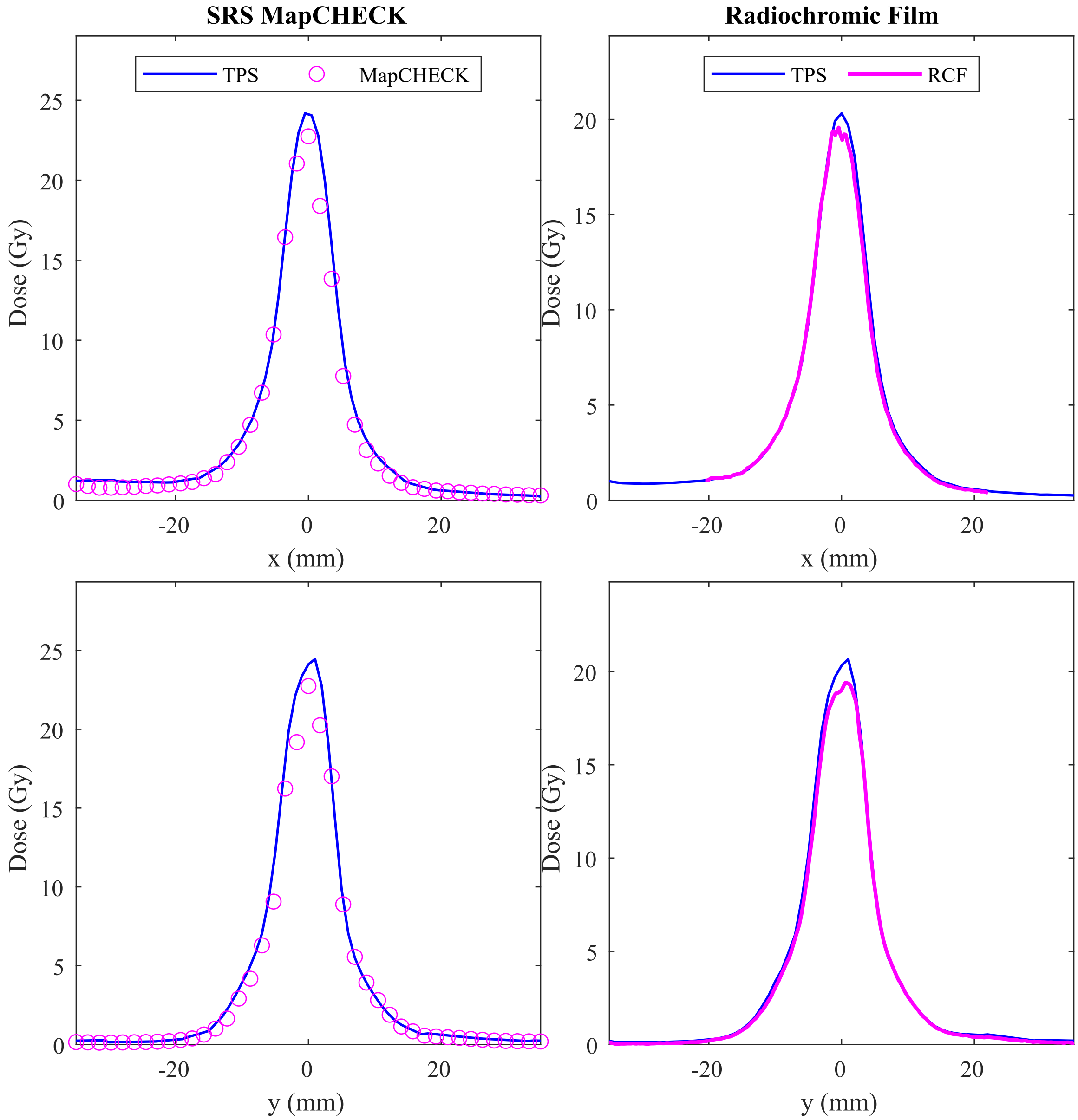

Measurement 61. Plan 39, single target, equivalent diameter 5.9 mm

SRS MapCHECK fraction passing gamma 3%/1 mm = 100.0%

Radichromic film fraction passing gamma 3%/1 mm = 100.0%

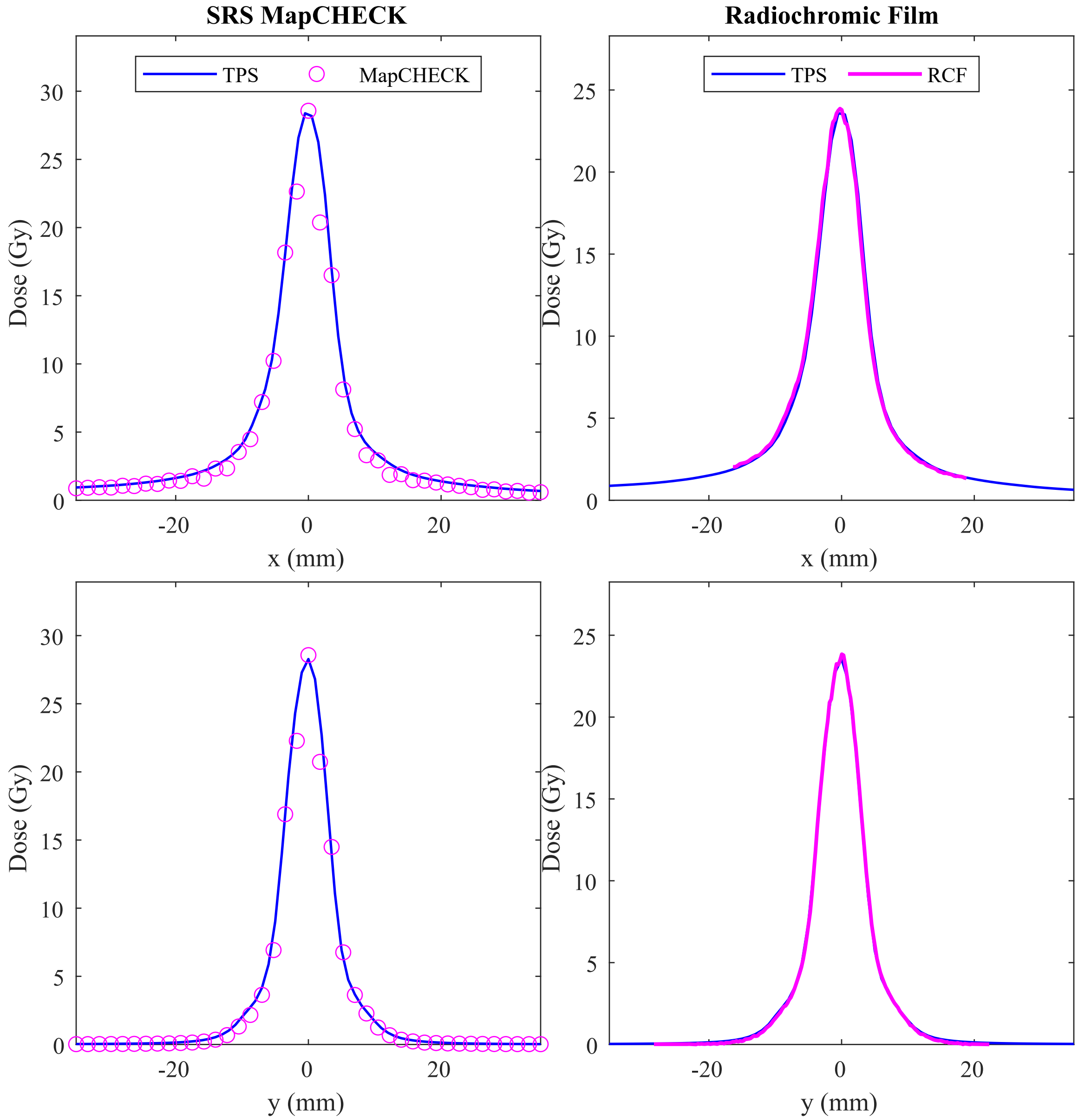

Measurement 62. Plan 40, 4 targets, equivalent diameter 8.3 mm

SRS MapCHECK fraction passing gamma 3%/1 mm = 99.4%

Radichromic film fraction passing gamma 3%/1 mm = 100.0%

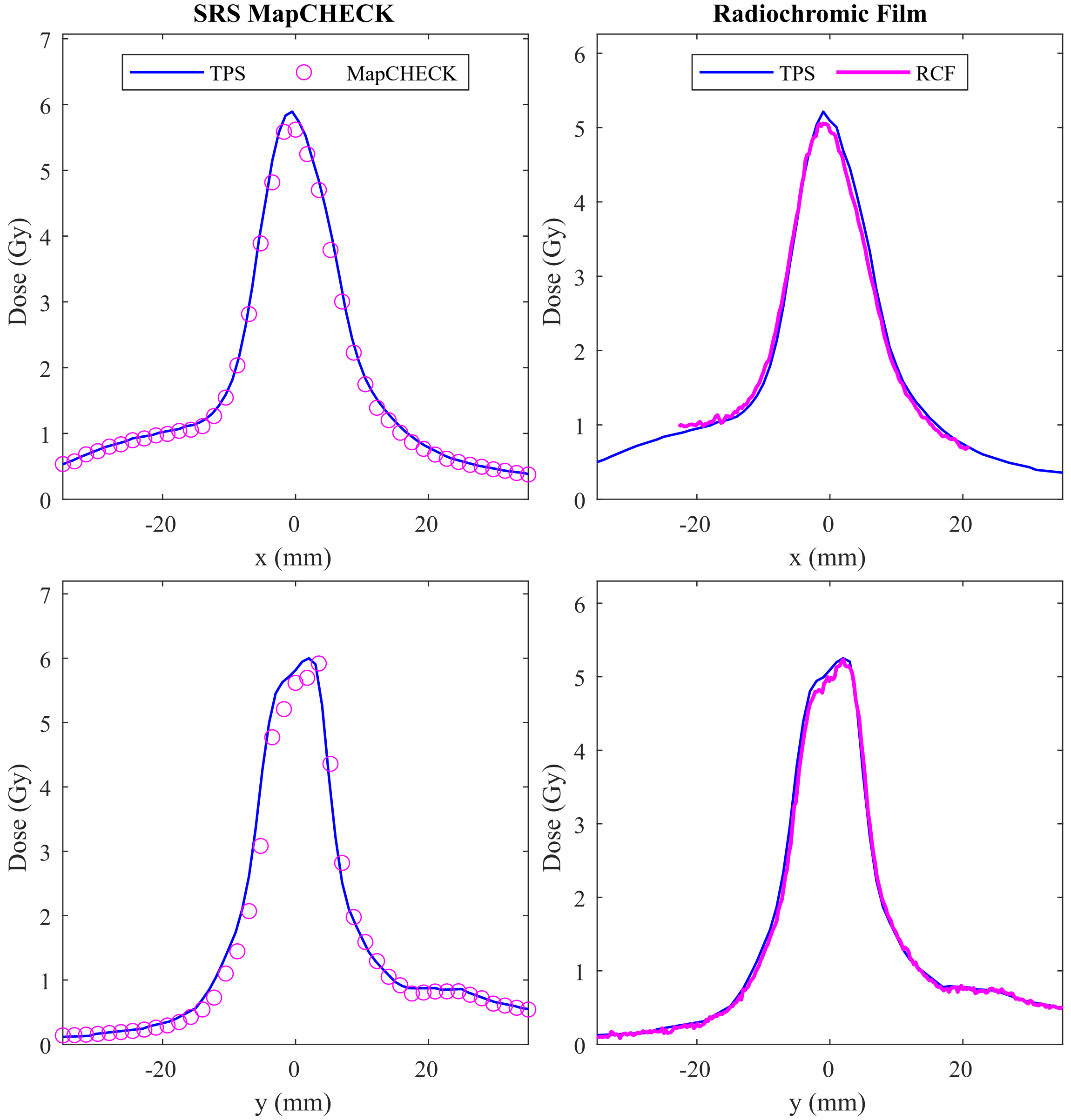

Measurement 63. Plan 40, 4 targets, equivalent diameter 11.8 mm

SRS MapCHECK fraction passing gamma 3%/1 mm = 100.0%

Radichromic film fraction passing gamma 3%/1 mm = 100.0%

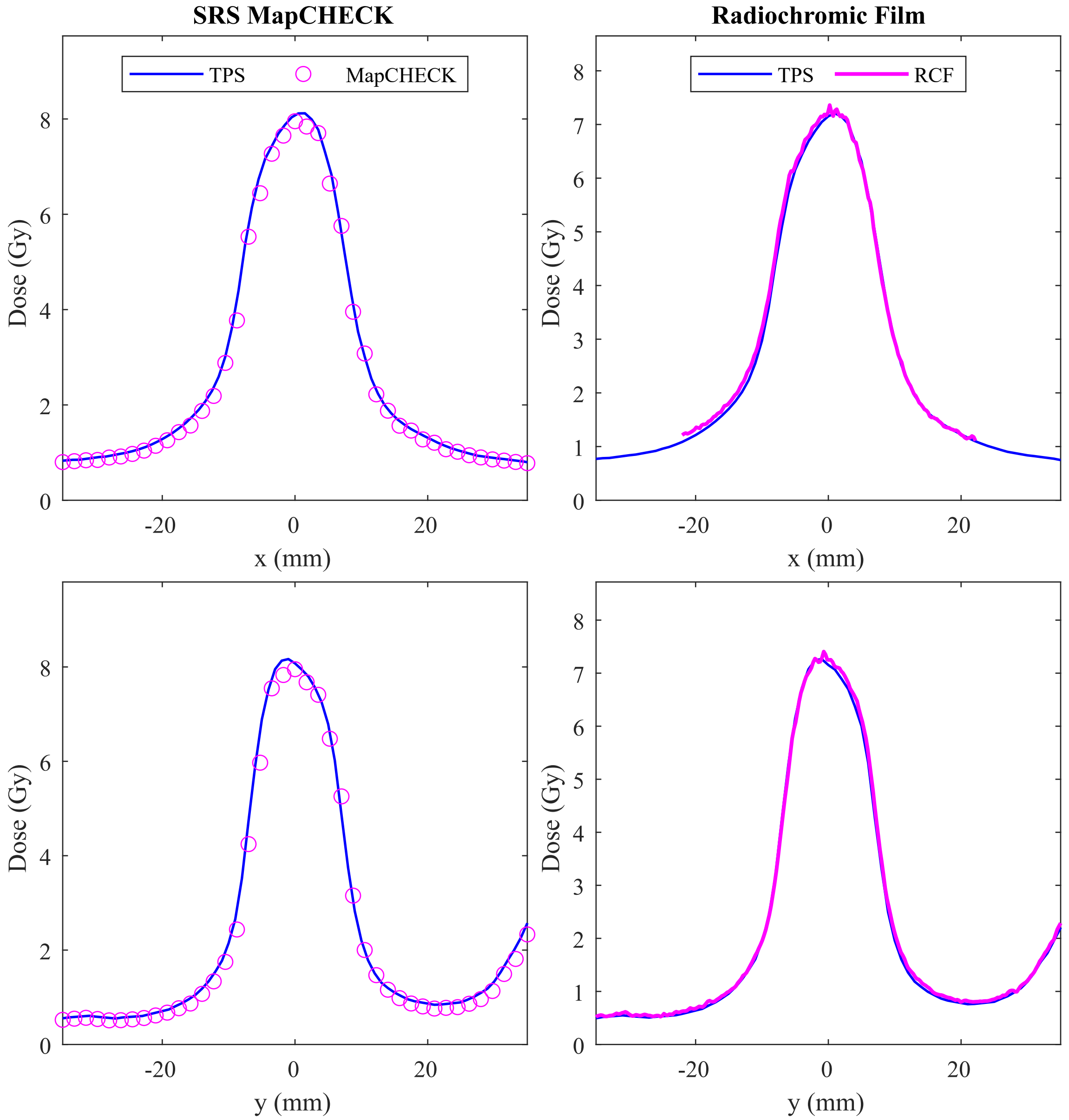

Measurement 64. Plan 41, 2 targets, equivalent diameter 3.3 mm

SRS MapCHECK fraction passing gamma 3%/1 mm = 100.0%

Radichromic film fraction passing gamma 3%/1 mm = 100.0%

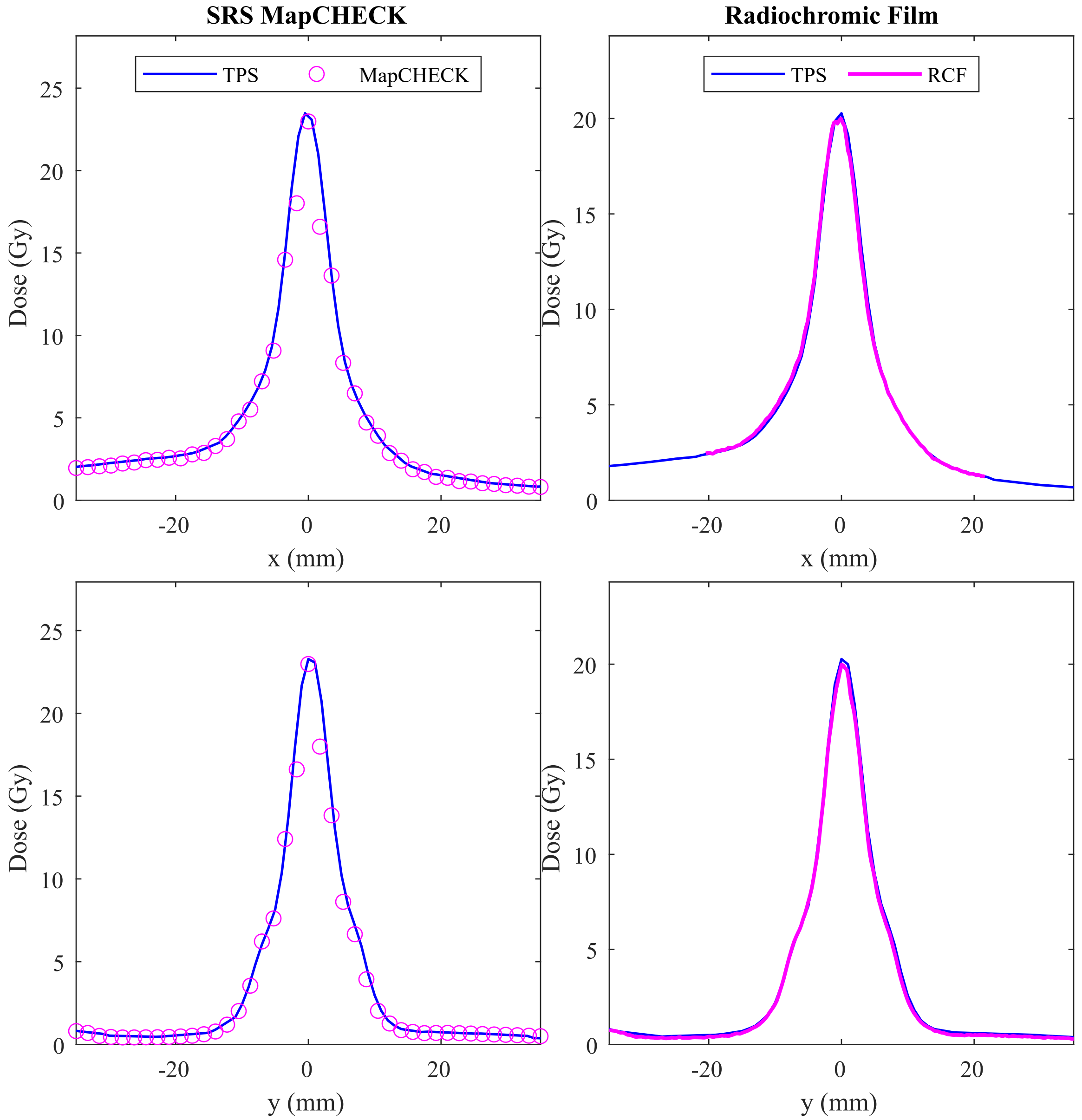

Measurement 65. Plan 41, 2 targets, equivalent diameter 26.7 mm

SRS MapCHECK fraction passing gamma 3%/1 mm = 100.0%

Radichromic film fraction passing gamma 3%/1 mm = 93.0%

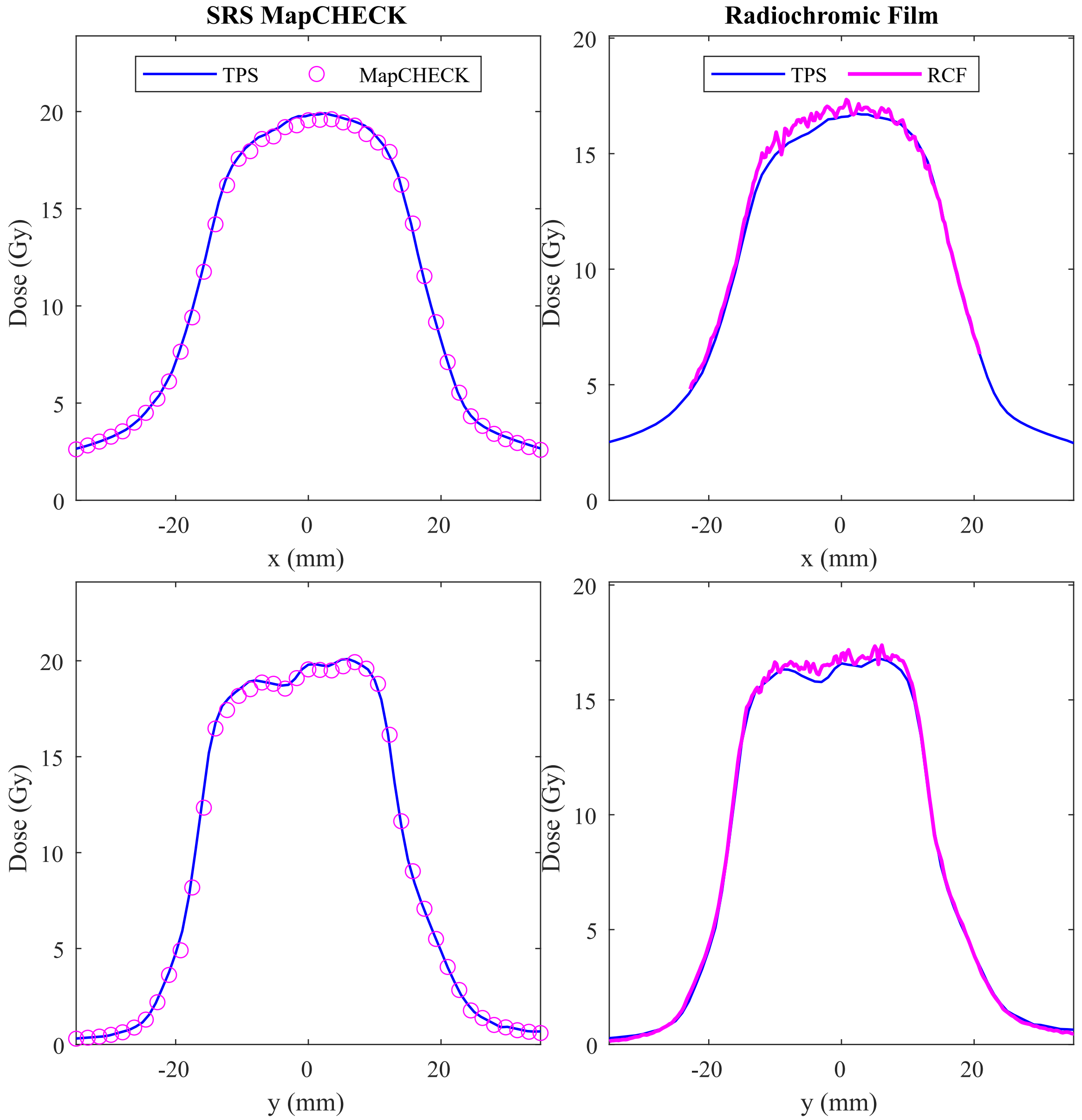

Measurement 66. Plan 42, 2 targets, equivalent diameter 3.4 mm

SRS MapCHECK fraction passing gamma 3%/1 mm = 100.0%

Radichromic film fraction passing gamma 3%/1 mm = 100.0%

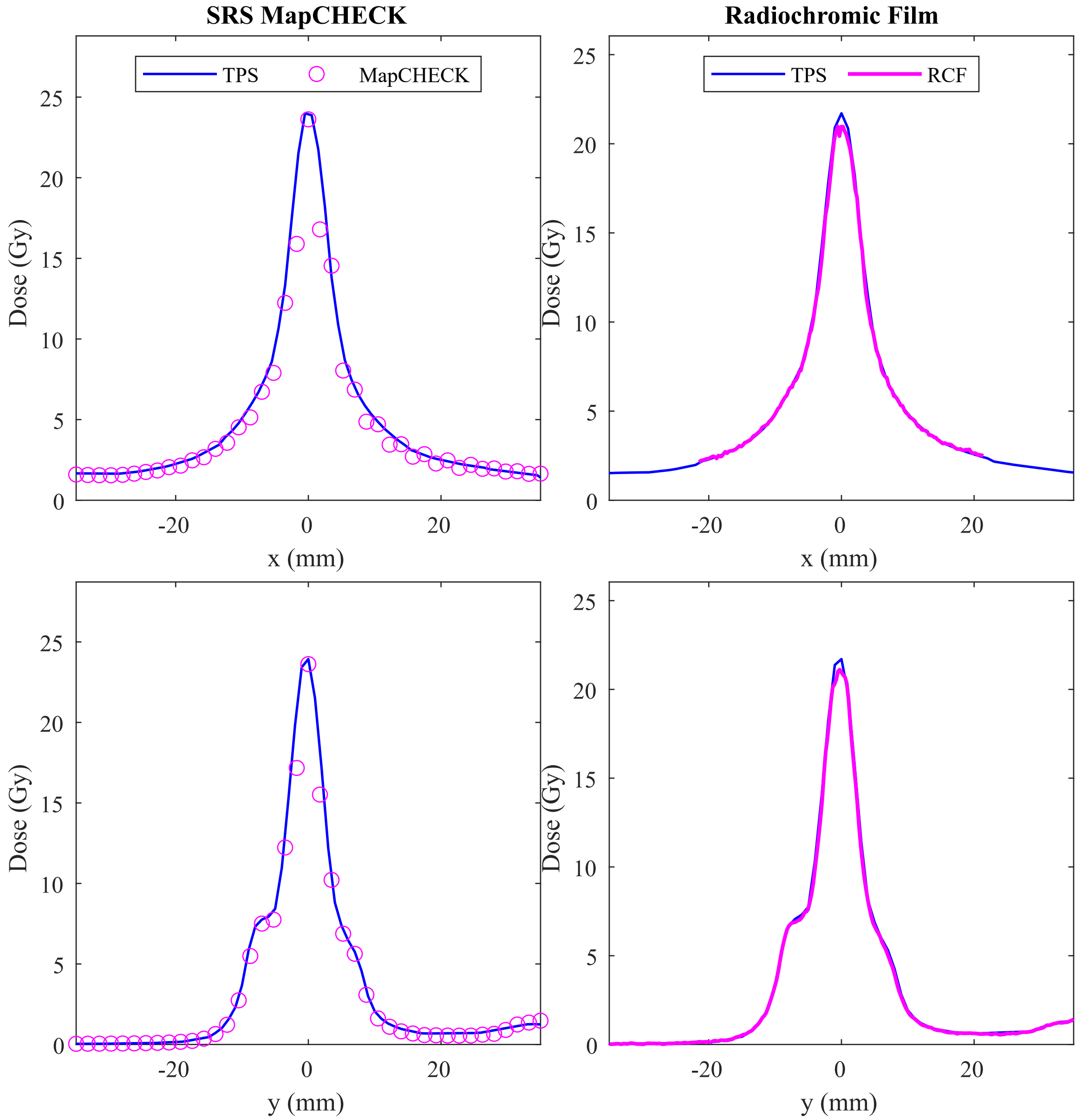

Measurement 67. Plan 42, 2 targets, equivalent diameter 10.8 mm

SRS MapCHECK fraction passing gamma 3%/1 mm = 100.0%

Radichromic film fraction passing gamma 3%/1 mm = 99.5%

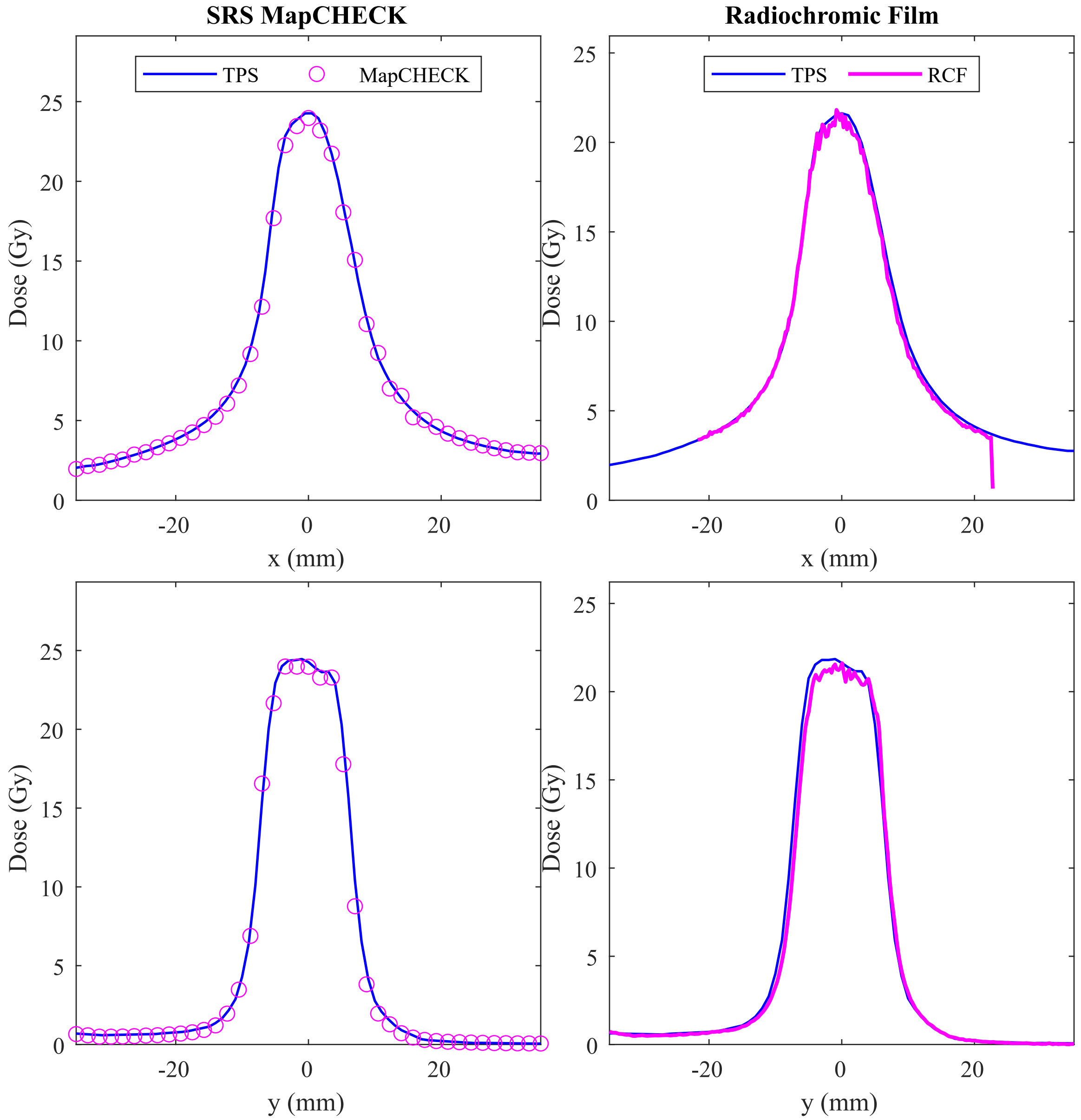

Measurement 68. Plan 43, 2 targets, equivalent diameter 2.4 mm

SRS MapCHECK fraction passing gamma 3%/1 mm = 100.0%

Radichromic film fraction passing gamma 3%/1 mm = 100.0%

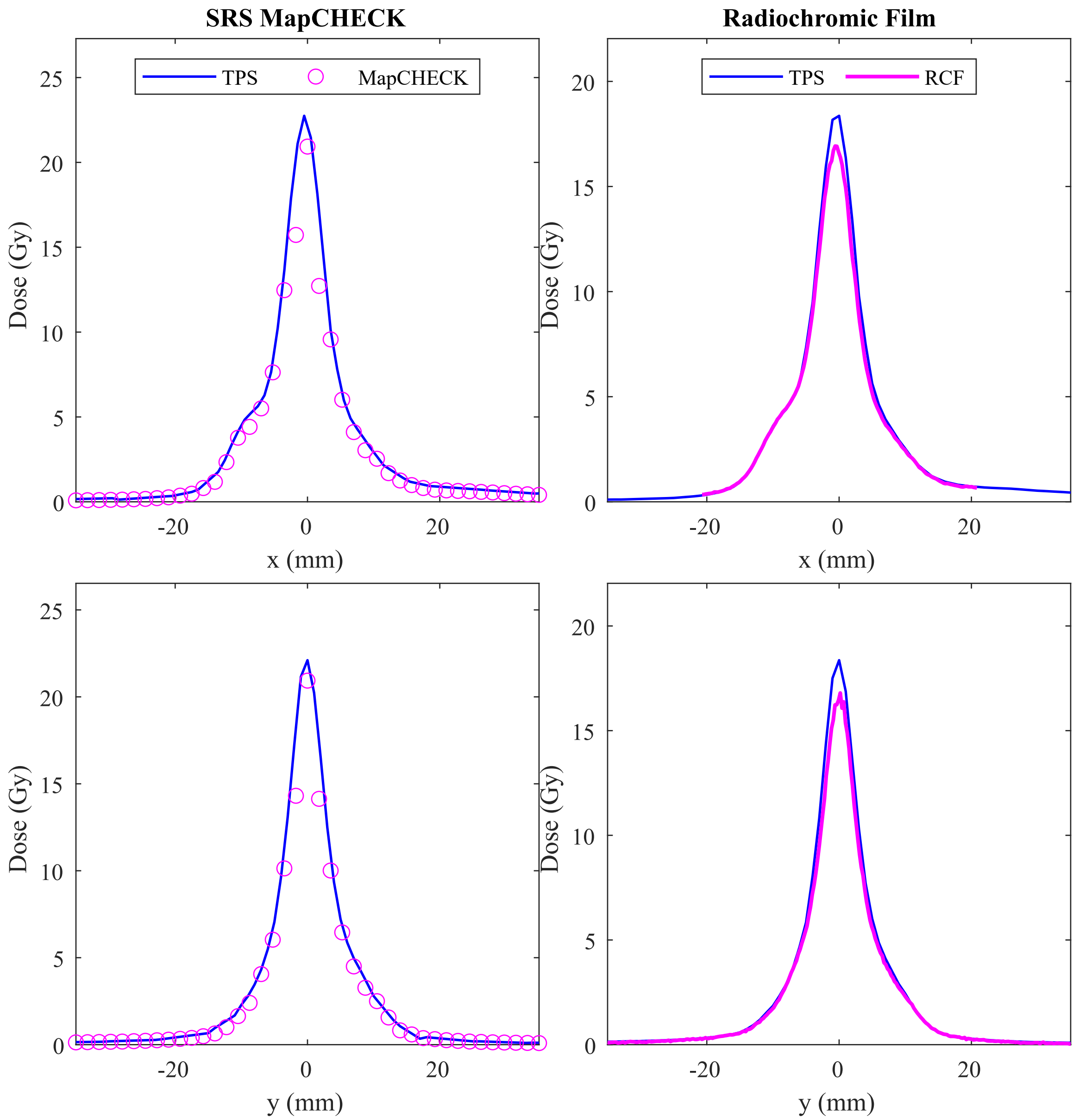

Measurement 69. Plan 43, 2 targets, equivalent diameter 5.5 mm

SRS MapCHECK fraction passing gamma 3%/1 mm = 100.0%

Radichromic film fraction passing gamma 3%/1 mm = 99.6%

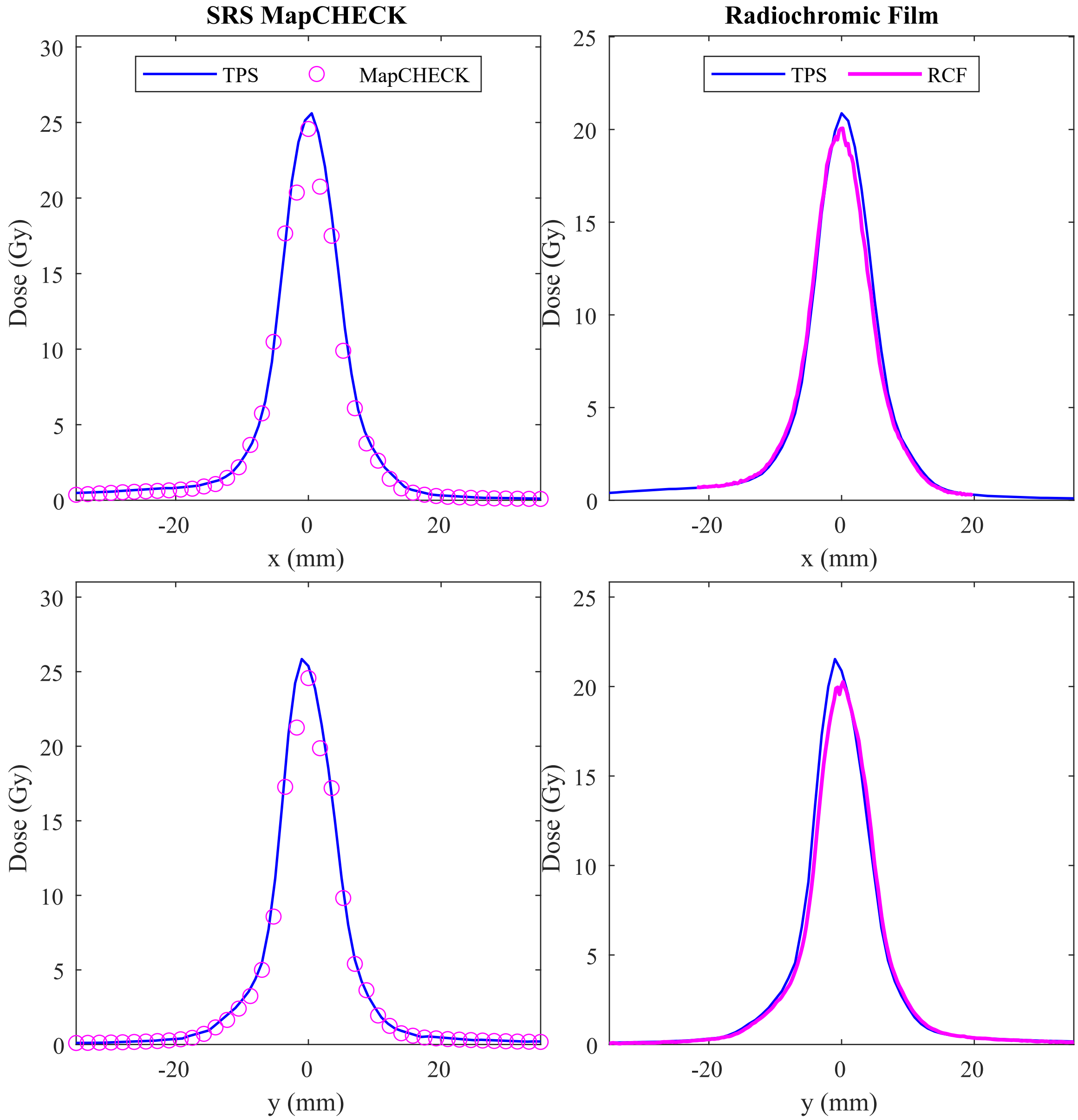

Measurement 70. Plan 44, 2 targets, equivalent diameter 5.6 mm

SRS MapCHECK fraction passing gamma 3%/1 mm = 100.0%

Radichromic film fraction passing gamma 3%/1 mm = 100.0%

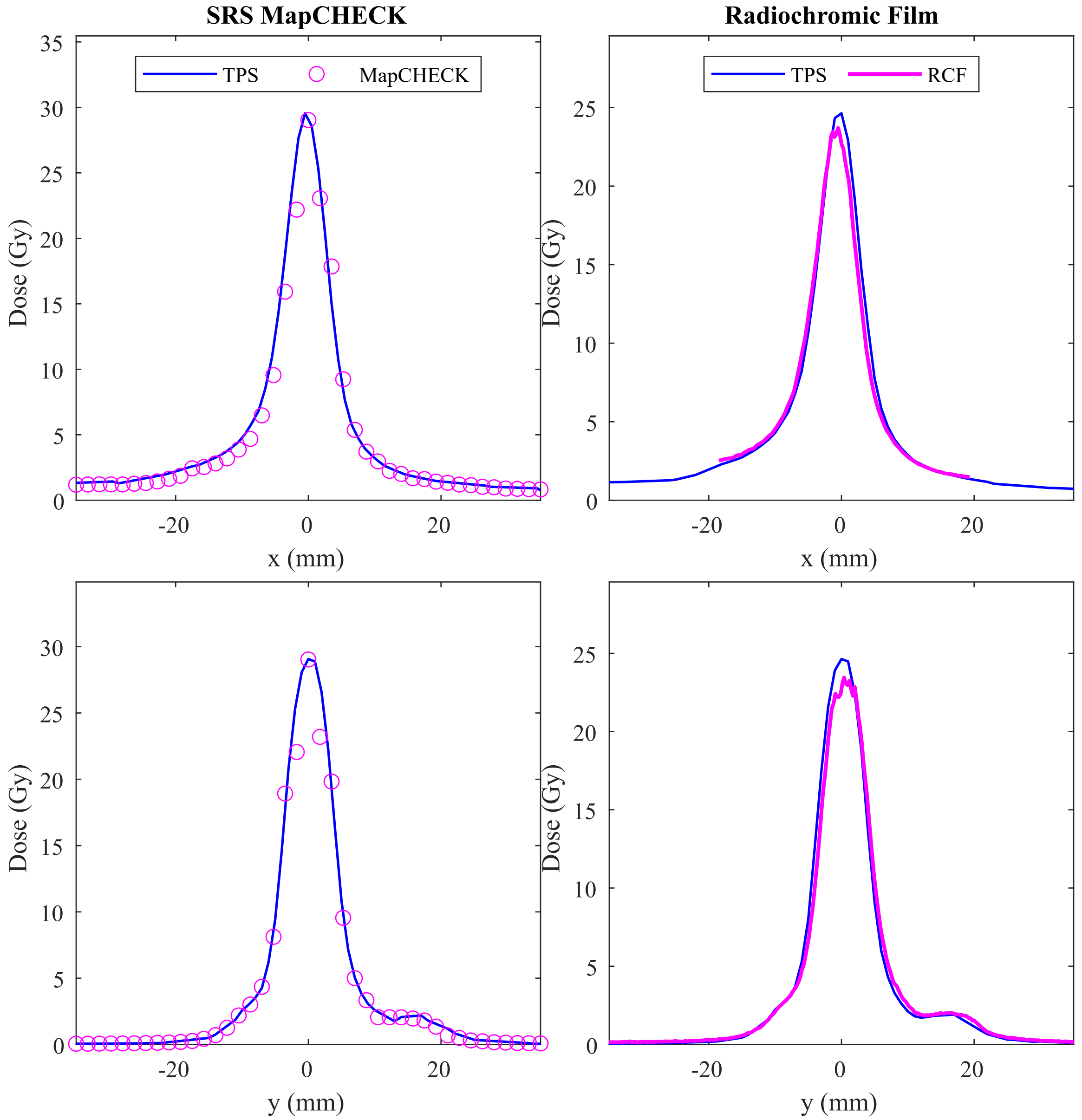

Measurement 71. Plan 44, 2 targets, equivalent diameter 9.2 mm

SRS MapCHECK fraction passing gamma 3%/1 mm = 100.0%

Radichromic film fraction passing gamma 3%/1 mm = 99.6%

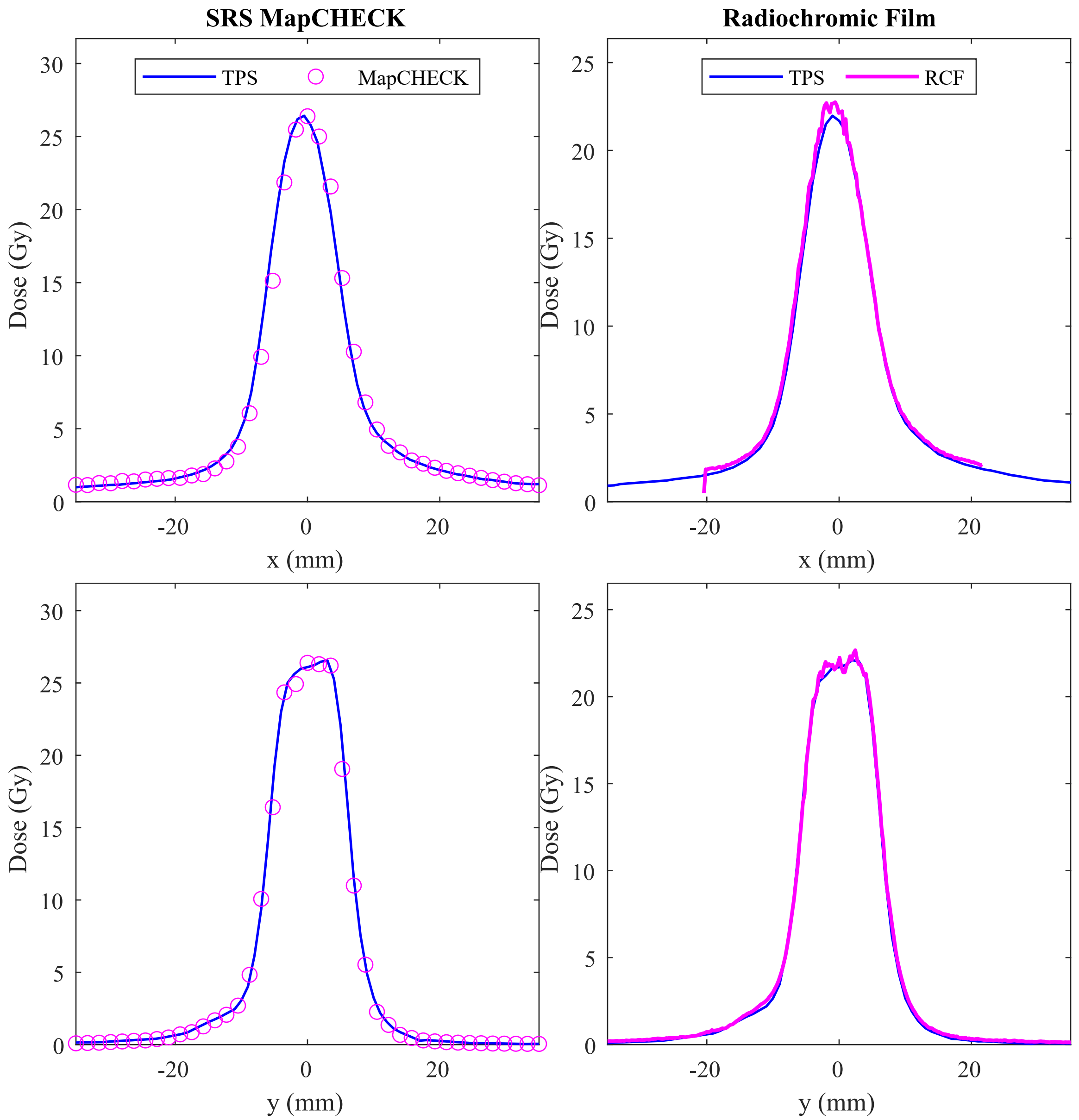

Measurement 72. Plan 45, single target, equivalent diameter 13.1 mm

SRS MapCHECK fraction passing gamma 3%/1 mm = 99.5%

Radichromic film fraction passing gamma 3%/1 mm = 99.7%

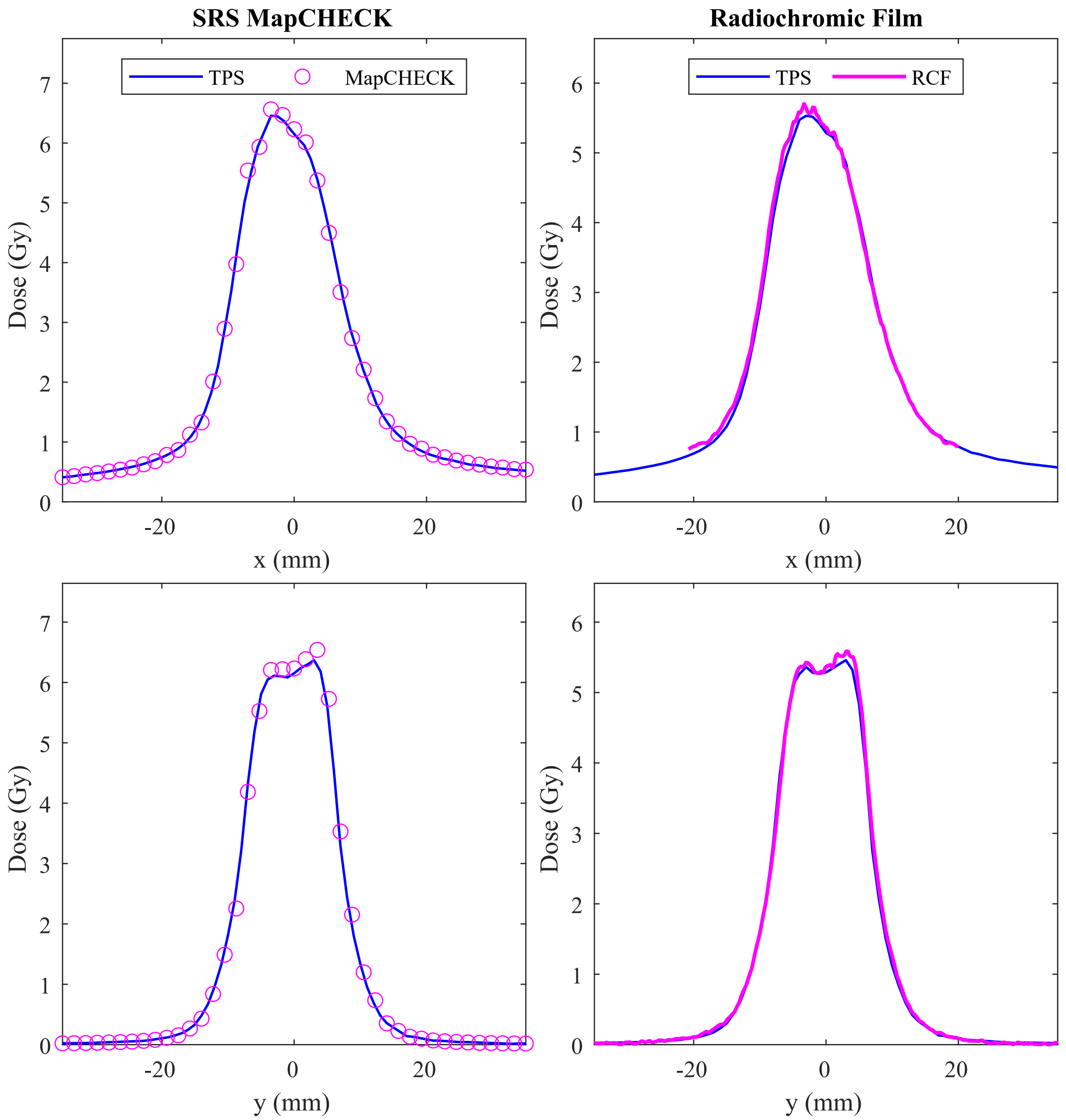

Measurement 73. Plan 46, 2 targets, equivalent diameter 4.2 mm

SRS MapCHECK fraction passing gamma 3%/1 mm = 100.0%

Radichromic film fraction passing gamma 3%/1 mm = 100.0%

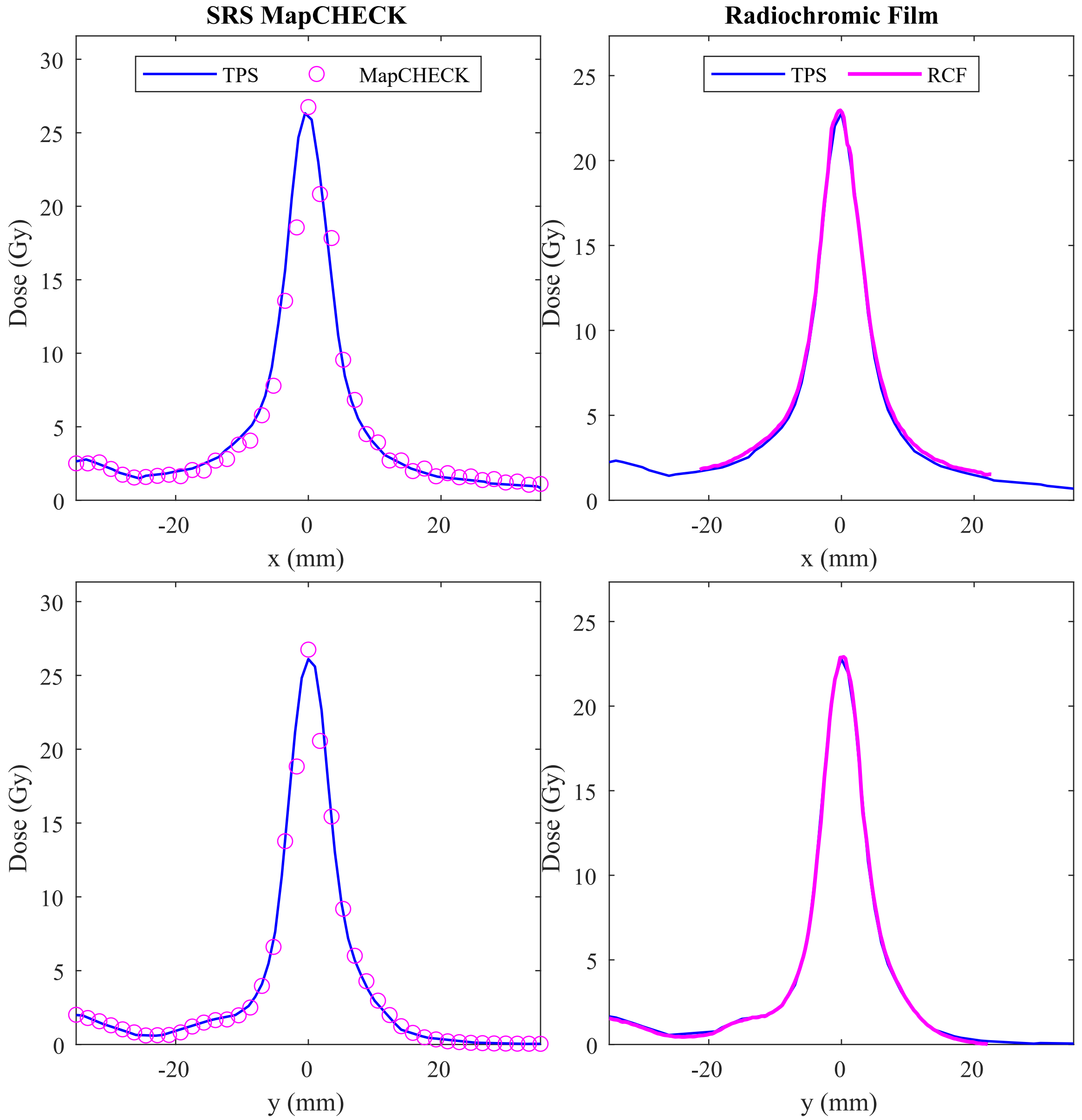

Measurement 74. Plan 46, 2 targets, equivalent diameter 8.8 mm

SRS MapCHECK fraction passing gamma 3%/1 mm = 100.0%

Radichromic film fraction passing gamma 3%/1 mm = 100.0%

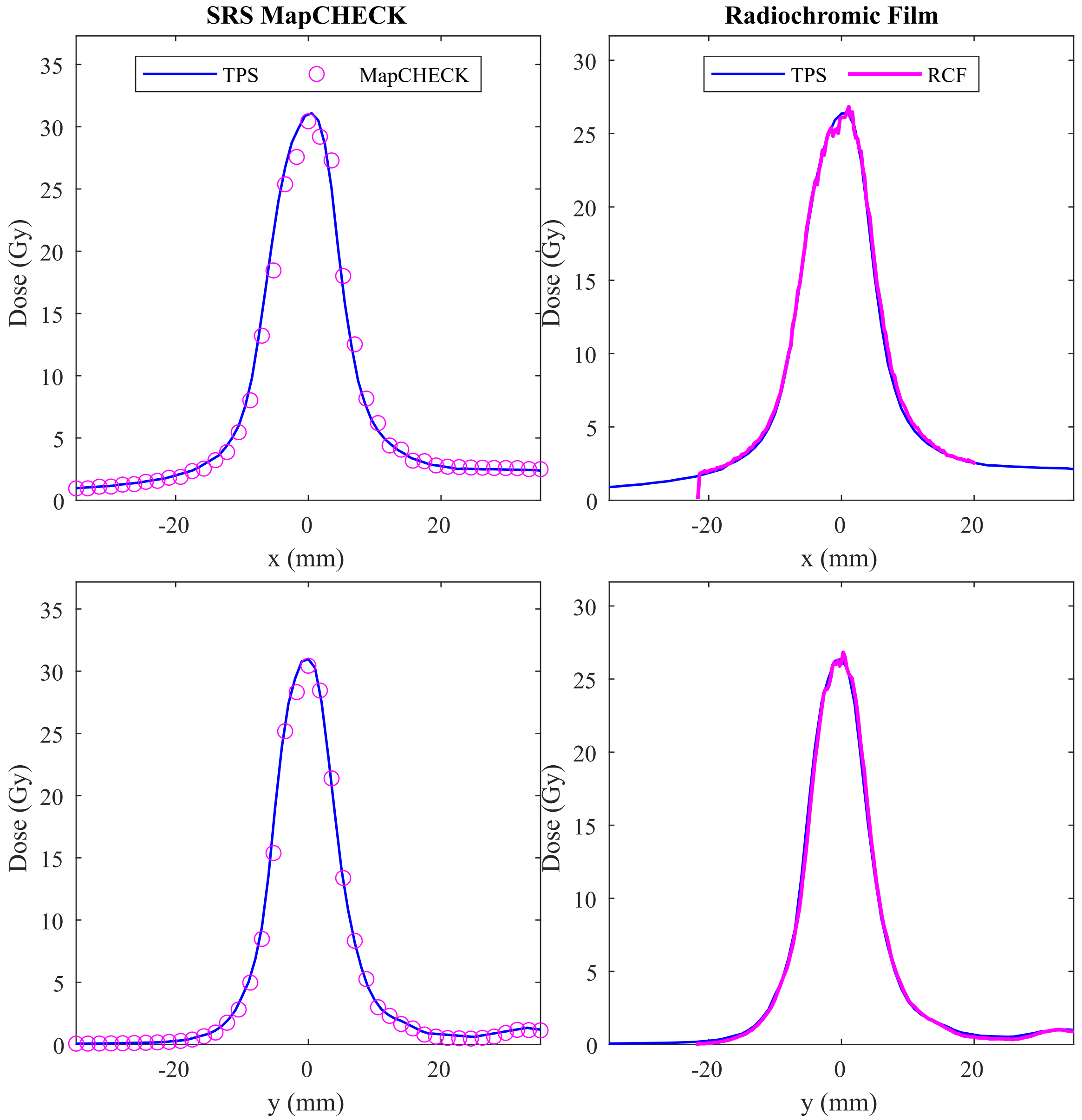

Measurement 75. Plan 47, single target, equivalent diameter 35.8 mm

SRS MapCHECK fraction passing gamma 3%/1 mm = 100.0%

Radichromic film fraction passing gamma 3%/1 mm = 97.2%

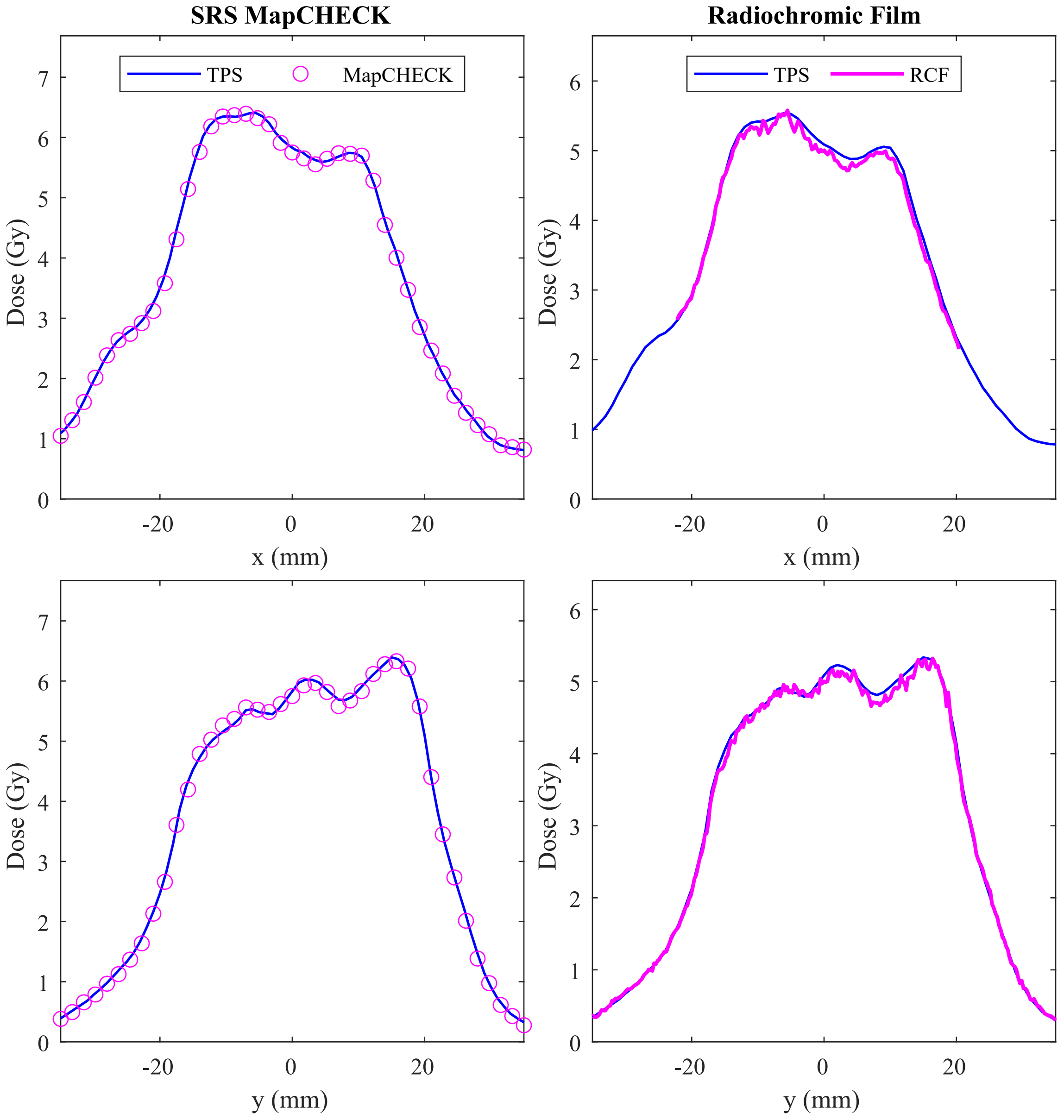

Measurement 76. Plan 48, 6 targets, equivalent diameter 2.7 mm

SRS MapCHECK fraction passing gamma 3%/1 mm = 100.0%

Radichromic film fraction passing gamma 3%/1 mm = 100.0%

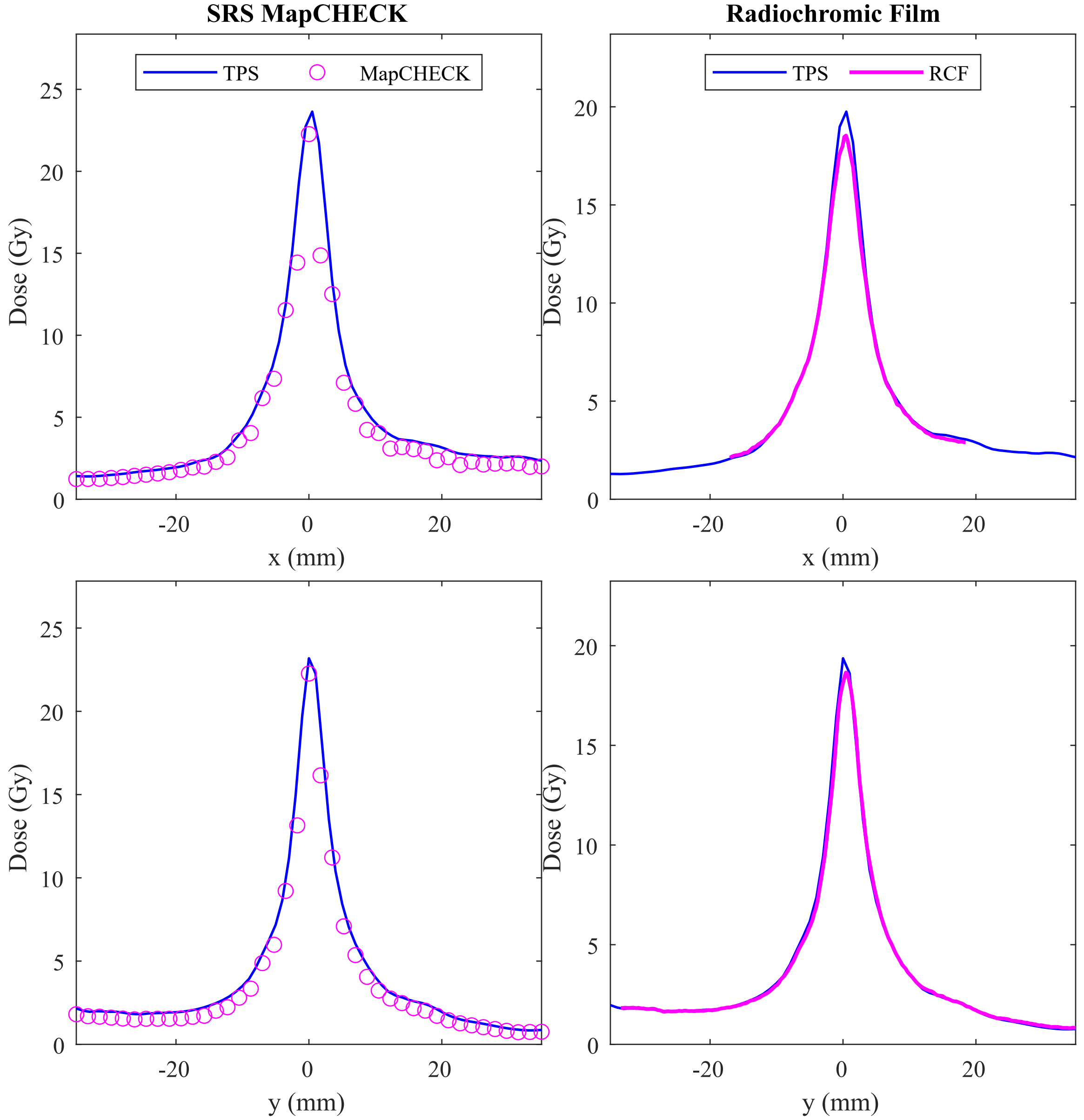

Measurement 77. Plan 48, 6 targets, equivalent diameter 6.6 mm

SRS MapCHECK fraction passing gamma 3%/1 mm = 100.0%

Radichromic film fraction passing gamma 3%/1 mm = 100.0%

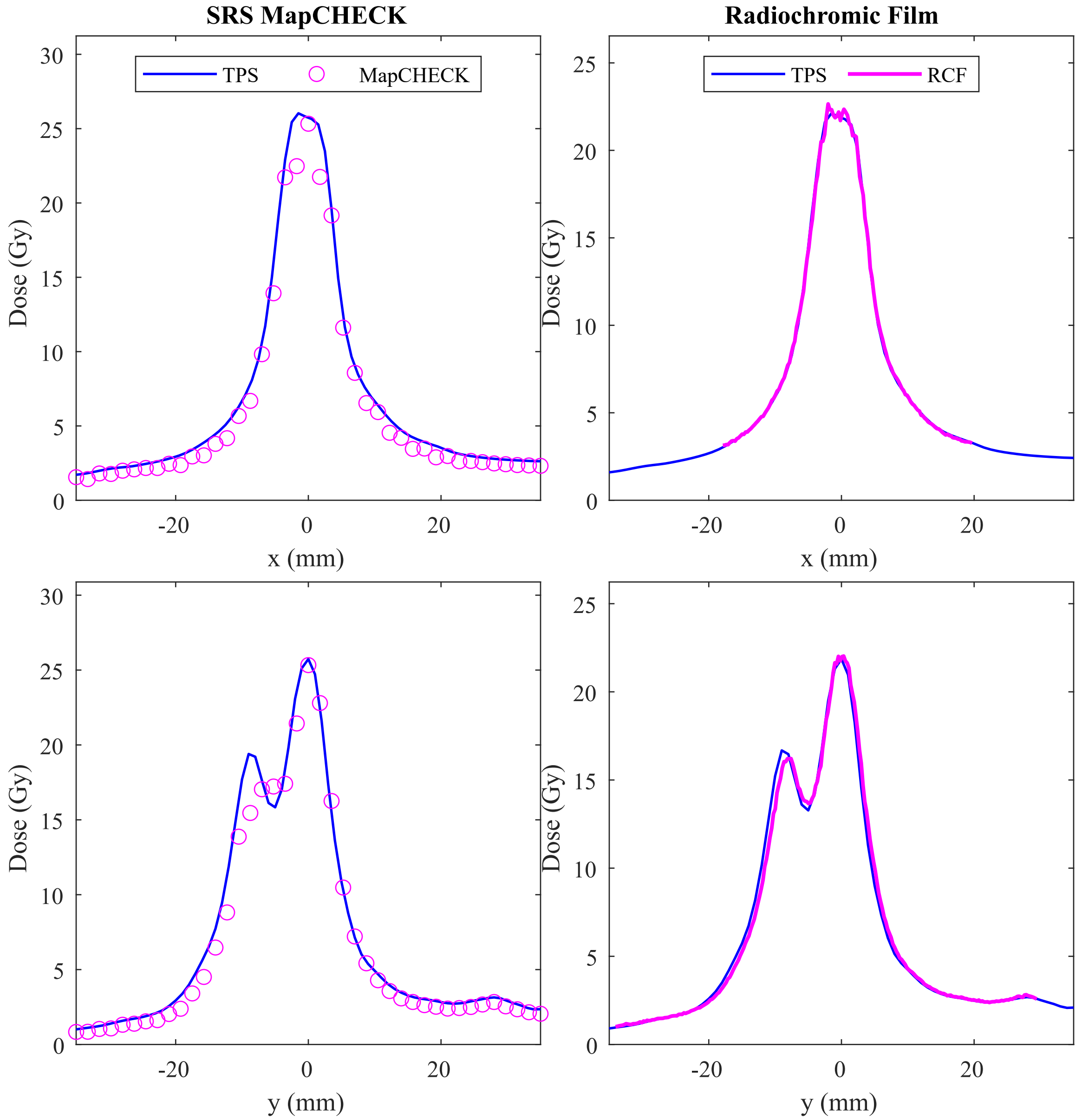

Measurement 78. Plan 49, 7 targets, equivalent diameter 3.5 mm

SRS MapCHECK fraction passing gamma 3%/1 mm = 100.0%

Radichromic film fraction passing gamma 3%/1 mm = 100.0%

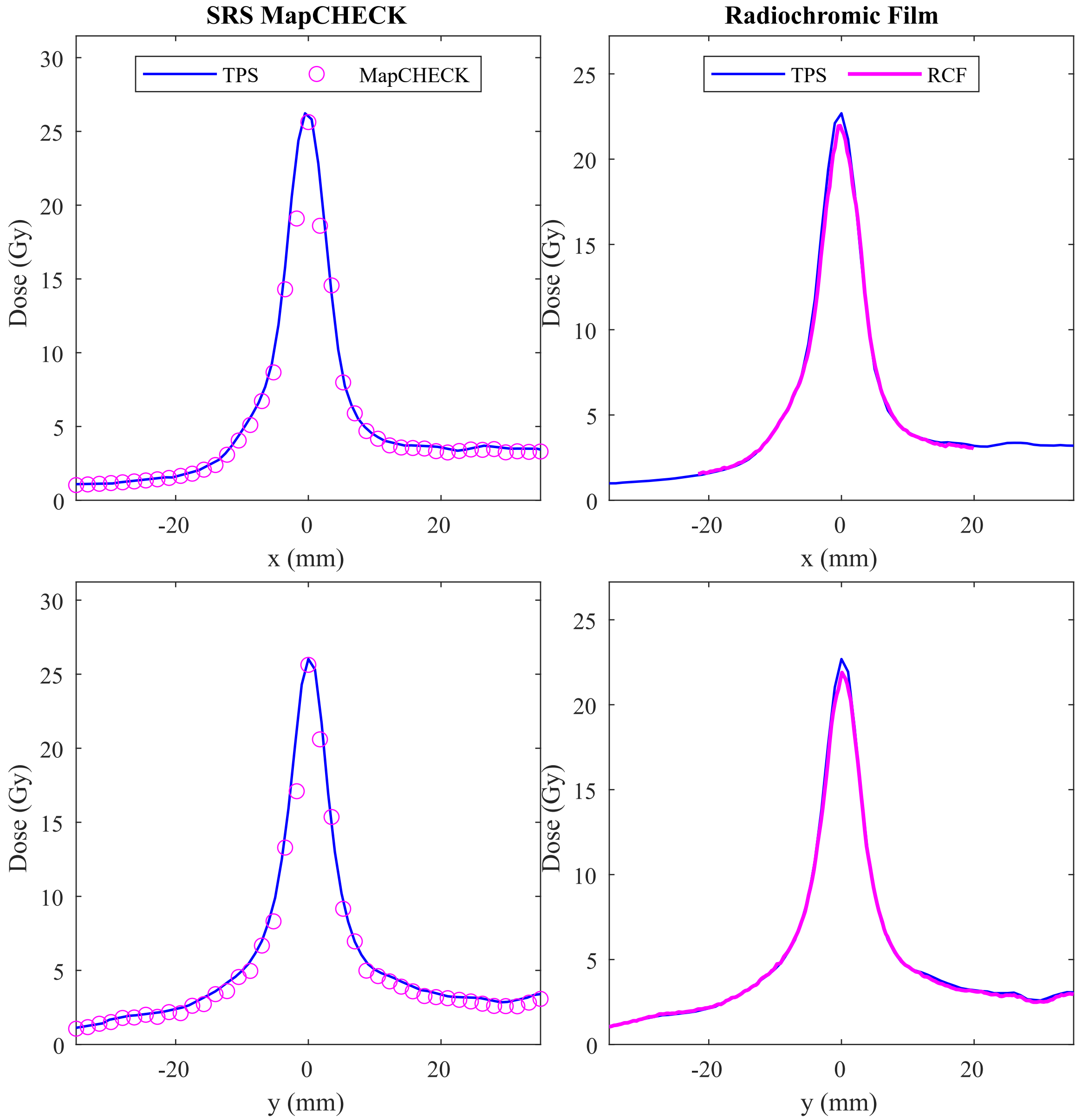

Measurement 79. Plan 49, 7 targets, equivalent diameter 44.7 mm

SRS MapCHECK fraction passing gamma 3%/1 mm = 90.3%

Radichromic film fraction passing gamma 3%/1 mm = 69.5%

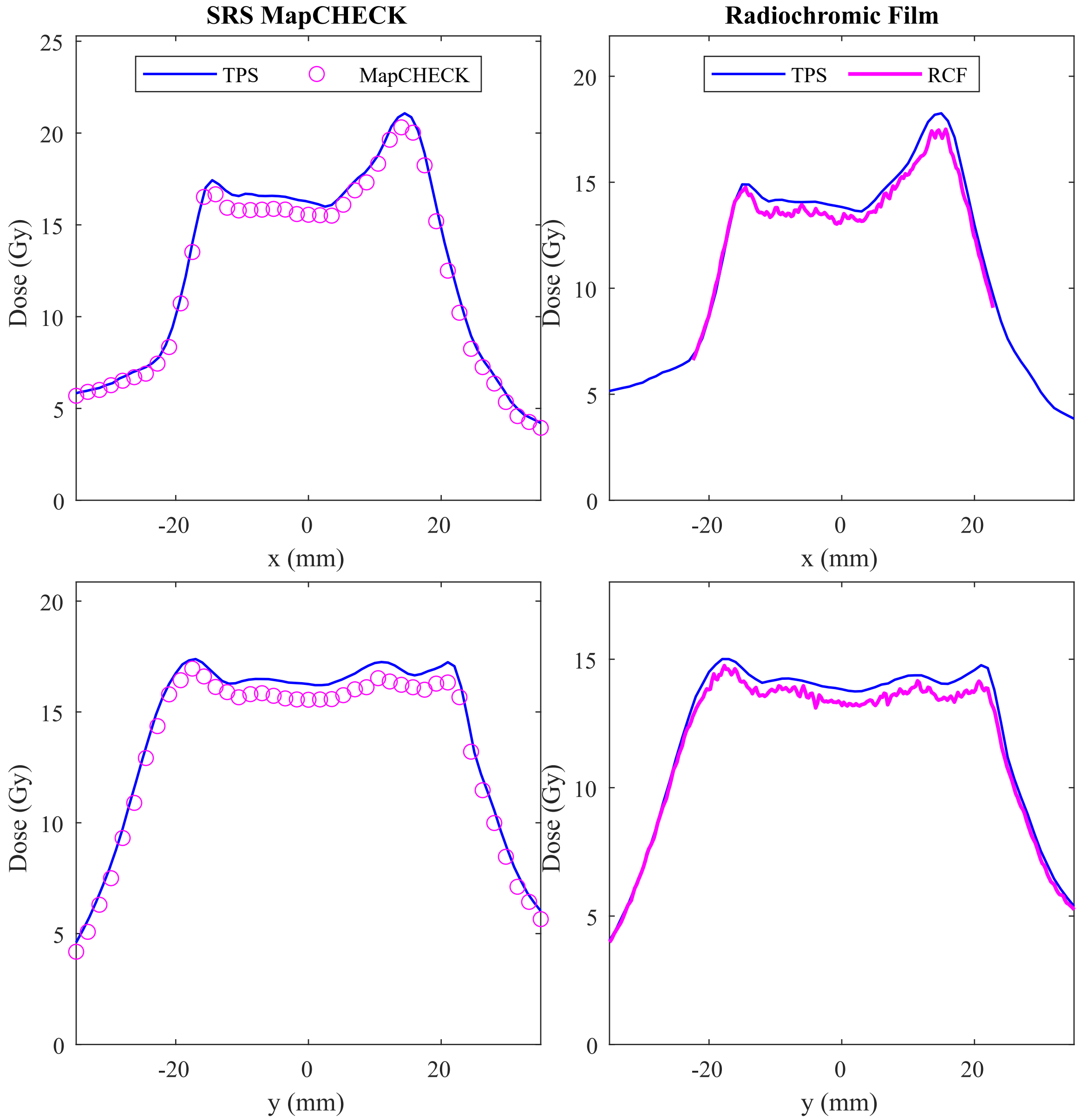

Measurement 80. Plan 50, single target, equivalent diameter 13.6 mm

SRS MapCHECK fraction passing gamma 3%/1 mm = 100.0%

Radichromic film fraction passing gamma 3%/1 mm = 85.7%

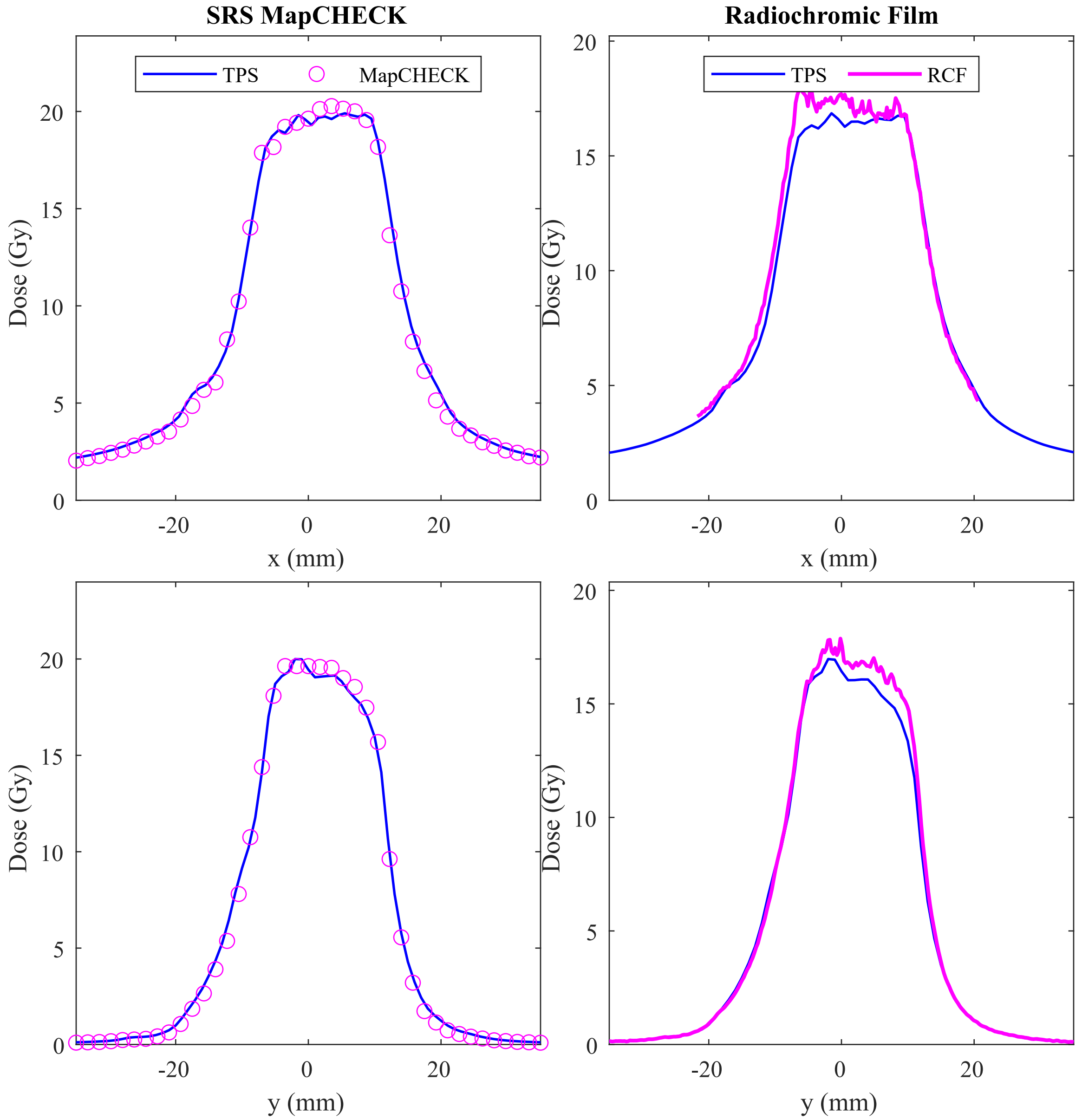

Measurement 81. Plan 51, single target, equivalent diameter 15.2 mm

SRS MapCHECK fraction passing gamma 3%/1 mm = 100.0%

Radichromic film fraction passing gamma 3%/1 mm = 95.0%

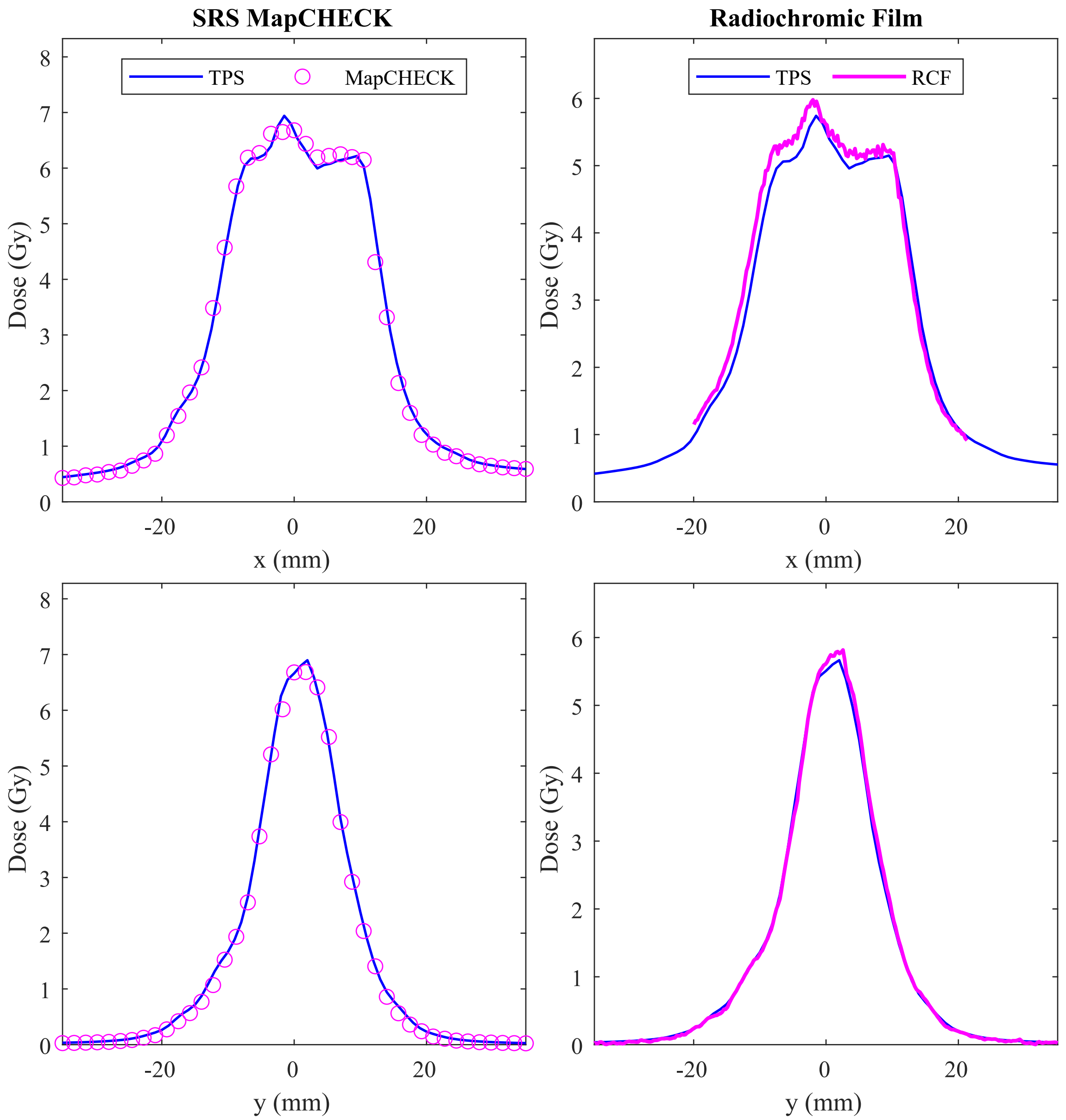

Measurement 82. Plan 52, single target, equivalent diameter 30.9 mm

SRS MapCHECK fraction passing gamma 3%/1 mm = 100.0%

Radichromic film fraction passing gamma 3%/1 mm = 95.4%

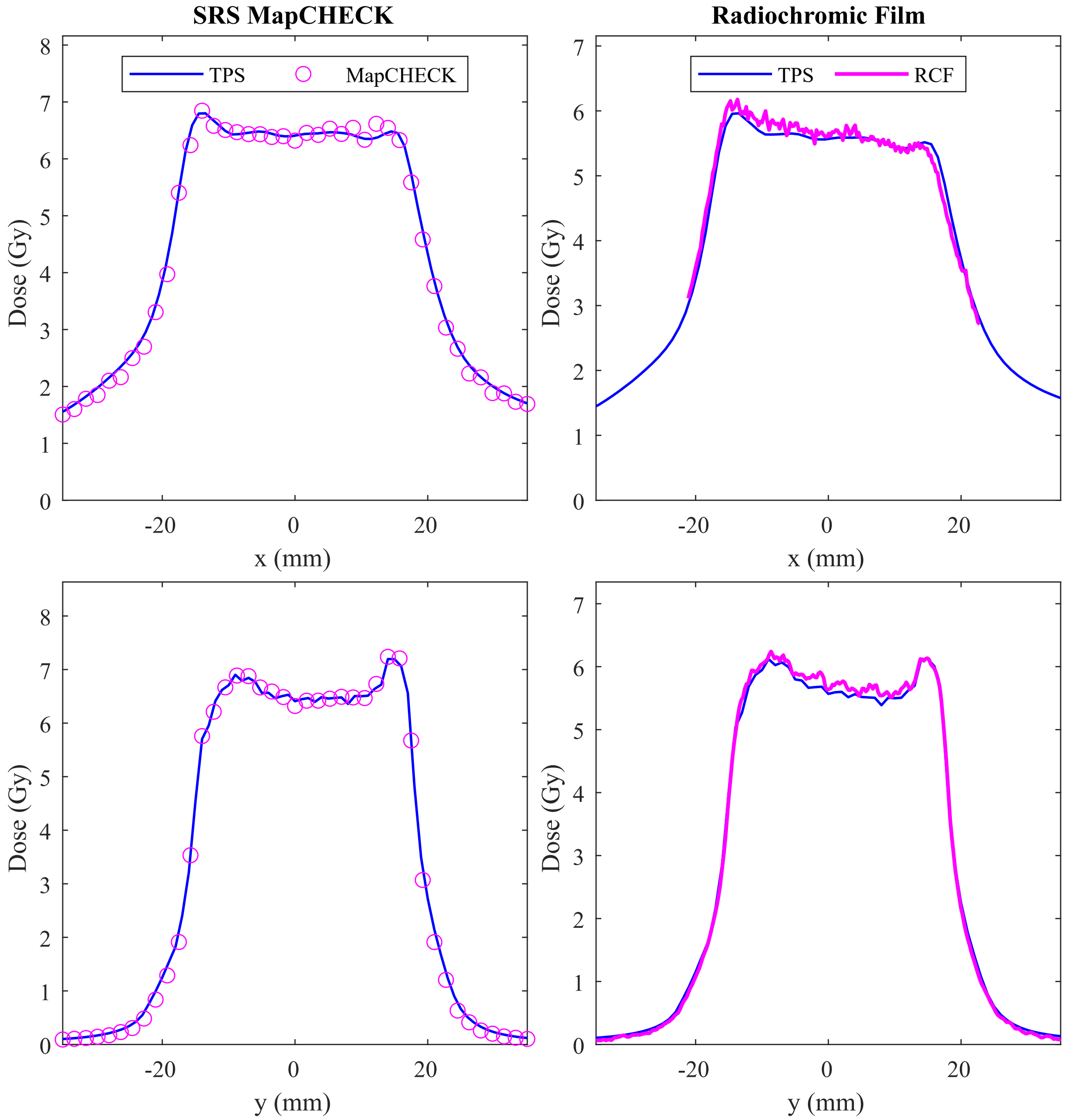

Measurement 83. Plan 53, single target, equivalent diameter 19.5 mm

SRS MapCHECK fraction passing gamma 3%/1 mm = 99.8%

Radichromic film fraction passing gamma 3%/1 mm = 99.8%

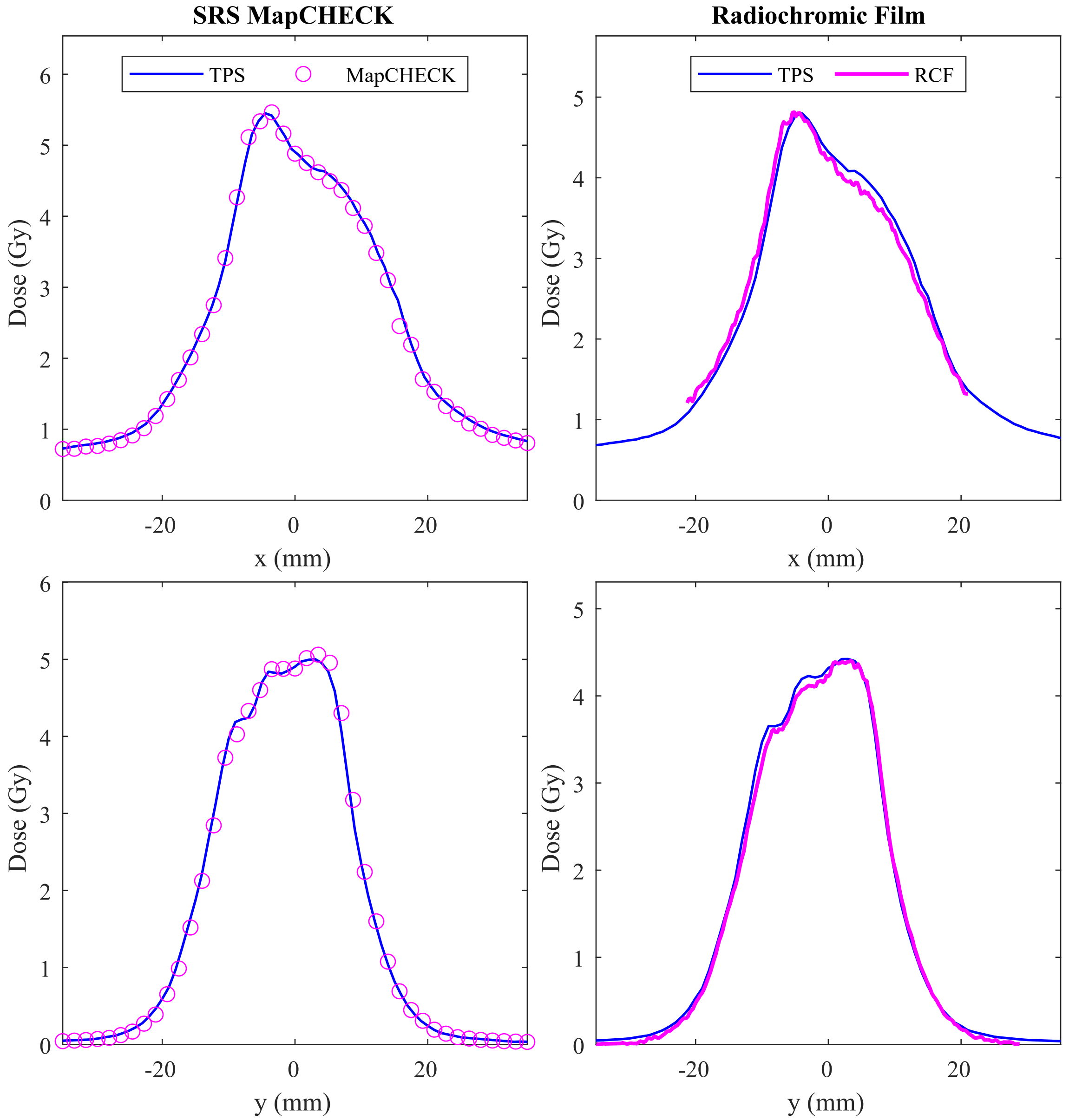

Measurement 84. Plan 54, single target, equivalent diameter 31.7 mm

SRS MapCHECK fraction passing gamma 3%/1 mm = 100.0%

Radichromic film fraction passing gamma 3%/1 mm = 99.0%

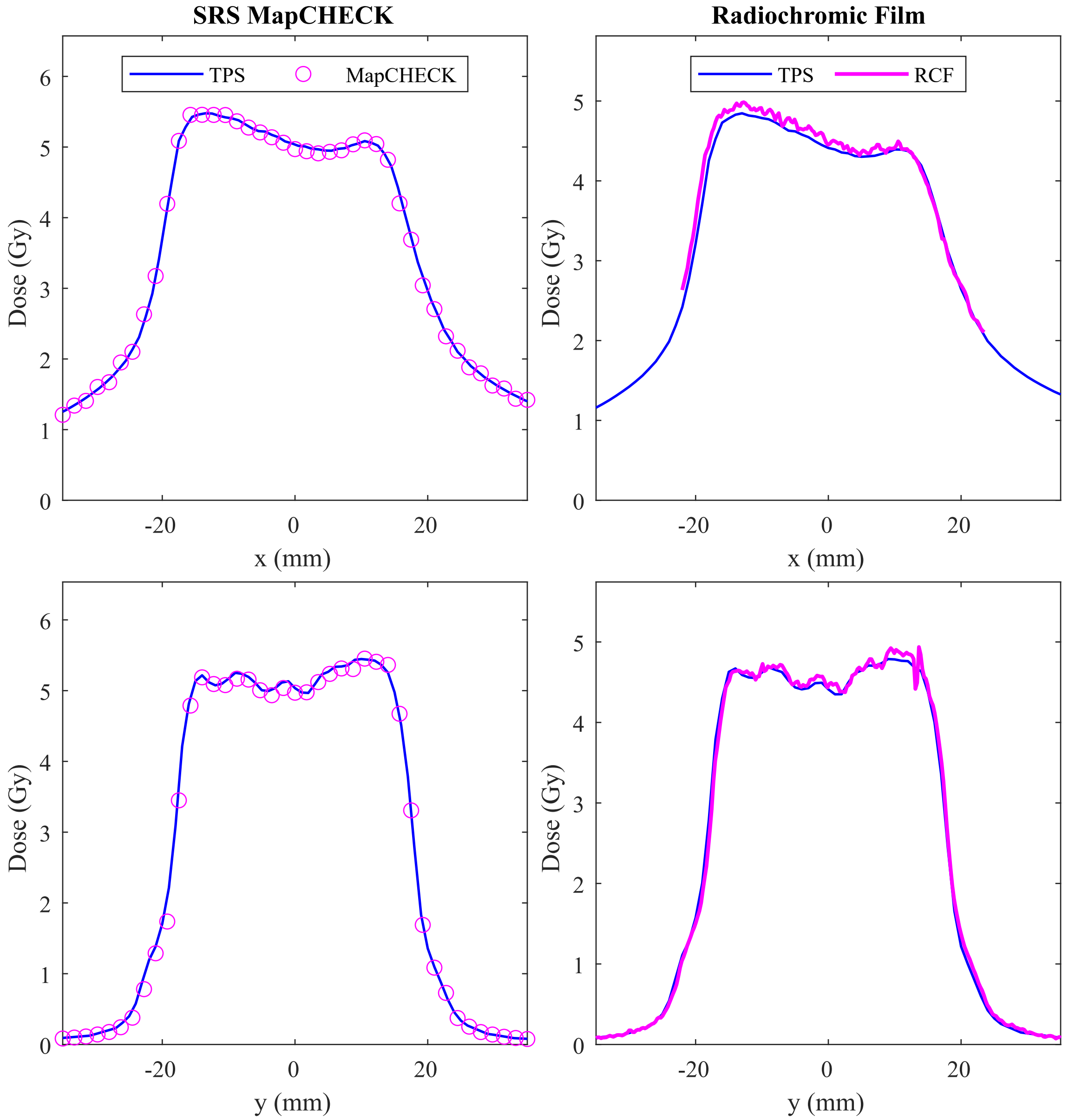

Measurement 85. Plan 55, single target, equivalent diameter 12.2 mm

SRS MapCHECK fraction passing gamma 3%/1 mm = 100.0%

Radichromic film fraction passing gamma 3%/1 mm = 96.1%

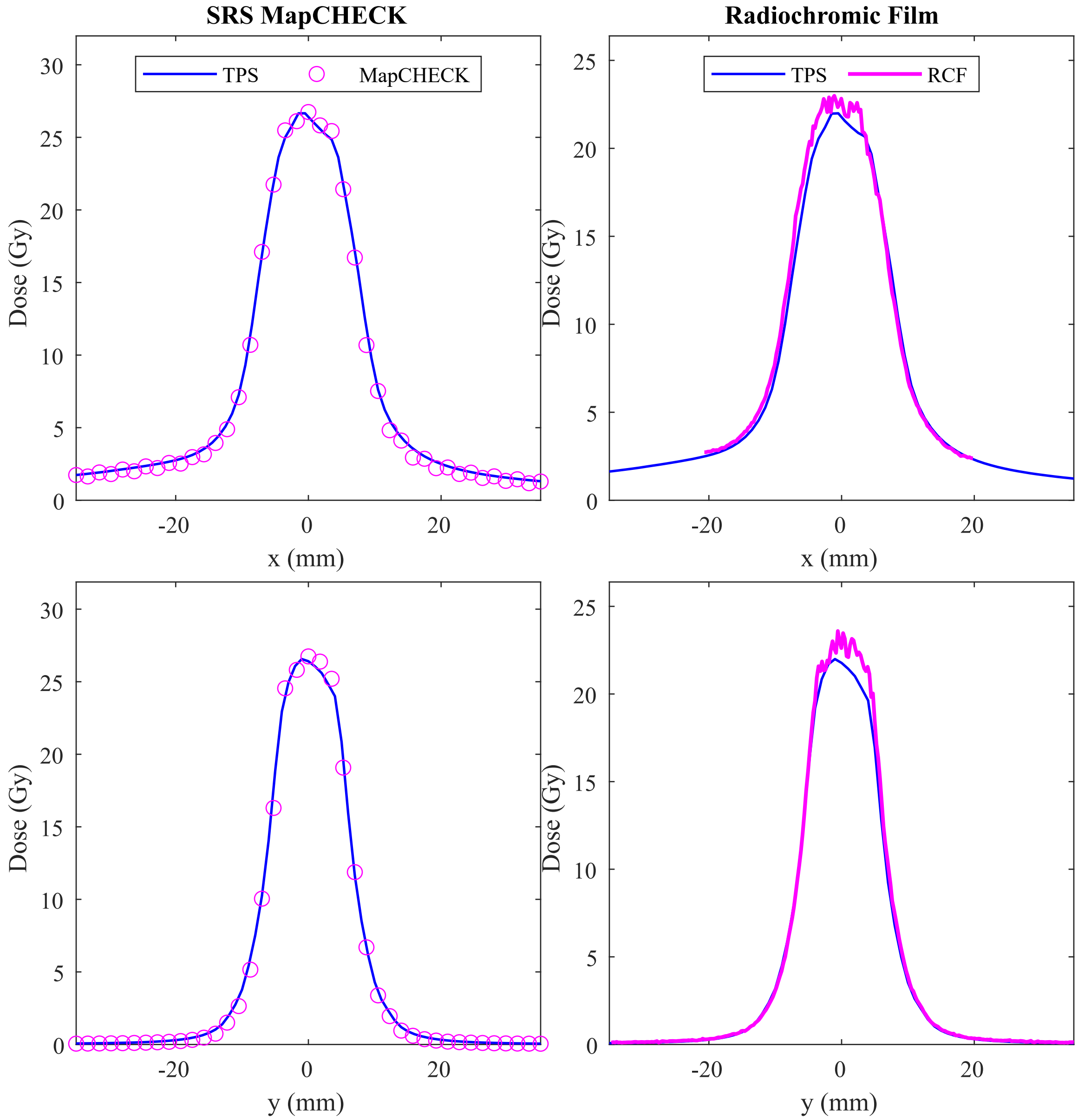

Measurement 86. Plan 56, single target, equivalent diameter 4.8 mm

SRS MapCHECK fraction passing gamma 3%/1 mm = 100.0%

Radichromic film fraction passing gamma 3%/1 mm = 100.0%

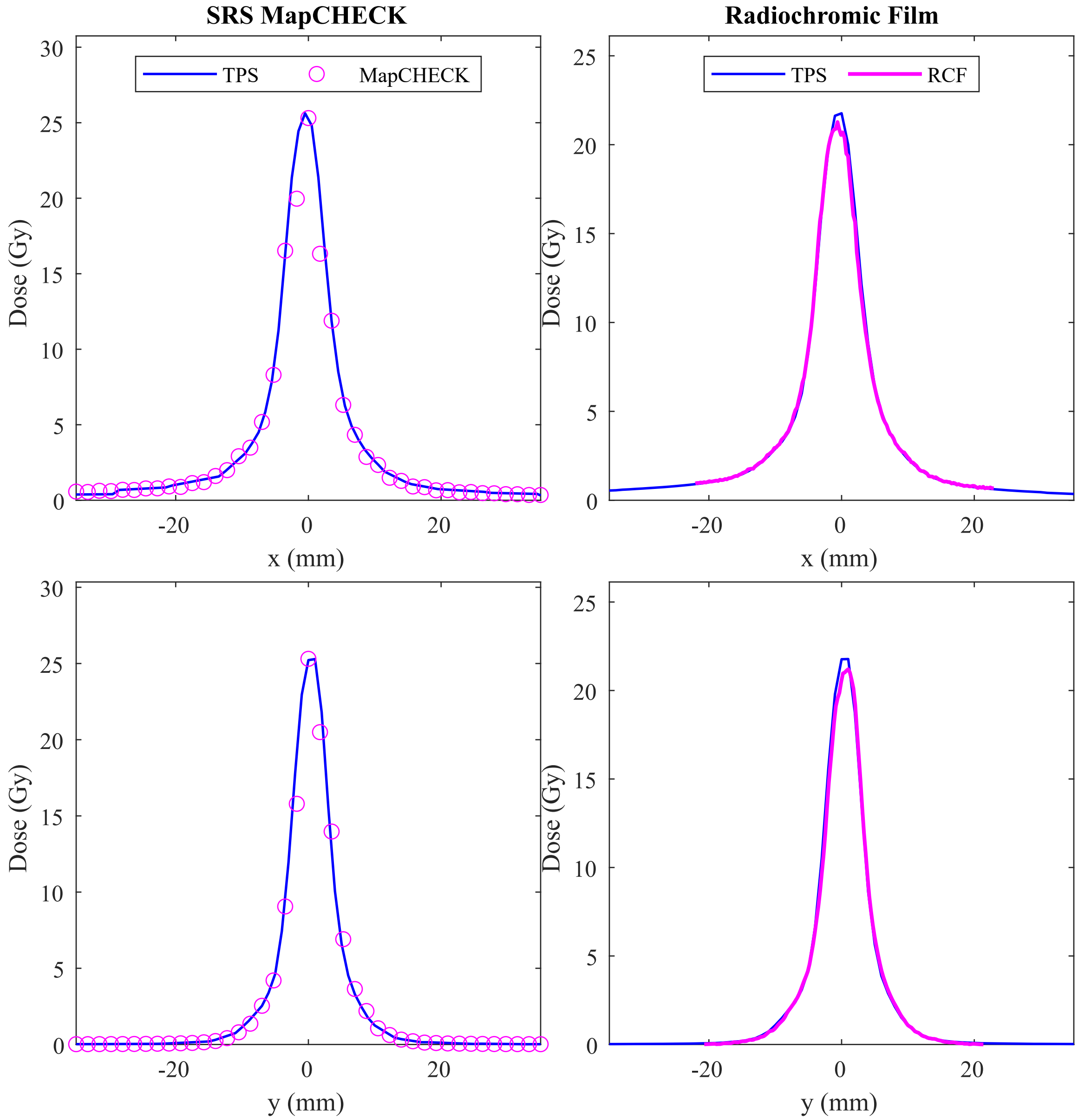

Measurement 87. Plan 57, single target, equivalent diameter 25.4 mm

SRS MapCHECK fraction passing gamma 3%/1 mm = 99.4%

Radichromic film fraction passing gamma 3%/1 mm = 97.0%

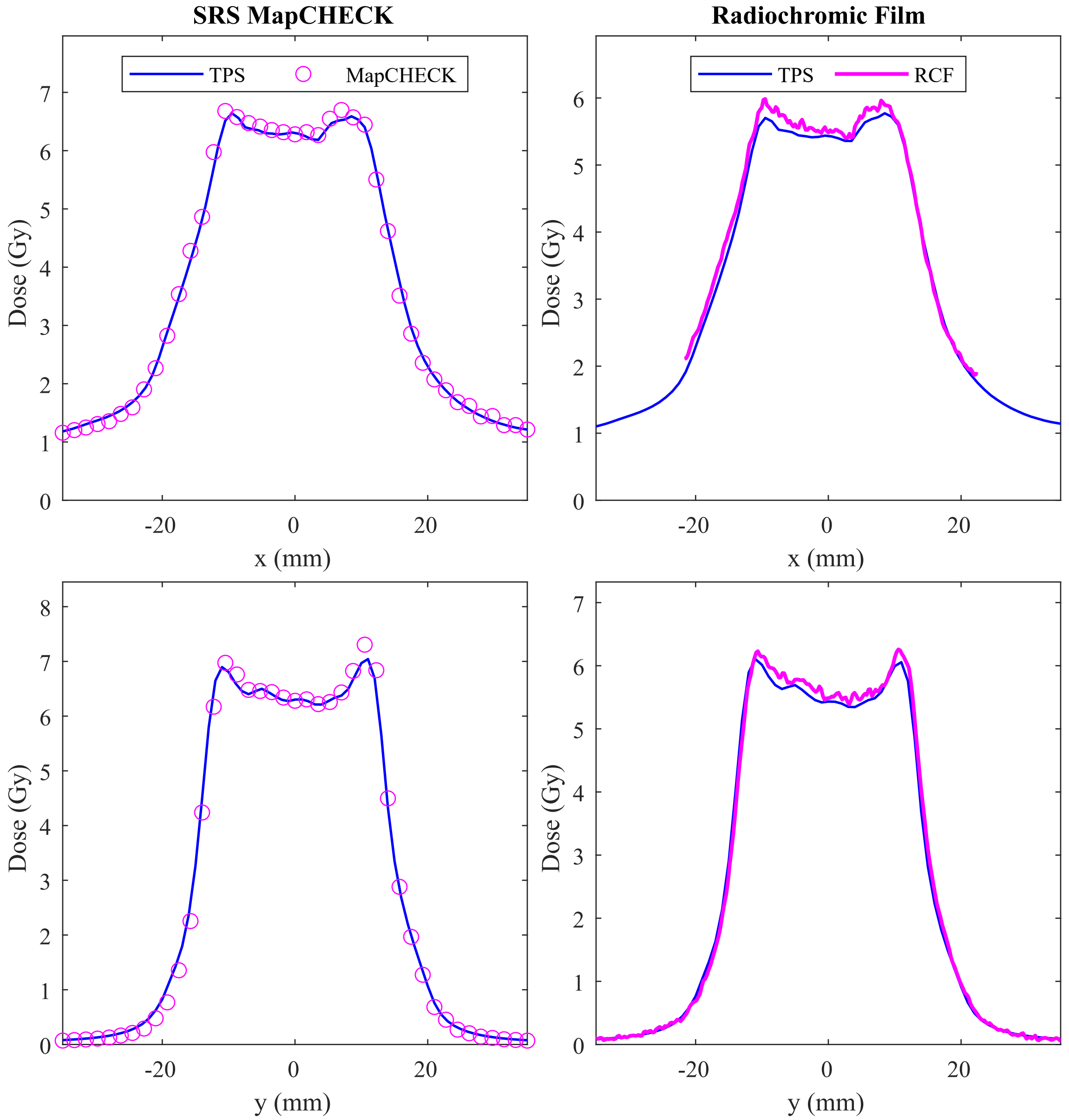

Measurement 88. Plan 58, single target, equivalent diameter 21.3 mm

SRS MapCHECK fraction passing gamma 3%/1 mm = 100.0%

Radichromic film fraction passing gamma 3%/1 mm = 98.8%

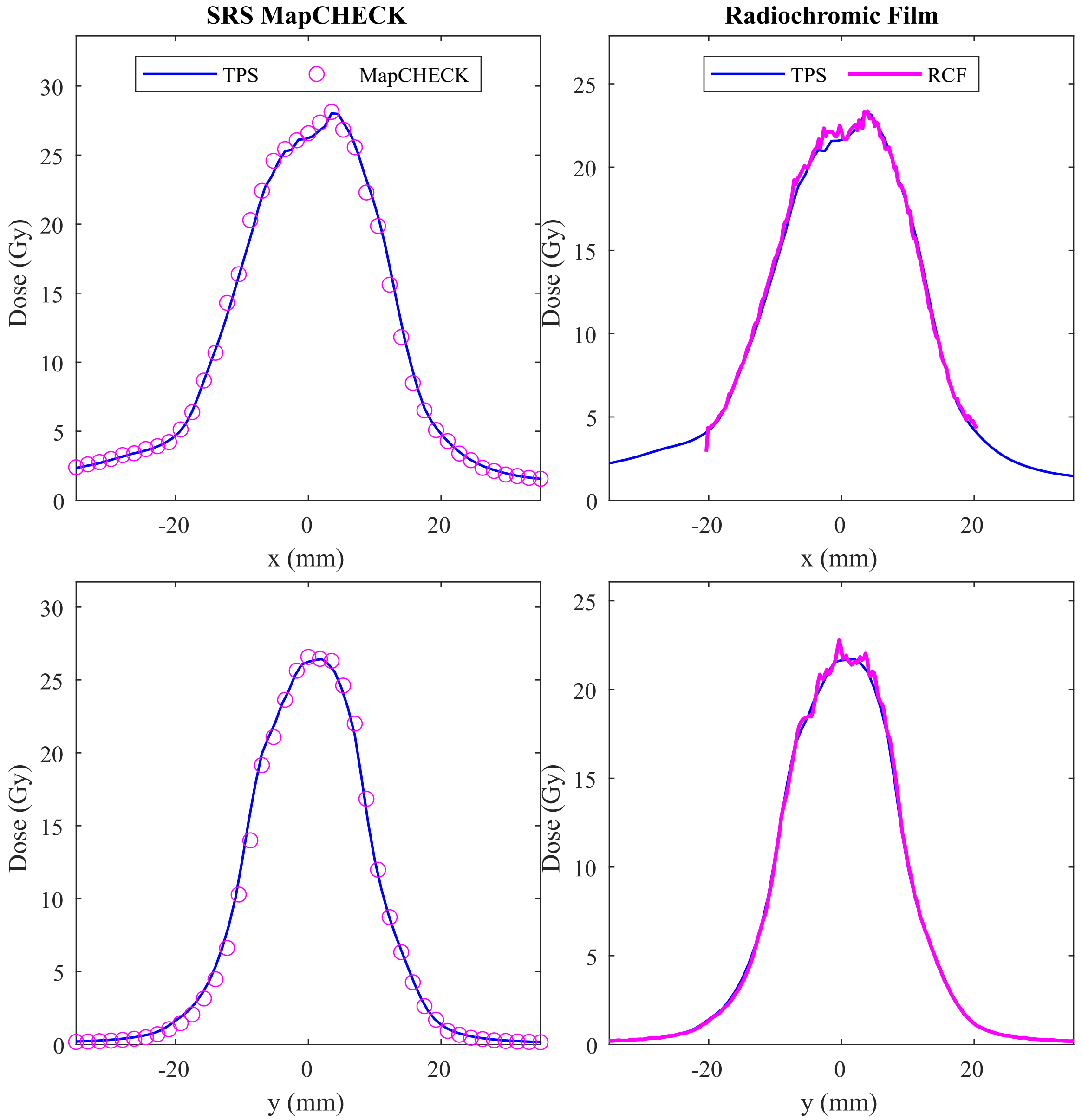

Measurement 89. Plan 59, single target, equivalent diameter 7.7 mm

SRS MapCHECK fraction passing gamma 3%/1 mm = 100.0%

Radichromic film fraction passing gamma 3%/1 mm = 99.7%

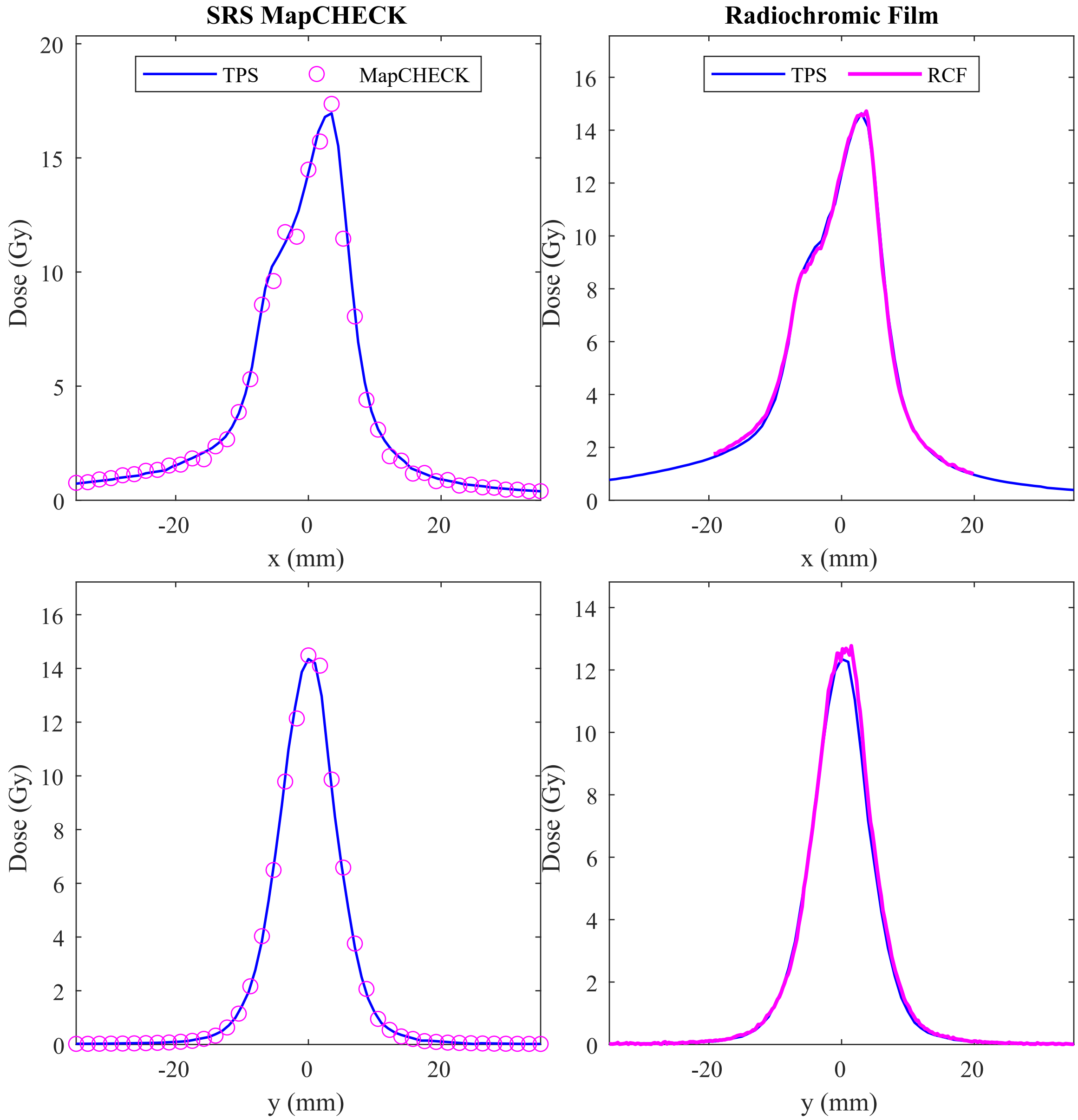

Measurement 90. Plan 60, single target, equivalent diameter 25.1 mm

SRS MapCHECK fraction passing gamma 3%/1 mm = 100.0%

Radichromic film fraction passing gamma 3%/1 mm = 97.7%

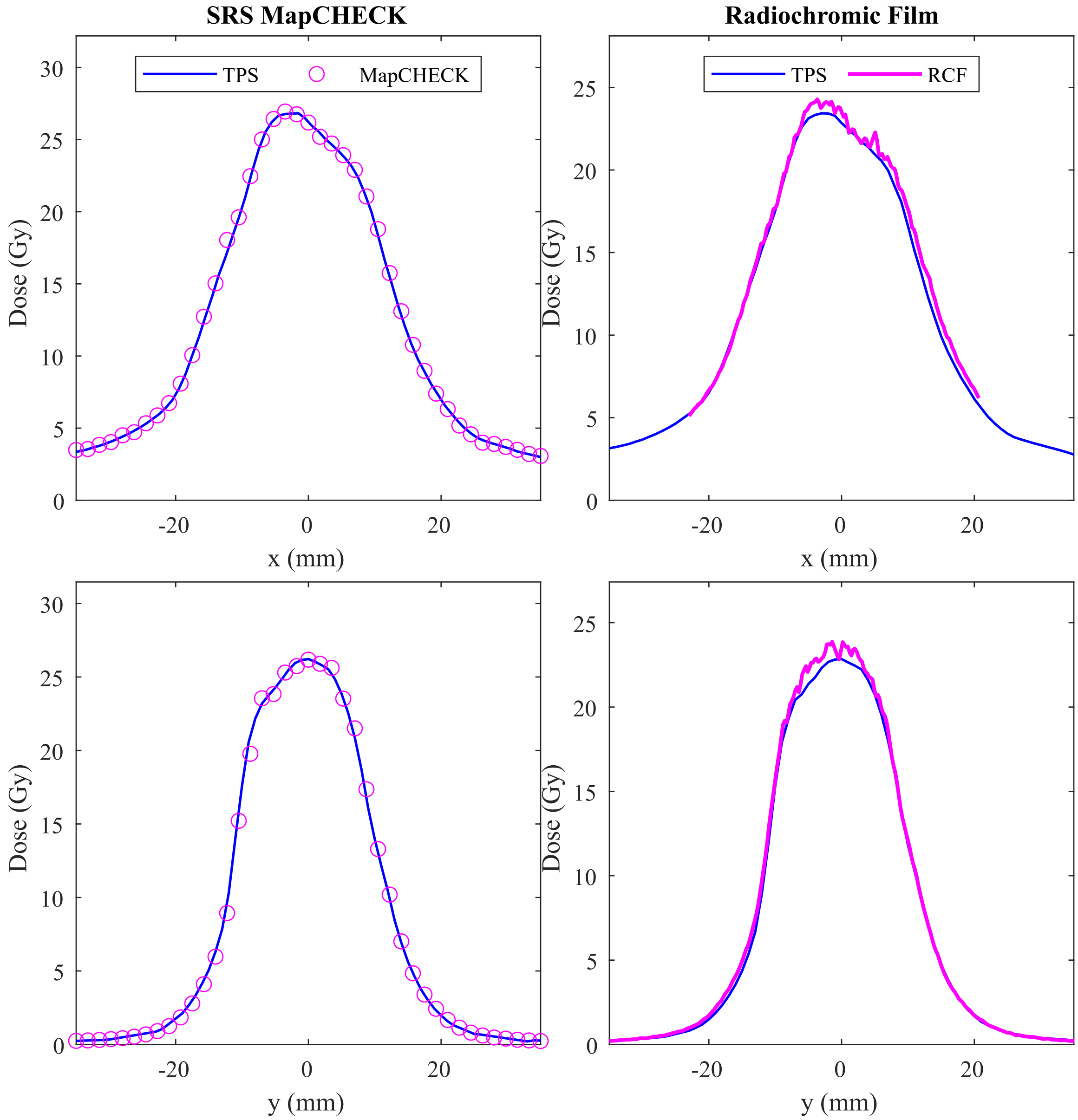

Supplement: Supplementary file 1 — Supporting information [file ACM2-22-203-s002.pdf]
